# Biomechanical and Musculoskeletal Measurements as Risk Factors for Running-Related Injury in Non-elite Runners: A Systematic Review and Meta-analysis of Prospective Studies

**DOI:** 10.1186/s40798-022-00416-z

**Published:** 2022-03-07

**Authors:** Benjamin Peterson, Fiona Hawke, Martin Spink, Sean Sadler, Morgan Hawes, Robin Callister, Vivienne Chuter

**Affiliations:** 1 School of Health Sciences, College of Health, Medicine and Wellbeing, University of Newcastle, Central Coast Campus, Ourimbah, NSW 2258 Australia; 2 Department of Podiatry, School of Health, Medical and Applied Sciences, CQUniversity, Rockhampton, QLD 4701 Australia; 3 School of Biomedical Sciences and Pharmacy, College of Health, Medicine and Wellbeing, University of Newcastle, Callaghan Campus, Callaghan, NSW 2308 Australia; 4 School of Health Sciences, Western Sydney University, Campbelltown Campus, Sydney, NSW 2560 Australia

**Keywords:** Running-related injury, Risk factor, Biomechanics, Screening, Systematic review, Meta-analysis

## Abstract

**Background:**

Running-related injury (RRI) is highly prevalent among recreational runners and is a key barrier to participation. Atypical lower limb alignment and mechanical function have been proposed to play a role in development of lower extremity injury. The purpose of this study was to investigate relationships between incidence of running-related injury (RRI) in non-elite runners with biomechanical and musculoskeletal variables.

**Methods:**

A systematic review and meta-analysis of prospective studies. Published research indexed in MEDLINE, EMBASE, CINAHL, SPORTDiscus, AMED, and The Cochrane library until 13th January 2021, grey literature, and reference lists of included studies were screened to identify prospective studies of non-elite adult runners that measured a relationship between biomechanical or musculoskeletal measures and incidence of RRI.

**Results:**

Thirty studies (3404 runners), testing over 100 discrete biomechanical and musculoskeletal risk factors for RRI, were included. Nineteen studies were pooled in twenty-five separate meta-analyses. Meta-analysis of four studies detected significantly less knee extension strength among runners who developed a RRI (SMD − 0.19, 95% CI − 0.36 to − 0.02, *p* = 0.03), though this may not be clinically important. A meta-analysis of two studies detected significantly lower hip adduction velocity among runners who developed a RRI (MD − 12.80, 95% CI − 25.22 to − 0.38, *p* = 0.04). Remaining meta-analyses found no significant relationship between biomechanical or musculoskeletal variables and RRI.

**Conclusion:**

This systematic review and meta-analysis found the currently available literature does not generally support biomechanical or musculoskeletal measures as risk factors for RRI in non-elite runners. While meta-analysis findings for knee extension strength and hip adduction velocity as risk factors for RRI were statistically significant, the associated trivial to small effects sizes suggest these findings should be treated with caution. Until further evidence emerges, recommendations for injury prevention in non-elite runners cannot be made based on biomechanical and musculoskeletal measurements alone.

**Supplementary Information:**

The online version contains supplementary material available at 10.1186/s40798-022-00416-z.

## Key Points

- Meta-analyses of included prospective studies found no meaningful differences in biomechanical or musculoskeletal measurements between non-elite runners who did and did not prospectively develop a RRI.
- A trivial reduction in knee extension strength identified among runners who prospectively developed a RRI, and a difference in mean hip adduction velocity, between prospectively injured and non-injured runners require further investigation in future longitudinal studies.
- Biomechanical factors reported as significant predictors of injury in recent systematic reviews need to be interpreted with caution. Other kinematic and kinetic variables recently identified as risk factors for running-related injury have been demonstrated in single studies and require further investigation before being applied clinically.

## Background

Running-related injury (RRI) is defined as running-related musculoskeletal pain in the lower limbs that causes a restriction or stoppage of running (distance, speed, duration, or training) for at least seven days or three consecutive scheduled training sessions, or that requires the runner to consult a physician or other health professional [1]. Recent systematic reviews have reported a mean incidence of RRI of between 37 [2] and 40% [3]; however rates as high as 79% during a six-month follow-up have been reported [4]. Predominantly, RRI affects the more distal portion of the lower limb, with 70% occurring at or below the knee [5]. A systematic review of nine articles identified the most common RRIs to be patellofemoral pain, Achilles tendonitis, iliotibial band syndrome and plantar fasciitis [6]. RRI affects event preparation [7], and is associated with psychological distress [8], financial expense [7], and reduced motivation to return to running [9].

While risk factors for RRI are not well understood, there is some evidence that previous injury [10] and particular training errors, such as pronounced increases in training volume or intensity, may be contributors [11]; however the evidence for this relationship is inconsistent [12]. Atypical lower limb alignment and biomechanical function are proposed to play a role in the development of lower limb injury [13], and may contribute to development of RRI. Previous investigations of biomechanical and musculoskeletal risk factors for RRI have included measures of muscle strength [14–18], joint range of motion (ROM) [4, 16, 19], lower limb alignment characteristics [4, 16, 17, 20, 21], plantar pressure analysis [22, 23], running kinetics [16, 24, 25], and three-dimensional (3D) running kinematics [16, 26–29]. The findings of these studies have generally provided conflicting or inconclusive results.

Most runners are not elite, yet studies often focus on or include elite runners [30, 31]. This may compromise our ability to get a clear understanding of risk factors for RRI in non-elite runners. The authors are unaware of any systematic review on RRI that comprises only prospective studies of adult non-elite runners and includes all currently available research of biomechanical and musculoskeletal risk factors for RRI. A number of reviews have included study designs that are not prospective [32–35] despite this being the most appropriate methodology for determining causality for sports injuries [36]. Recent reviews by Ceyssens et al. [37] and Christopher et al. [38] performed syntheses of research of biomechanical and musculoskeletal risk factors for RRI, respectively, but they did not perform meta-analyses. Further, these reviews included non-adult participants, as did a recent review by Vannatta et al. [39], which performed pooled analyses, but excluded novice runners while including high calibre (National Collegiate Athletic Association (NCAA) Division 1) runners [39]. The review by Vannatta et al. [39] identified differences in risk factors between recreational and high-calibre runners, supporting the need to better consolidate research of risk factors for RRI in non-elite runners. Therefore, there is an absence of meta-analyses of musculoskeletal measures as risk factors for RRI, and existing meta-analyses of biomechanical risk factors for RRI have not included novice and recreational runners.

The aim of this systematic review and meta-analyses of available prospective evidence was to evaluate biomechanical and musculoskeletal risk factors for RRI in non-elite adult runners.

## Methods

This systematic review was conducted in accordance with the Preferred Reporting Items for Systematic Reviews and Meta-Analyses (PRISMA) statement [40], and the protocol was prospectively registered (PROSPERO ID: CRD42018089392).

### Search Strategy

An electronic database search of MEDLINE, EMBASE, CINAHL, SPORTDiscus, AMED, and The Cochrane library was conducted from inception to the 13th January 2021. The search strategy applied to Ovid MEDLINE is presented in “Appendix 1” and was adapted for each database. No language or publication restrictions were applied. Authorship and results were not masked. Reference lists of included studies were manually screened for other potentially eligible studies, as were sources of grey literature, including conference proceedings, dissertations, and editorials.

### Eligibility Criteria

Prospective studies of RRI incidence including non-injured adult runners were eligible for inclusion. Studies of elite and sub-elite athletes (including NCAA Division 1 and 2 cross-country runners), sprinters, middle distance runners (800–3000 m), and military personnel were not eligible for inclusion. With the absence of consensus for the definition of elite and sub-elite athletes and the presence of performance-based scholarships within NCAA division 1 and 2 institutions [41], runners from NCAA divisions 1 and 2 but not division 3 institutions were excluded with the intention of reducing heterogeneity within our population, without totally excluding collegiate runners in an attempt to minimise selection bias. Likewise, if a runner’s status as ‘non-elite’ could not be determined based on a study’s eligibility criteria (such as a study of ‘competitive runners’), corresponding authors were contacted to confirm whether any participants in their sample were considered ‘elite or sub-elite athletes’. If a study included some elite athletes, corresponding authors could provide reanalysed data or raw data which included only runners who were eligible for this review. Where the competition level of ‘competitive’ runners in a particular study could not be determined after contacting a corresponding author, that study was not excluded from the review entirely, but was not included in any relevant pooled analyses to avoid unintended contamination effects.

Studies were eligible for inclusion regardless of the specific type (diagnosis) or definition of RRI used, as studies reporting on the consensus definition of RRI [1] are very few in number. Eligible studies had to conduct a biomechanical (e.g., walking or running kinematics/kinetics) and/or musculoskeletal assessment (e.g., muscle strength, joint ROM, skeletal alignment) at baseline and followed runners over time to track RRI incidence. Studies occurring during start-to-run programs or event preparation programs were eligible for inclusion. Any follow-up duration was acceptable, except for studies of injury incidence during a single event. Studies in which participants were provided with any non-running intervention, such as a footwear or injury prevention program (including gait retraining programs), were excluded.

Authors were contacted via email where clarification of participant eligibility, e.g. competition level, was required. Where no response was received, and uncertainty about participant eligibility remained, the study was included in this review but omitted from the meta-analysis to minimise participant heterogeneity among pooled analyses.

Where data for eligible participants were pooled with ineligible participants in an individual study, data were requested from the contact author via email for the eligible participants only. Where these data were provided by contact authors, they were re-analysed and included in this review and in relevant pooled analyses, and this was documented under ‘results’. Where no response was received, or data were not provided, the study was excluded and cited in the list of excluded studies (Additional file 1).

### Study Selection

Two reviewers (BP and MH) independently screened the titles and abstracts of all studies retrieved by the electronic search. Full texts were retrieved for any study that could not be excluded based on its title and abstract. Full-text articles were independently assessed by two reviewers (BP and SS), and disagreements were arbitrated by a third reviewer (VC). Study authors were contacted where necessary to determine eligibility and where additional data were required.

### Quality Appraisal

Risk of bias was assessed independently by two reviewers (BP and FH) based on a version of the procedure outlined by the 'Health Evidence Bulletins—Wales: Questions to assist with the critical appraisal of an observational study' tool [42], which was modified for this review. Modifications were made to the descriptive criteria of the tool to increase the specificity of those criteria to prospective studies of RRI incidence. Items which were of no relevance to this review were omitted, while relevant items were reframed so that their application within the current review was explicitly stated.

### Data Extraction and Analysis

Data were independently extracted by two authors (BP and SS) using standardised forms and cross-checked by BP. Review Manager 5.3 (The Cochrane Collaboration, Denmark) was used for all analyses. Meta-analysis was performed for each potential risk factor. Where there was adequate methodological homogeneity (similar sample sizes, participant characteristics, measurement methodologies, and duration of follow-up) and statistical homogeneity (*I*^2^ < 40%) fixed effects were used [43]. If not, random effects were used. If *I*^2^ was > 75%, indicating considerable statistical heterogeneity [43, 44], the studies were not pooled unless the source of the heterogeneity could be clearly explained [45]. Meta-analyses used weighted mean difference (WMD) unless there were differences in how the risk factor was measured, in which case standardised mean differences were used (SMD). Crude (unadjusted) estimates from individual studies were used in all analyses.

Nominal scaled data were dichotomised and used to calculate risk ratios (RR) with 95% confidence intervals (CI). Where data for the left and right side were reported, the right side only was included in the analysis to maintain independence of data [46, 47].

Magnitudes of standardised effects were interpreted following Hopkins’ modified version of Cohen’s scale, as used by Hume [48], as follows: < 0.20 = trivial; 0.20–0.59 = small; 0.60–1.19 = moderate; > 1.20 = large. A RR > 1.0 indicated that the risk of injury was higher in participants with the risk factor present. A small effect was indicated by a RR of ≥ 2.0, and a large effect by a RR ≥ 4.0 [46].

If a pooled analysis used WMD (expressed as the difference between groups and reported in the units of measure for the discrete risk factor) and a significant result was detected, both the WMD and the Cohen’s *d* effect size were reported to increase transparency of findings and to aid interpretation of results. In these cases, the Cohen’s *d* effect size was manually calculated [49] and interpreted in the same manner as per standardised effects as reported above.

## Results

### Study Identification

**Fig. 1:**
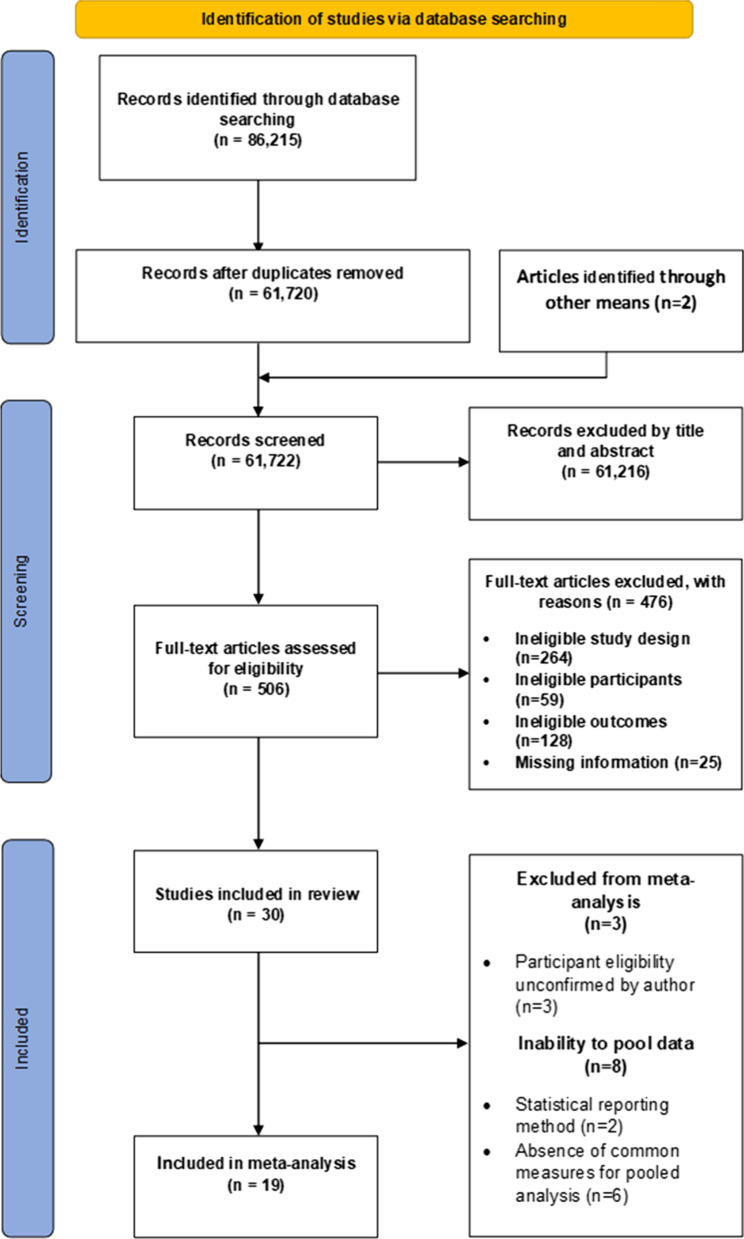
Prisma flow diagram

61,722 titles and abstracts (after removal of duplicates) were retrieved through electronic and manual searches and screened. Five-hundred-and-six potentially relevant full texts were retrieved and screened (Fig. 1). Thirty articles [4, 10, 14–29, 50–61] were included, while 476 were excluded based on the stated criteria (Additional file 1).

### Characteristics of Included Studies

Thirty studies, comprising 3404 runners (2267 female, 66.6%), investigated risk factors for RRI [4, 10, 15, 16, 18–22, 24, 25, 51, 53, 54, 56, 57, 59–61], exercise related lower leg pain [50], patellofemoral pain [17, 27, 29, 52], iliotibial band syndrome [26, 28, 58] and Achilles tendinopathy [14, 23]. Study duration ranged from 8 weeks [28] to 2 years [16]. A summary of included studies is presented in Table 1.

**Table 1:** Characteristics of included studies

Author (year)	Participant characteristics				Study design			
	Population	Sample size (completions)	% Female (*n*)	Mean age (SD)	Follow-up duration	Injury type	Injury incidence	Assessments conducted
Bennet et al. (2012) [50]	Competitive collegiate XC runners (unconfirmed) calibre)	77	43 (33)	19.3 (no SD)	1 XC season	ERLLP	26/59 (44.1%)	Navicular drop, plantarflexor endurance
Bring et al. (2018) [51]^a^	Collegiate XC runners (NCAA DIII)	81^a^	56 (45)	19.31 (1.12)	3 separate XC seasons	RRI	12/81 (14.8%)	Functional movement screen
Buist et al. (2010) [10]	Male and female novice runners	532	57.5 (306)	Female: 37.9 (9.9) Male: 42.3 (9.9)	13 weeks	RRI	100/532 (486 at risk) (18.8%)	Hip internal and external rotation ROM, ankle joint ROM (knee flexed and extended), navicular drop
Davis et al. (2003) [52]	18–45 yo female competitive distance runners of ≥ 20 mi./week (unconfirmed) calibre)	18^b^	100	Injured: 33.4 (8.2) Uninjured: 29.9 (11.3)	Unclear	PFPS	9/18 (matched) (% unknown)	Running kinematics
Davis and Mullineaux (2016) [24]	18–45 yo female recreational runners of ≥ 20 mi./week	249	100	Injured: 26.40 (9.2) Uninjured: 25.40 (9.2)	2 years	RRI	144/249 (all RRI) (57.8%) 103/249 (Dx Injuries) (44.1%)	Running kinetics
Desai and Gruber (2021) [59]	Recreational runners	39	61.5 (24)	Injured: 32.38 (11.68) Uninjured: 31.11 (9.62)	6 months	RRI	21/39 (53.8%)	Running kinematics, coordinative variability
Hamill et al. (2007) [58]	18–45 yo female recreational runners of ≥ 20 mi./week	34	100	Injured: 26.8 (8.04) Uninjured: 28.5 (12.1)	2 years	ITBS	17/400 (34 analysed)	Running kinetics
Hein et al. (2014) [14]	Recreational runners of ≥ 20 km/week	20^b^	20 (4)	Control: 40 (7) AT: 45 (5)	52 weeks	AT	10/142(AT) (20 analysed) (7%) 45/142 (RRI) (31.7%)	Strength measures: hip abduction and adduction, knee flexion and extension. Barefoot running kinematics
Hendricks and Phillips (2013) [19]^a^	Running club members	50^a^	32 (16)	46 (8.5)	16 weeks	RRI	16/50 (32%)	LLD, *Q*-angle, hip joint ROM (flexion, extension, abduction, adduction, internal and external rotation), knee flexion ROM, muscle strength (leg-press)
Hesar et al. (2009) [22]	New runners in a start-to-run program	131	85 (111)	39.09 (10.3)	10 weeks	RRI	27/131 (20.6%)	Running barefoot plantar pressure
Hespanhol et al. (2016) [20]	Recreational runners	89	23.6 (41)	44.2 (10.6)	12 weeks	RRI	24/89 (29.9%)	LLD, *Q*-angle, subtalar angle, plantar arch index
Hotta et al. (2015) [54]	18–24 year-old male competitive runners	84	0	20.0 (1.1)	6 months	RRI	15/84 (17.9%)	Functional movement screen
Jungmalm et al. (2020) [60]	Recreational runners	225	39.6 (89)	40.3 (8.1)	52 weeks	RRI	75/225 (33.3%)	Running kinematics, strength measures (hip abduction and adduction, knee flexion and extension, trunk flexion, extension, and rotation), strength ratios (hamstrings-quadriceps, trunk flexion–extension), joint ROM (hip flexion, extension, abduction, adduction, internal rotation and external rotation, knee flexion and extension, ankle dorsiflexion, plantarflexion, pronation, and supination), muscle flexibility (hamstrings, hip flexors), lower limb trigger points
Leetun et al. (2004) [15]^a^	Collegiate XC runners (non-NCAA)	10^a^	50 (5)	Injured: 33.4 (8.2) Uninjured: 29.9 (11.3)	1 XC season	RRI	2/10 (20%)	Strength: hip abduction and external rotation, back extension, and lateral core
Lun et al. (2004) [4]	Recreational runners	87	49 (43)	38.0 (no SD)	6 months	RRI	69/87 (79.3%)	ROM: hip internal and external rotation, ankle joint dorsi- and plantarflexion. *Q*-angle, STJ varus and valgus, forefoot varus and valgus, foot posture (subjective classification), genu varum, LLD
Messier et al. (2018) [16]	Recreational runners	300	42.6 (128)	Injured: 42.3 (9.7) Uninjured: 40.0 (10.3)	2 years	RRI	199/300 (66.3%)	Flexibility: hamstrings, quadriceps, ankle joint. *Q*-angle, arch index, muscle strength (hip, knee, ankle), running kinetics and kinematics
Napier et al. (2018) [25]	Healthy female recreational runners	65	100	Injured: 34.7 (7.8) Uninjured: 37.4 (8.2)	15 weeks	RRI	22/65 (33.8%)	Running kinetics and kinematics
Noehren et al. (2007) [26]	18–45 yo healthy female recreational runners of ≥ 20 mi./week	36^b^	100	Control: 26.8 (no SD) ITBS: 28.5 (no SD)	2 years	ITBS	18/400 (36 in analysis) (4.5%)	Running kinetics and kinematics
Noehren et al. (2013) [27]	Healthy female runners of ≥ 20 mi./week who heel-strike	30^b^	100	Control: 27 (10) PFPS: 27 (10)	2 years	PFPS	15/400 (30 in analysis) (3.8%)	Running kinematics
Peterson et al. (2020 unpbl) [53]	Adult recreational runners	59	51 (30)	48 (13.4)	6 months	RRI	30/59 (50.8%)	FPI, navicular drop, ankle joint lunge (knee flexed and extended)
Shen et al. (2019) [28]	18–25 yo male recreational runners	30	100	Injured: 20.40 (1.2) Uninjured: 19.70 (1.9)	8 weeks	ITBS	15/249 (30 in analysis) 6.0%	Running kinetics and kinematics
Stefanyshyn et al. (2006) [29]	20–50 yo runners of ≥ 20 km/week	80	48.7 (39)	Female: 35.9 (8) Male: 39.8 (8.9)	6 months	PFPS	6/80 (12 in analysis) (7.5%)	Running kinetics (knee abduction impulse)
Thijs et al. (2008) [55]	Novice recreational runners in a start-to-run program	102	87 (89)	37 (9.5)	10 weeks	PFPS	17/102 (16.7%)	Plantar pressure measurement and FPI
Thijs et al. (2011) [17]	Female novice recreational runners in a start-to-run program	77	100	38 (9)	10 weeks	PFPS	16/77 (20.8%)	*Q*-angle and hip muscle strength (flexion and extension, abduction and adduction, internal and external rotation)
Torp et al. (2018) [18]	Healthy female recreational runners	50	100	39.1 (9.4)	16 weeks	RRI	15/50 (30.0%)	Isometric strength: knee flexion and extension, hip flexion and extension, hip external rotation, hip abduction
Van Der Worp et al. (2016) [56]	Adult women training for 5/10 km event	435	100	38.7 (11.5)	12 weeks	RRI	93/417 (12 did not run) (22.3%)	Navicular drop, 1st metatarsophalangeal joint extension
Van Ginkel et al. (2009) [23]	Novice runners in a start-to-run program	63	84 (53)	Injured: 38 (11.35) Uninjured: 40 (9.0)	10 weeks	AT	10/63 (15.9%)	Plantar pressure measurement
Wen et al. (1998) [21]	Participants in a 32-week marathon training program	255	58 (143)	41.3 (10.8)	32 weeks	RRI	90/255 completions (35.3%)	Arch index, heel varus, tubercle-sulcus angle, knee varus, LLD
Winter et al. (2019) [61]	Recreational runners of different abilities (elite, advanced, intermediate, and slow)	76	39.4 (30)	Advanced injured 36.63 (10.13) Advanced non-injured 37.27 (10.92) Intermediate injured (47.07 (11.34) Intermediate non-injured 50.67 (10.96) Slow injured 36.46 (13.07) Slow non-injured 53.00 (7.23)	1 year	RRI	39/76 (51.3%)	Spatiotemporal parameters using body-mounted accelerometry
Zifchock (2007) [57]	18–45 yo non-injured runners of ≥ 20 mi./week	29	55 (16)	Injured: 27.9 (7.6) Uninjured: 31.1 (7.1)	9 months	RRI	14/29 (48.3%)	Running kinetics and kinematics, arch height index, *Q*-angle, hip abduction and external rotation strength, knee varus angle, hip internal rotation ROM

*AT* achilles tendinopathy, *DIII* division 3, *Dx* diagnosed, *ERLLP* exercise-related lower leg pain, *FPI* foot posture index, *ITBS* iliotibial band syndrome, *LLD* limb length discrepancy, *n* number, *NCAA* National collegiate athletics association, *PFPS* patellofemoral pain syndrome, *Q-angle* quadriceps angle, *ROM* range of motion, *RRI* running-related injury, *SD* standard deviation, *STJ* subtalar joint, *XC* cross-country

^a^Data reported were re-analysed to exclude participants not meeting the systematic review eligibility criteria

^b^n analysed in nested case-control

Five study authors provided additional data for inclusion in this review [15, 19, 25, 51, 60]. Two studies included some ineligible participants, i.e., below 18 years of age [51] or non-recreational runners [15]. Authors of both of those studies provided raw continuous data which have been re-analysed for use in this systematic review [15, 51]. One study author provided raw data for musculoskeletal assessment measures which were not reported in the original publication [19]. The author of one study which did not report continuous data in the original publication provided mean and SD data for relevant measures upon request [25]. Finally, the author of one study which did not report continuous data in the original publication provided raw data upon request [60].

### Methodological Quality

Methodological quality appraisal is presented in Table 2. Overall, included studies performed well on quality appraisal; however lack of control for confounding factors and missing information about dealing with multiple injuries were common limitations.

**Table 2:** Quality appraisal of included studies

	Bennett et al. (2012) [50]	Bring et al. (2017) [51]^a^	Buist et al. (2010) [10]	Davis et al. (2003) [52]	Davis and Mullineaux (2016) [24]	Desai and Gruber (2021) [59]	Hamill et al. (2007) [58]	Hein et al. (2014) [14]	Hendricks and Phillips (2013) [19]^a^	Hesar et al. (2009) [22]	Hespanhol et al. (2016) [20]	Jungmalm et al. (2020) [60]	Leetun et al. (2004) [15]^a^	Lun et al. (2004) [4]	Messier et al. (2018) [16]
Are the eligibility criteria appropriate for the aims of the study? e.g. were participants free from injury at baseline?	✓	✓	✓	?	✓	✓	✓	✓	✓	?	✓	✓	✓	✓	✓
Are the baseline assessment methods adequately described?	✓	✓	✓	✓	✓	✓	✓	✓	✓	✓	✓	✓	✓	✓	✓
Have the reliability and validity of baseline assessment methods been established?	✓	✓	*	✓	✓	✓	✓	*	*	✓	*	*	✓	*	*
Was the injury reporting method adequately described?	✓	✓	✓	X	X	X	X	✓	✓	X	Y	X	✓	✓	✓
For specific injury diagnoses, was there a suitably qualified assessor?^c^	X	n/a	n/a	?	n/a	n/a	?	?	n/a	✓	n/a	n/a	n/a	n/a	n/a
For specific injury diagnoses, was the assessor blinded to baseline results?^c^	X	n/a	n/a	?	n/a	n/a	?	?	n/a	?	n/a	n/a	n/a	n/a	n/a
For specific injury diagnoses, were all injuries diagnosed in the same manner?^c^	X	n/a	n/a	?	n/a	n/a	?	?	n/a	?	n/a	n/a	n/a	n/a	n/a
Did the authors state how they dealt with multiple injuries? e.g. only analysed first injury^d^	X	X	n/a	X	n/a	n/a	X	X	n/a	n/a	n/a	n/a	n/a	n/a	n/a
Were important confounders (e.g. training load) accounted for?	X	X	✓	X	✓	?	✓	✓	X	✓	X	X	✓	✓	✓
Is it likely that attrition rates and/or reasons affected the results of the study?	?	✓	n/a	n/a	?	X	n/a	X	?	n/a	n/a	?	✓	X	✓

	Napier et al. (2018) [25]	Noehren et al. (2007) [26]	Noehren et al. (2013) [27]	Peterson et al. (2020) [53]^b^	Shen et al. (2019) [28]	Stafanyshyn et al. (2006) [29]	Hotta et al. (2015) [54]	Thijs et al. (2008) [55]	Thijs et al. (2011) [17]	Torp et al. (2018) [17]	Van Der Worp et al. (2016) [35]	Van Ginckel et al. (2008) [23]	Wen et al. (1998) [21]	Winter et al. (2019) [61]	Zifchock (2007) [57]
Are the eligibility criteria appropriate for the aims of the study? e.g. were participants free from injury at baseline?	✓	✓	✓	✓	✓	✓	✓	✓	✓	✓	?	✓	✓	✓	?
Are the baseline assessment methods adequately described?	✓	✓	✓	✓	✓	✓	✓	✓	✓	✓	✓	✓	✓	✓	✓
Have the reliability and validity of baseline assessment methods been established?	✓	✓	✓	*	✓	✓	✓	✓	✓	*	✓	✓	?	?	*
Was the injury reporting method adequately described?	✓	X	X	✓	X	✓	X	X	X	✓	✓	✓	✓	X	X
For specific injury diagnoses, was there a suitably qualified assessor?^c^	n/a	✓	✓	n/a	?	✓	n/a	✓	✓	✓	n/a	?	n/a	n/a	?
For specific injury diagnoses, was the assessor blinded to baseline results?^c^	n/a	?	?	n/a	?	?	n/a	?	✓	✓	n/a	✓	n/a	n/a	?
For specific injury diagnoses, were all injuries diagnosed in the same manner?^c^	n/a	✓	✓	n/a	✓	✓	n/a	✓	✓	✓	n/a	✓	n/a	n/a	?
Did the authors state how they dealt with multiple injuries? e.g. only analysed first injury^d^	n/a	X	X	✓	X	X	n/a	X	X	n/a	n/a	n/a	n/a	n/a	n/a
Were important confounders (e.g. training load) accounted for?	✓	✓	✓	n/a	✓	✓	✓	✓	✓	✓	✓	✓	✓	?	X
Is it likely that attrition rates and/or reasons affected the results of the study?	✓	n/a	n/a	n/a	?	n/a	X	✓	✓	✓	?	✓	X	X	X

✓ = low risk; X = high risk; ? = can’t tell; *some but not all measures were known by the reviewers to be reliable

^a^Data for eligible participants provided and re-analysed in this review

^b^Some items incomplete as research is ongoing; additional information for some items provided by contact author

^c^n/a applied if the outcome was injured vs. not-injured (risk classification only applied to studies investigating risk factors for specific diagnoses)

^d^Only applied to studies interested in a specific injury diagnosis (n/a for studies comparing RRI vs. no RRI)

### Risk Factors for Running-Related Injury

The 30 studies investigated over 100 discrete biomechanical and musculoskeletal measurements as potential risk factors for RRI, of which there was commonality among 25 variables which were pooled in meta-analysis. Measurement techniques and results of individual studies are reported in Additional file 2.

### Meta-analysis

Nineteen studies were included in pooled analyses [4, 14–20, 24–28, 53, 55–57, 59, 60], while 11 studies were incompatible for pooled analysis, as described below. Two studies, by Buist et al. and Wen et al., were excluded from the meta-analysis as they reported only categorical data, which were not compatible with categorical data from any other study [10, 21], and the authors were not able to provide compatible data upon request. Three studies by Bennet et al. [50], Davis et al. [52], and Hotta et al. [54] were omitted from meta-analysis, and their results are reported descriptively as data on competition level could not be confirmed. The study by Bring et al. [51] reported data which were only compatible with the data from Hotta et al. [54] which had already been excluded from the analysis. Finally, five other studies were not included in meta-analysis as they each reported unique discrete risk factors which were not tested in other studies [22, 23, 29, 58, 61].

The findings of individual studies that were not included in the meta-analysis are reported narratively in the following sections, and are reported in Additional file 2.

**Fig. 2:**
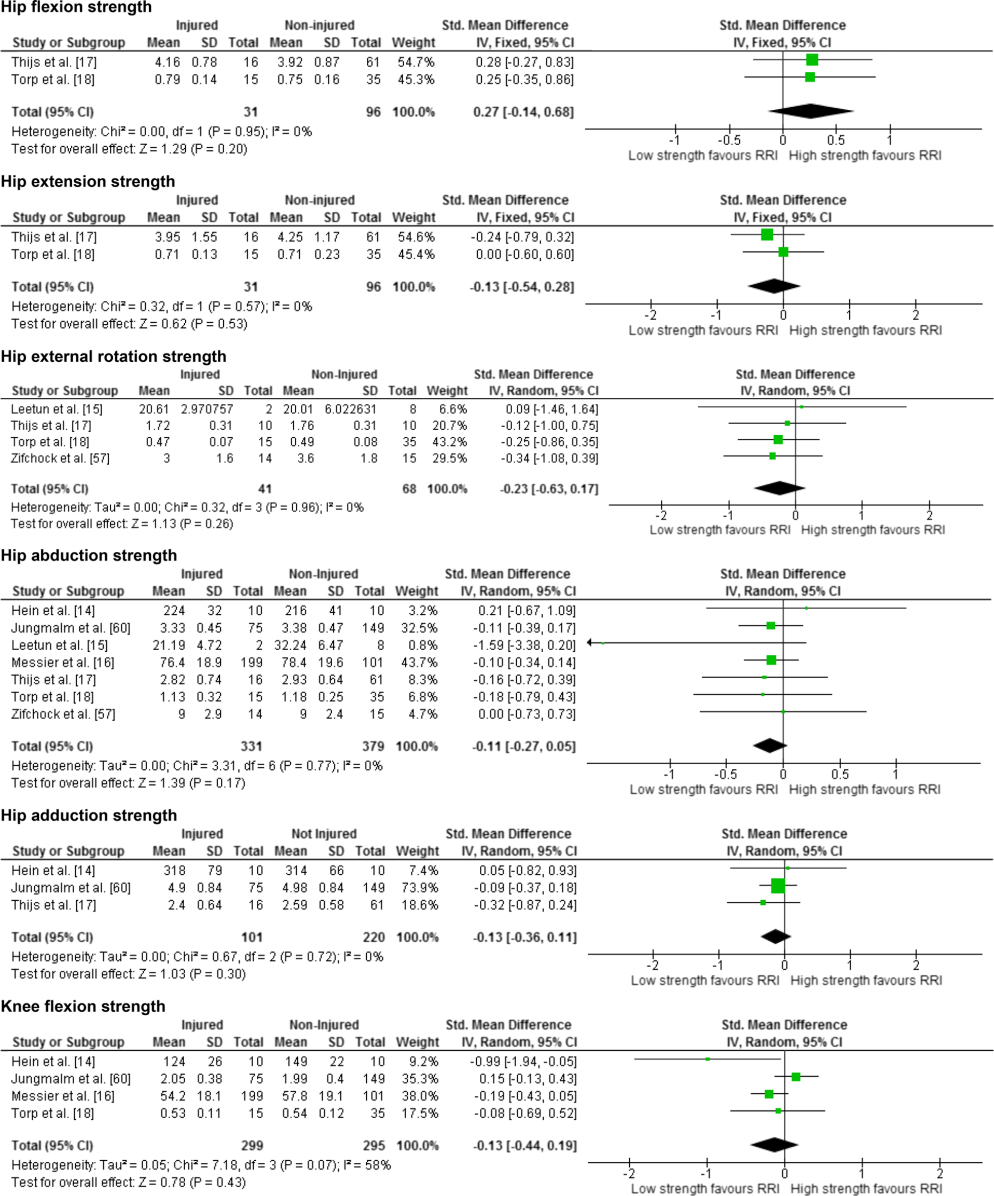

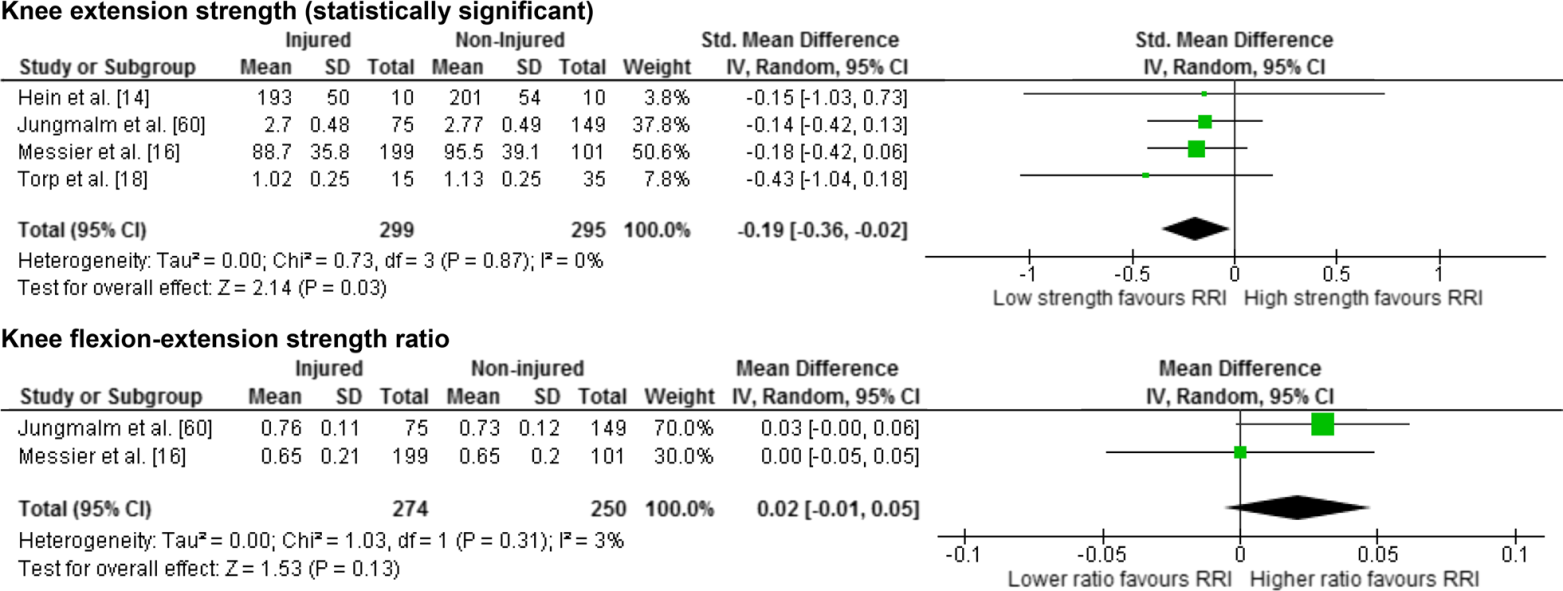
Forest plots for muscle strength measures as risk factors for RRI

**Fig. 3:**
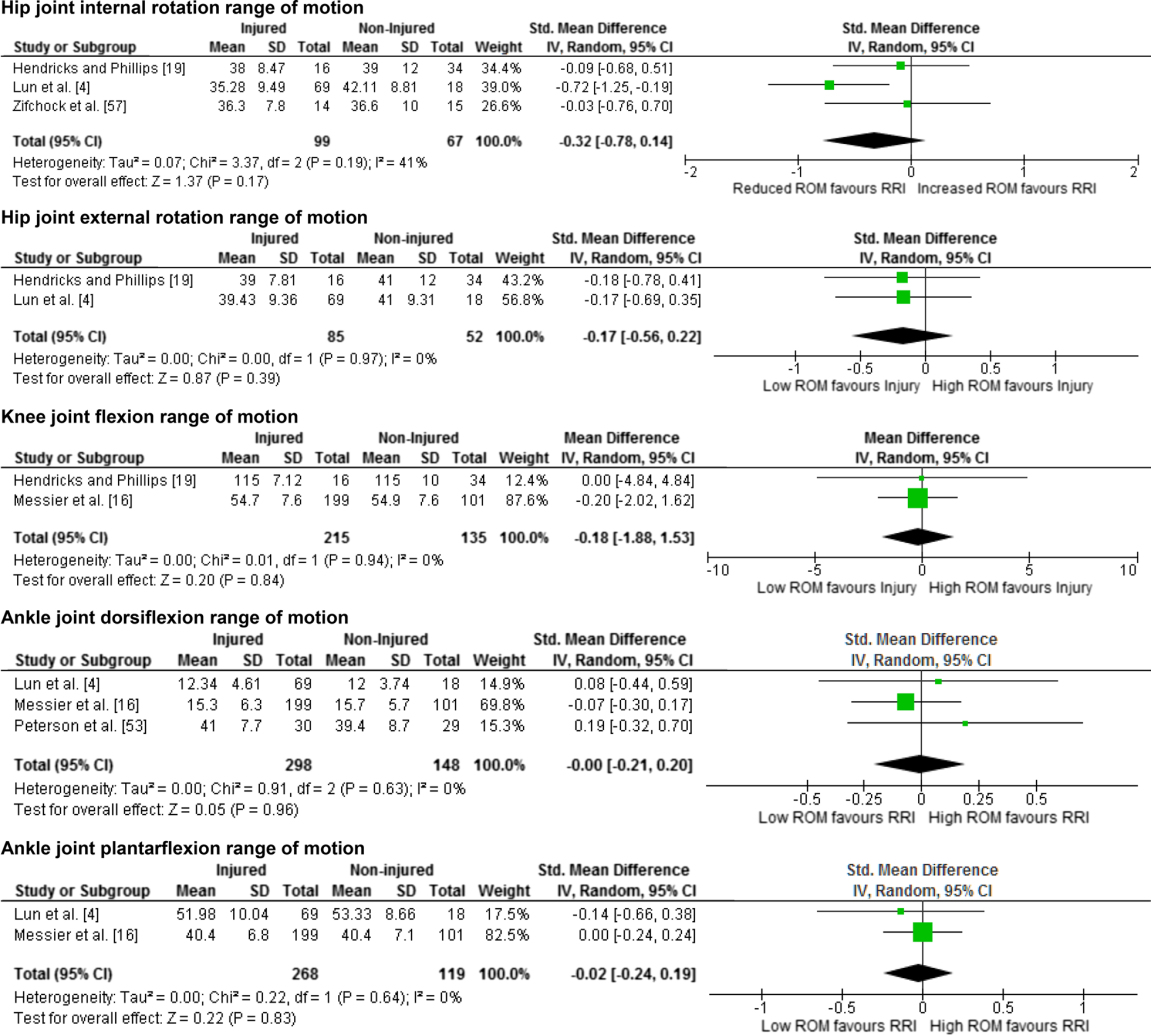
Forest plots for joint range of motion measures as risk factors for RRI

**Fig. 4:**
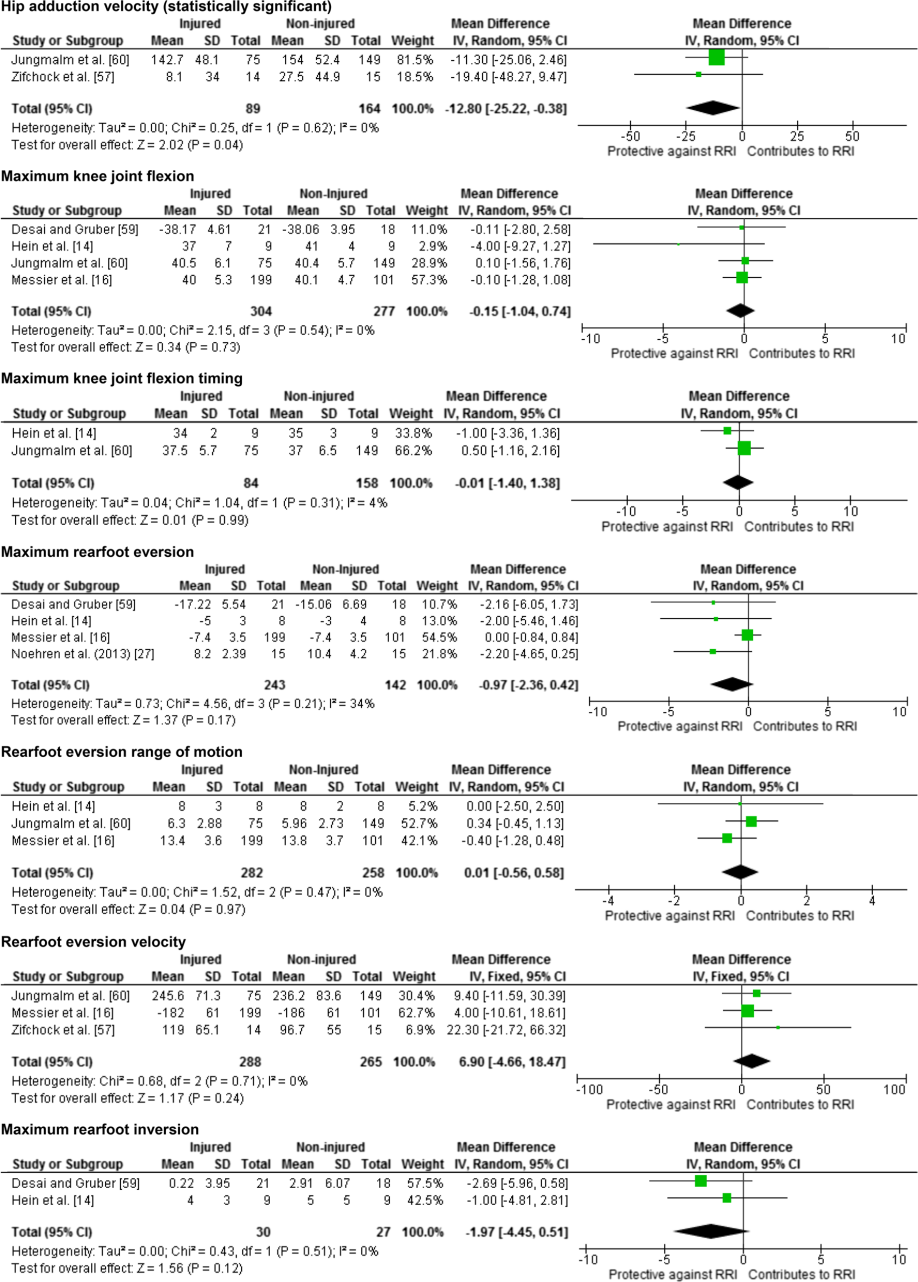
Forest plots for running kinematic measures as risk factors for RRI

**Fig. 5:**
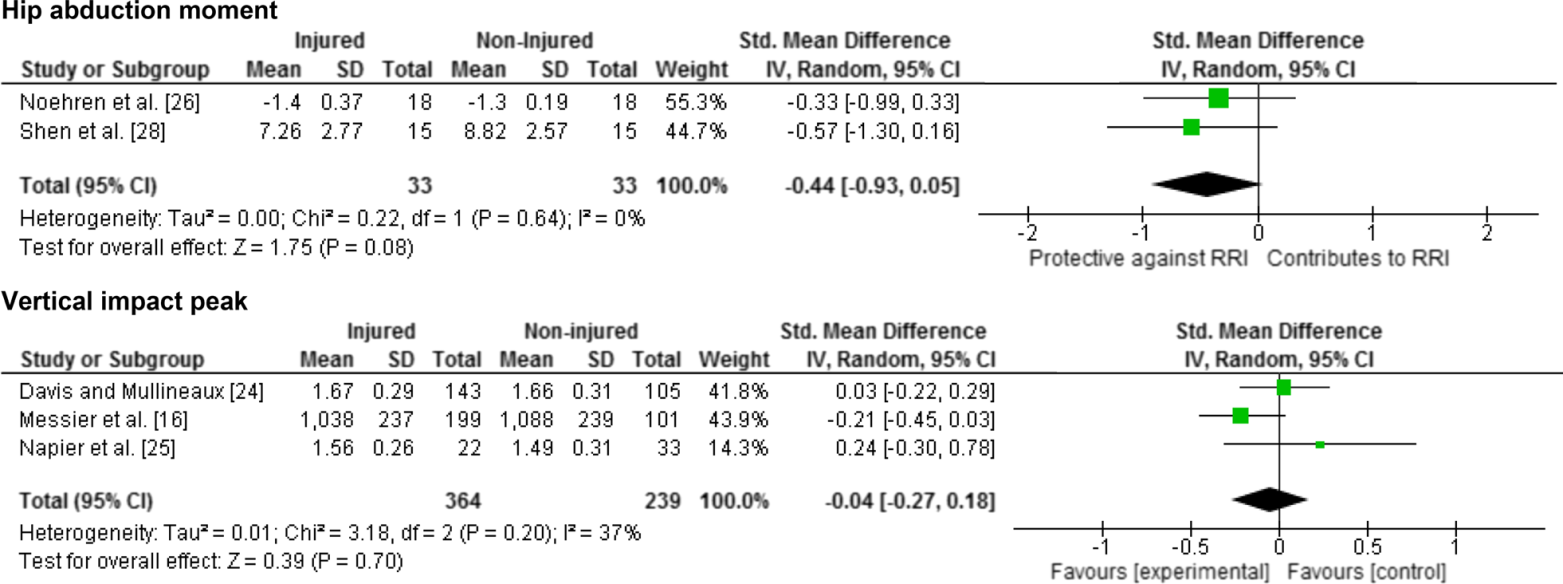
Forest plots for running kinetic measures as risk factors for RRI

**Fig. 6:**
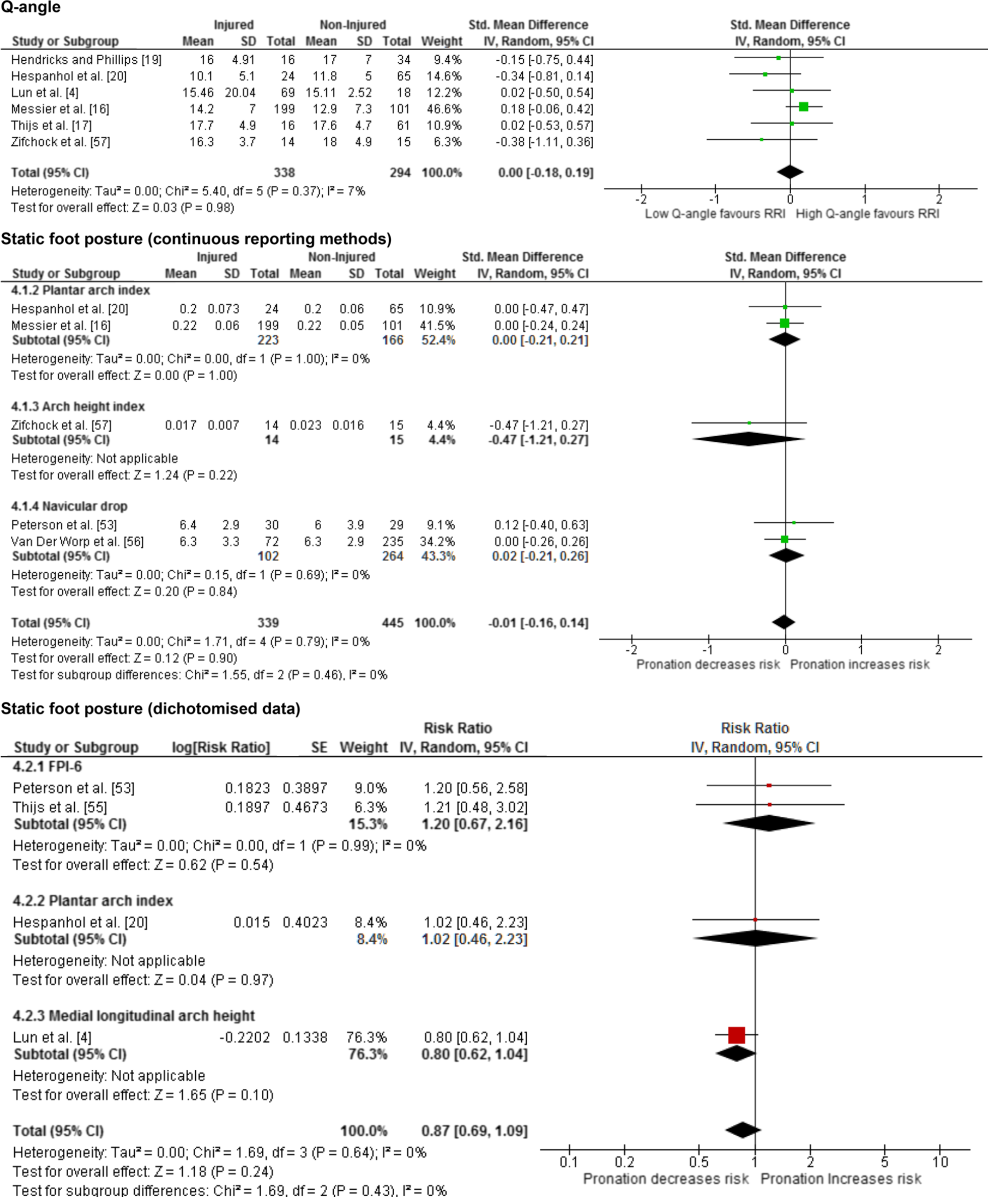
Forest plots for measures of alignment as risk factors for RRI

Twenty-five meta-analyses were performed to test the relationship between reported risk factors and RRI. Forest plots for pooled analyses were reported under five themes: muscle strength (Fig. 2), joint ROM (Fig. 3), running kinematics (Fig. 4), running kinetics (Fig. 5), and static lower limb alignment (Fig. 6). All pooled analyses are reported as SMD or WMD, with the exception of static foot posture where two separate meta-analyses were performed as pooled analysis was precluded by heterogeneity in statistical reporting methods. One of these meta-analyses included studies reporting continuous data and was analysed using SMD, and the other included studies reporting categorical data, which were dichotomised to calculate risk ratios.

Notably 23 of the 25 meta-analyses performed in this systematic review did not detect a significant difference between prospectively injured and non-injured runners for the included biomechanical or musculoskeletal variables (Figs. 2, 3, 4, 5, 6).

Two pooled analyses identified that results of baseline measures of knee extension strength and hip adduction velocity were significantly different between runners who did and did not prospectively develop a RRI. Four studies [14, 16, 18, 60] including 594 runners (299 injured, 271 female) reported eligible data for knee extension strength (Fig. 2). The analysis revealed significantly less knee extension strength among runners who prospectively developed a RRI, with a trivial effect size (SMD − 0.19, 95% CI − 0.36 to − 0.02, *p* = 0.03) and no evidence of statistical heterogeneity (*I*^2^ = 0%, *p* = 0.87) (Fig. 2). Standardised mean differences were calculated for the meta-analysis of this factor due to differences in measurement methods between studies (maximal voluntary isometric contraction [14, 18, 60] vs. maximal isokinetic contraction [16]). Significantly lower velocity of hip adduction during the stance phase of running was detected among runners who prospectively developed a RRI compared with those who did not (MD − 12.80 °/s, 95% CI − 25.22 to − 0.38, *p* = 0.04) with no evidence of statistical heterogeneity (*I*^2^ = 0%, *p* = 0.62) (Fig. 4). This analysis included two studies [57, 60] involving 253 runners (89 injured, 105 female) (Fig. 4) with WMD reported as the measurement methods were similar between the two studies. The effect size for this WMD was manually calculated and estimated to be small (0.32).

### Narrative Synthesis of Studies Omitted from Meta-analysis

There were several instances where a pooled analysis omitted the results of one or more relevant studies. The findings of these studies and the rationale for their omission are outlined below.

#### Joint Range of Motion

Two large studies by Buist et al. [10] and Jungmalm et al. [60], including 532 novice runners and 225 recreational runners respectively, each found no significant association between both passive hip joint internal rotation ROM or passive ankle joint dorsiflexion ROM and RRI, and were excluded from these meta-analyses due to not reporting or providing on request continuous data for these variables. Non-significant associations reported by Jungmalm et al. [60] for knee flexion ROM, hip external rotation ROM, and ankle joint plantarflexion ROM, and RRI, were unable to be pooled within relevant meta-analyses. After raw data were provided by these authors it was determined as these measures were recorded as ‘reference’, ‘hypermobile’, and ‘hypomobile’ at the time of data collection, and therefore could not be included.

#### Quadriceps-Angle

A conference abstract by Davis et al. [52] was omitted from the non-significant pooled analysis of the association between *Q*-angle and RRI incidence. In this abstract, Davis et al. [52] found a greater *Q*-angle in competitive runners who developed patellofemoral pain compared with controls (mean (SD) 16.1 (4.0) vs. 13.0 (3.1), *p* = 0.05). This study was excluded from meta-analysis as it could not be confirmed that the participants were not elite or sub-elite runners.

#### Static Measures of Foot Posture

Three studies were omitted from pooled analysis of the relationship between foot posture and RRI due to the absence of continuous data [10, 21] and unconfirmed participant eligibility [50]. Bennet et al. [50] included 77 (33 female) competitive collegiate cross-country runners and found runners with > 10 mm of navicular drop were 6.6 times more likely to sustain medial exercise related lower leg pain during a cross-country season (odds ratio (OR) 6.6, 95% CI 1.2 to 38.0, *p* = 0.03). This finding was supported by that of Buist et al. [10] who reported that female novice runners in a systematic training program were more likely to become injured with increasing amounts of navicular drop (hazard ratio (HR) 0.85, 95% CI 0.75 to 0.97). A study by Wen et al. [21] reported a higher arch index (ratio of height of the navicular tuberosity relative to medial longitudinal arch length) was protective against overall injuries and knee injuries.

#### Peak Rearfoot Eversion

Two studies by Noehren et al. reported data for peak rearfoot eversion as a risk factor for patellofemoral pain [27] and iliotibial band syndrome [26], respectively. Both studies reported non-significant findings. Both studies were nested case–control studies within a large prospective cohort study. It was not possible to determine if the data for the control groups of both studies were independent of one another and therefore these were not pooled for meta-analysis. For pooled analysis of peak rearfoot eversion, the level of statistical heterogeneity was tested when adding each of the studies by Noehren et al. to the analysis, one at a time. The study causing greater statistical heterogeneity [26] was omitted.

### Narrative Synthesis of Risk Factors Not Tested in Meta-analysis

Several risk factors were not tested by meta-analysis due to being reported in only one study or due to other factors precluding pooling of data. The findings of these risk factors are outlined below, with an explanation of why pooled analyses were not performed.

#### Joint Range of Motion

Hip joint flexion, extension, abduction and adduction ROM, and knee extension ROM were measured in two studies by Hendricks et al. [19] and Jungmalm et al. [60], each reporting non-significant results. These studies were not able to be pooled in meta-analysis due to Jungmalm et al. [60] not reporting continuous data for ROM measures.

#### Trigger Points

One study tested trigger points, defined as a tender area in a muscle that reproduces pain during palpation, in the iliotibial band, gastrocnemius, soleus, piriformis, gluteus medius, tibialis anterior, and tibialis posterior and detected no significant association with incidence of RRI [60]. The relationships between trigger points and RRI were tested in this one study only, preventing pooled analysis of this measurement.

#### Leg Length Discrepancy

Limb length discrepancy (LLD) was measured in four studies with similar methods [4, 19–21]. One study did not report the findings for this assessment in the published report [4] and did not provide data on request. Wen et al. [21] reported LLD was associated with more overall injuries (relative rate 1.96, 95% CI 1.07 to 3.58, *p* < 0.05) but did not report mean and SD for LLD measures, and was not able to provide these data for re-analysis, so was excluded from meta-analysis. Two studies that reported non-significant findings [19, 20] were eligible for meta-analysis, but significant statistical heterogeneity (*I*^2^ = 79%, *p* = 0.03) precluded their inclusion.

#### Muscle Strength

Ankle joint plantarflexion strength [16] and endurance [50] were assessed in two separate studies and were not significantly associated with RRI. Unconfirmed competition calibre in the study by Bennet et al. [50] precluded pooled analysis. Back extension strength was measured in three studies [14, 15, 60]. Hein et al. [14] did not report statistical comparisons between groups. Data provided by Leetun et al. [15] and Jungmalm et al. [60] were re-analysed for this review and no statistically significant associations with RRI were found. The studies by Leetun et al. [15] and Jungmalm et al. [60] were eligible for meta-analysis, but significant statistical heterogeneity precluded this (*I*^2^ = 81%, *p* < 0.01).

Jungmalm et al. [60] reported a higher rate of injury among recreational runners whose hip abduction to adduction strength ratio was > 1 standard deviation below the reference value (risk difference 17.3, 95% CI 0.8 to 33.7, *p* = 0.04). Other measures of muscle strength were reported in individual studies only, with results for individual studies reported in Additional file 2.

#### Functional Movement Screen

The relationship between functional movement screen (FMS) performance and RRI was investigated in two studies [51, 54]. Bring et al. [51] used the FMS in high-school and collegiate cross-country runners, with some runners below 18 years of age. Data for eligible participants were extracted and re-analysed for this review. Incidence and total number of RRI were not associated with any individual domain of the FMS; however there was a significantly greater number of injuries in those scoring below the suggested normative score of 14 out of 21 compared to those scoring 14 or greater (*p* = 0.045). A study by Hotta et al. [54] reported a higher incidence of RRI in competitive runners with a deep squat and active straight-leg raise combined score of ≤ 3 out of a total of six (OR 9.7, 95% CI 2.1 to 44.4, *p* < 0.01), but as there was uncertainty about the eligibility criteria for participants in this study, pooled analysis for FMS was not possible.

#### Running Gait Kinematics

Nine studies reported results of kinematic running gait assessment [14, 16, 26–28, 52, 59–61]. A study by Hein et al. [14] reported differences in sagittal plane kinematics between runners who did and did not develop Achilles tendinopathy, but did not provide statistical analyses to support this finding. Jungmalm et al. [60] reported a higher rate of RRI in runners whose rearfoot eversion timing was ≥ 1 standard deviation above the reference value (risk difference 20.7, 95% CI 1.3 to 40.0, *p* = 0.03) [60]. Desai et al. [59] reported significantly greater average ankle motion (− 11.34° (SD 5.9) vs. − 9.09° (SD 5.27), *p* < 0.01) and average shank motion (2.86° (SD 3.52) vs. 1.54° (SD 3.64), *p* < 0.01) among runners who developed a RRI over a 6-month follow-up. Noehren et al. [26] demonstrated significantly greater peak knee internal rotation in runners who developed iliotibial band syndrome versus matched controls (iliotibial band syndrome 3.9° (SD 3.7), controls 0.02° (SD 4.6), *p* = 0.01), whilst Davis et al. [52] reported a lesser degree of hip external rotation in runners who prospectively developed patellofemoral pain (5.1° SD (9.3) patellofemoral pain vs. 10.2° (SD 4.9) controls) with this latter analysis performed using a significance threshold of *p* = 0.10. These variables were only reported in individual studies so pooling of data was not possible.

Two studies by Noehren et al. demonstrated a greater peak hip adduction angle in female recreational runners who developed iliotibial band syndrome (14.1° (SD 12.5) vs. 10.6° (SD 5.1), (*p* = 0.01) [26] and between runners who developed patellofemoral pain compared with healthy controls (12.1° (SD 2.8) vs. 8.1° (SD 4.5), *p* = 0.007) [27]. Concern about the lack of independence of control groups precluded pooling of these data.

Desai et al. [59] measured coordinative variability (CAV) during the stance phase of running gait, and reported results against both the consensus definition of RRI [1] and a modified definition of RRI used by the authors of the study (i.e. any running related pain in the lower back or lower limbs that caused runners to stop or modify training for a minimum of 1 day). Significant associations were reported between the consensus definition of RRI [1] and CAV at initial contact, mid-stance, and late-stance. At initial contact, significant differences were reported between injured and non-injured runners for knee-ankle CAV (7.17 (SD 0.66) vs. 5.98 (SD 0.96), *p* < 0.05) and knee-shank CAV (7.22 (SD 1.2) vs. 5.59 (SD 1.34), *p* < 0.05). At mid-stance, knee-shank CAV was greater among injured runners (13.85 (SD 6.8) vs. 10.89 (SD 5.92), *p* < 0.05). During late-stance, shank-ankle CAV was greater among injured runners (5.48 (SD 2.61) vs. 4.77 (SD 3.48), *p* < 0.05). Desai et al. [56] also reported significant associations between the authors’ modified definition of RRI and CAV at initial contact and during late stance. At initial contact, significant differences were reported between injured and non-injured runners for knee-ankle CAV (7.03 (SD 0.61) vs. 6.05 (SD 1.16), *p* < 0.05) and knee-shank CAV (6.98 (SD 1.12) vs. 5.69 (SD 1.73), *p* < 0.05). During late stance, significant differences were reported between injured and non-injured runners for shank-ankle CAV (5.28 (SD 2.39) vs. 4.93 (SD 3.81), *p* < 0.05).

Other kinematic measures were reported in individual studies only and were not significantly associated with development of RRI (Additional file 2).

#### Running Gait Kinetics

Eight studies tested the relationship between RRI and running kinetic variables.[16, 24–26, 28, 29, 57, 58]. Five studies reported significant risk factors for RRI [16, 24, 25, 29, 58]. Napier et al. found peak braking force (PBF) to be a significant predictor of low-back or lower extremity RRI, reporting that runners in the highest PBF tertile (< − 0.27) become injured at 5.08 times those in the middle PBF (− 0.27 to 0.23) (HR 5.08, 95% CI 1.71 to 15.03, *p* = 0.003) tertile and 7.98 times those in the lowest tertile (> − 0.23) (HR 9.98, 95% CI 2.08 to 30.51, *p* = 0.002) [25]. Continuous data were provided by the contact author for Napier et al., which facilitated pooled analysis with brake force data reported by Messier et al. [16]. However, statistical heterogeneity precluded this (*I*^2^ = 92%, *p* < 0.001).

Messier et al. found increased knee joint stiffness to be a significant predictor of RRI, with every 6.89 Nm/deg increase in stiffness increasing the odds of RRI by 18% (OR 1.184, 1.021 to 1.374, *p* = 0.03) [16]. Knee stiffness was also reported by Zifchock et al. [57] who reported data for this variable as the deviation from the mean of a larger group of healthy runners; hence pooled analysis was not possible due to heterogeneity in statistical reporting.

Stefanyshyn et al. [29] prospectively compared runners sustaining patellofemoral pain to non-injured controls and found a significant increase in knee abduction impulse in injured runners (9.2 (SD 3.7) Nm/s vs. 4.7 (SD 3.5) Nm/s, *p* = 0.42). Hamill et al. [58] found iliotibial band strain rate to be greater during the support period (*p* = 0.001) in those who prospectively developed iliotibial band syndrome, and that this difference was most notable during mid-support. These kinetic variables were reported in only individual studies, precluding pooled analysis.

Davis et al. [24] reported no kinetic risk factors for prospectively injured compared to non-injured 18–45 year old female recreational runners, but demonstrated significant differences between those who reported never experiencing a RRI versus those who prospectively reported a medically diagnosed injury for vertical average load rate (VALR) (*p* = 0.001) and vertical instantaneous load rate (VILR) (*p* = 0.014). Both Davis et al. [24] and Napier et al. [25] reported data for VILR and VALR. Pooled analyses were precluded by significant statistical heterogeneity (VILR *I*^2^ = 77%, *p* = 0.04; VALR *I*^2^ = 76%, *p* = 0.04). Other kinetic factors were measured in individual studies and were not significant risk factors for RRI.

#### Spatiotemporal Parameters

One study by Winter et al. [61] reported differences in flight-time, step frequency, and step regularity, measured using a body-mounted accelerometer, in recreational runners who were identified as being ‘slow’ runners (runners who averaged less than 12 km/h running speed during an 8 km run at a self-selected speed). Flight-time measured in milliseconds (ms) among injured runners running at < 12 km/h, was significantly greater than in those who remained uninjured (117.27 (SD 15.44) v 93.39 (SD 19.55) ms, *p* < 0.05). Among the same group of runners, step frequency was lower among those who went on to develop a RRI (165.25 (SD 8.97) v 173.32 (SD 3.1), *p* < 0.05), as was step regularity (0.94 (SD 0.04) v 0.96 (SD 0.01), *p* < 0.05) measured using the vertical accelerometer axis. ‘Step regularity’ has been defined as the consistency of the step-to-step pattern [62].The same and additional spatiotemporal variables were measured in other groups sub-classified by running speed as ‘intermediate’ and ‘advanced’ with non-significant results (Additional file 2).

#### Plantar Pressures

Three studies investigated the relationship between plantar pressure measurements and incidence of RRI, reporting significant findings related to force distribution, and spatiotemporal measures [22, 23, 55]. The results of these studies were not pooled in meta-analysis due to measurement heterogeneity.

Van Ginkel et al. [23] reported a more laterally directed force distribution at forefoot flat (*p* = 0.016) and a decrease in total anterior–posterior displacement of the centre of force (COF) (*p* = 0.015) to be gait-related risk factors for Achilles tendinopathy. Thijs et al. [55] found increased vertical peak force at the lateral heel (*p* = 0.034) and a shorter time to peak force at the lateral heel (*p* = 0.048) in runners who developed patellofemoral pain. Peak vertical force was also higher at metatarsal 2 (*p* = 0.016) and metatarsal 3 (*p* = 0.026) in runners who prospectively developed patellofemoral pain.

Hesar et al. [22] reported participants who developed lower limb overuse injury had a significantly more laterally directed force distribution at first metatarsal contact and at forefoot flat, as well as a more laterally directed force displacement during forefoot contact, foot flat and heel off. In addition, injured runners demonstrated a delayed change in the COF at forefoot flat, higher force and loading underneath the lateral border of the foot, and a significantly higher directed force displacement of the COF at forefoot flat. These findings were based on interpretation of a large number of highly significant correlations which are reported in Additional file 2.

## Discussion

This is the first systematic review of non-elite adult runners to synthesise all available prospective evidence of biomechanical and musculoskeletal risk factors for RRI. Overall, results of the meta-analyses in this systematic review do not support the role of biomechanical and musculoskeletal measures as risk factors for the development of RRI in non-elite runners. Despite significant findings in several individual studies, twenty-three of twenty-five pooled analyses detected no relationship between baseline biomechanical and musculoskeletal measures and development of RRI. A finding of significantly less knee extension strength among prospectively injured runners compared with non-injured runners which was identified in pooled analysis in this review should be interpreted in the context of a trivial effect size. Likewise, the finding of statistically significantly lower hip adduction velocity among prospectively injured runners compared with non-injured runners should be interpreted very cautiously as this pooled analysis contained only two studies which both individually reported no association between this measure and RRI, and in light of one of those studies being small and of uncertain methodological quality [57].

The results of the present study expand on a previous review by Christopher et al. [38] which reported mixed results for muscle strength, flexibility, ROM, and alignment for predicting injury in recreational runners, but did not include meta-analyses with review findings limited by very low quality of evidence for each assessment and the variability in measurement and reporting in the included studies [38]. In the present review, several of the risk factors from Christopher et al. [38] were tested in meta-analysis but produced non-significant findings. In the present review, the trivial effect of knee extension strength on the incidence of RRI justifies further investigation of this measure as a risk factor for RRI.

The results of the present review also expand on those of Ceyssens et al. [37] who performed a systematic review of biomechanical risk factors for RRI using a narrative synthesis, justifying the omission of meta-analyses based on between-study heterogeneity in participants and biomechanical variables. In the current review, meta-analysis detected no significant relationship between kinematic or kinetic measures and incidence of RRI, with the previously noted exception of hip adduction velocity. Another risk factor, hip abduction moment, approached statistical significance for incidence of iliotibial band syndrome, but was limited by the fact that this only involved two studies, both with small sample sizes [26, 28]. Studies with larger samples investigating these factors are required to confirm the relevance of these risk factors.

In the current review, the two studies by Noehren et al. [26, 27] were not included together in pooled analysis as the independence of the control groups in these studies could not be confirmed. Our conservative approach differs from a recent systematic review by Vannatta et al. [39], which pooled the data of both studies by Noehren et al. [26, 27], and subsequently reported peak hip adduction angle and peak rearfoot eversion as the only significant risk factors for RRI in pooled analysis [39]. Also, Vannatta et al. [39] performed pooled analysis for vertical instantaneous loading rate and vertical average loading rate, which was not performed in the present review due to statistical heterogeneity. Therefore, until additional evidence is available to undertake more robust analyses, these data need to be interpreted with caution.

Static measures of foot posture were not significantly associated with RRI in meta-analysis in the present review. This finding is consistent with the findings of a systematic review by Neal et al. [46] which found no relationship between foot posture and foot and ankle injury or general lower limb injury in a pooled analysis. Neal et al. [46] reported significant associations between navicular drop, resting calcaneal position, and foot posture index (FPI) and medial tibial stress syndrome, in pooled analysis, and a significant association between navicular drop and patellofemoral pain, but only in individual studies. The difference in the result of the pooled analysis of static measures of foot posture in the present study, when compared with those reported by Neal et al. [46], is possibly due to the difference in the outcome (RRI vs. medial tibial stress syndrome) but also the notable differences in the participant population between the reviews (adult non-elite runners vs. a sample of adult, high-school, recreational, and competitive runners) resulted in inclusion of several studies in their meta-analyses that were excluded from the current review. It should also be noted that despite reaching statistical significance, the association between navicular drop and patellofemoral pain reported in the review by Neal et al. [46] was based on a mean difference of 0.9 mm difference between injured and non-injured groups [63], which is likely not clinically meaningful.

An important consideration when interpreting the findings of meta-analysis of foot posture as a risk factor for RRI in the current review is the exclusion of three studies [10, 21, 50] due to incompatible data, which may have affected the results of this analysis. Future studies of biomechanical and musculoskeletal risk factors for RRI should report measurement mean and standard deviation data for injured and non-injured groups, as well as their selected statistical reporting methods, since heterogeneous statistical reporting methods continue to be a limiting factor when attempting to synthesise the existing literature in this area.

## Limitations

Missing information, heterogeneity in assessment and statistical reporting methods, and unexplained statistical heterogeneity (*I*^2^ > 75%) precluded meta-analysis of a number of biomechanical and musculoskeletal variables and excluded potentially important studies from relevant meta-analyses. There was some heterogeneity among definitions and diagnoses of RRI used by studies included in this review, which may have influenced the results of individual pooled analyses, The absence of a consensus definition for ‘elite’ and ‘sub-elite’ distance runners is a limiting factor. The authors cautiously approached this by excluding participants described as ‘competitive’ from all meta-analyses unless the contact author from the relevant study confirmed their non-elite status. This resulted in three studies of ‘competitive runners’ [50, 52, 54] being excluded from the pooled analysis, and their omission should be considered when interpreting the results of this systematic review. It is also possible, due to missing information within studies which could not be verified by contact authors, that those same three studies [50, 52, 54] reported narratively in this review included participants competing at levels higher than those eligible for this review, which should be considered when interpreting the results of the narrative synthesis in this review.

There are important methodological differences between included studies which are important to consider. This systematic review included five prospective studies [14, 26–29] which used case–control approaches to their statistical analysis in which injured runners were compared against an equal number of matched non-injured runners, which may have introduced the potential for selection bias within those studies. Differences in injury surveillance methods are also important to note, such as when comparing the results of a study which surveyed participants weekly for the incidence of RRI using an online form [16] against a study where specific types of RRI were self-reported at the end of an athletic season [50], or where participants reported only injuries which had met specific diagnostic criteria such as patellofemoral pain [27]. Between-study differences in assessment procedures are also important to consider when interpreting the results of this review, e.g. measurement of running kinematics in overground vs. treadmill conditions, and muscle strength testing using isokinetic vs. hand-held dynamometry. The potential for measurement error within individual studies should also be considered.

While this review included a broad scope of literature, and synthesised the relationship between RRI and numerous measures, the tight eligibility criteria for ‘non-elite runners’ limited the capacity for pooling of data. Although every effort was made to undertake a complete and robust review, it is possible that important studies were missed by our search strategy. The notion of clinical significance was not discussed for the results of analyses of discrete risk factors due to differences in assessment methods between studies preventing the use of weighted mean difference analyses in most cases. Finally, there is an inherent possibility of publication bias [64], that is, that additional studies recording null-findings have been performed and subsequently have not been published. While every attempt has been made to account for this, including comprehensive screening of a trove of grey literature, this remains a possible limitation of this systematic review.

## Directions for Future Research

Recent literature has highlighted the importance of correct interpretation of non-causal relationships between biomechanical and musculoskeletal characteristics and RRI [65]. It has been suggested that atypical biomechanical function alone does not cause RRI but may interact with training characteristics as an effect measure modifier to contribute to the risk of RRI [66]. A recent causal framework has suggested that running-related injury occurs when a runner possesses multiple risk factors, and then participates in running under particular circumstances to a degree which exceeds a structure's load capacity [67]. This is given some credence by the lack of significant findings reported in this review, in which biomechanical and musculoskeletal parameters alone were considered, independent of participation-related variables. There is some evidence that particular training characteristics, especially relating to the rate of progression in training volume and intensity, may contribute to the risk of RRI [11]. While the contribution of training parameters to RRI remains not well understood [12], this may support the suggestion that the relationship between training characteristics and biomechanical variables should be considered in determining RRI causality [66]. Research into such relationships is justified. Measures reaching or approaching statistical significance in the present review, including knee extension strength, hip adduction velocity, and hip abduction moment, may warrant further investigation in adequately powered prospective studies.

## Recommendations for Clinicians

There is currently insufficient evidence for any musculoskeletal or biomechanical assessments as risk factors for RRI to recommend their use for preventative screening, clinically. The authors reiterate the caution which should be applied when interpreting the results of the two significant pooled analyses in this review, and their clinical implications should not be overstated.

## Conclusion

This is the first systematic review of non-elite adult runners to synthesise all currently available data for biomechanical and musculoskeletal risk factors for RRI. Despite significant results in individual studies, meta-analyses of the currently available literature found no meaningful association between biomechanical or musculoskeletal factors and RRI in non-elite runners. Consequently, injury prevention strategies for these runners cannot be made on the basis of biomechanical and musculoskeletal measurements alone.

Relationships between biomechanical and musculoskeletal factors and RRI should be further explored in future studies, and these studies should report continuous data alongside reporting of other statistical measures.

### Supplementary Information

**Additional file 1.** Table of excluded studies.

**Additional file 2.** Risk factors for RRI – Results from individual studies.

## Data Availability

The datasets used for analysis in this review can be provided by the corresponding author on reasonable request.

## Appendix 1: Ovid MEDLINE Search Strategy

**Table:**

1	Jogg*
2	Runn*
3	1 OR 2
4	Risk
5	Prospective
6	Injur*
7	Predict
8	Associat*
9	Relation*
10	4 OR 5 OR 6 OR 7 OR 8 OR 9
11	3 AND 10
12	Limit 11 to human

* indicates truncation of search terms
