# Supplementary material for: Biomechanical and Musculoskeletal Measurements as Risk Factors for Running-Related Injury in Non-elite Runners: A Systematic Review and Meta-analysis of Prospective Studies
Source: Sports Med Open. 2022 Mar 7;8:38. doi: 10.1186/s40798-022-00416-z (PMC8901814; doi:10.1186/s40798-022-00416-z)
Supplement: Supplementary file 1 — Additional file 1. Table of excluded studies. [file 40798_2022_416_MOESM1_ESM.docx]

| **Citation** | **Justification for exclusion** |
| --- | --- |
| Clinical update. Running the risk of injury. Journal of Musculoskeletal Medicine. 2003;20(11):523-6. | Ineligible study design |
| In Search of Midfoot. Running & FitNews. 2012;30(1):8-10. | Ineligible study design |
| 2019 AMSSM Abstracts. Clinical Journal of Sport Medicine Conference: Annual Meeting of the American Medical Society for Sports Medicine, AMSSM 2019;29(2) | Ineligible study design |
| 2020 AMSSM Abstracts. Clinical Journal of Sport Medicine Conference: 29th Annual Meeting American Medical Society for Sports Medicine, AMSSM 2020;30(2) | Ineligible study design |
| Baltich J, Emery CA, Whittaker JL, Nigg BM. Running injuries in novice runners enrolled in different training interventions: a pilot randomized controlled trial. Co-Kinetic Journal. 2016(70):6-. | Ineligible study design |
| Abate M, Oliva F, Schiavone C, Salini V. Achilles tendinopathy in amateur runners: role of adiposity (Tendinopathies and obesity). Muscles, Ligaments & Tendons Journal (MLTJ). 2012;2(1):44-8. | Ineligible study design |
| Alexander JLN, Barton CJ, Willy RW. Infographic running myth: static stretching reduces injury risk in runners. British journal of sports medicine 2019;06 | Ineligible study design |
| Allmon AL, Cronk NJ, Morris L. Does recreational running increase the risk of OA of the knee? Evidence-Based Practice. 2015;18(2):11-2. | Ineligible study design |
| Altman AR, Davis IS. Prospective comparison of running injuries between shod and barefoot runners. BJSM online. 2016;50(8):476-80. | Ineligible outcomes |
| Anderson GS. Iliotibial band friction syndrome. Australian Journal of Science and Medicine in Sport. 1991;23(3):81-3. | Ineligible study design |
| Aranda Bolívar Y, Munuera PV, Polo Padillo J. Relationship Between Tightness of the Posterior Muscles of the Lower Limb and Plantar Fasciitis. Foot Ankle Int. 2013;34(1):42-8. | Ineligible study design |
| Aiyegbusi AI, Tella BA, Sanusi GA. Is genu varum a risk factor for The prevalence and severity of Achilles tendinopathy? A crosssectional Study of nigerian elite Track and field athletes. Nigerian Postgraduate Medical Journal 2020;27(2):87–92 | Ineligible study design |
| Bach DK, Green DS, Jensen GM, Savinar E. A comparison of muscular tightness in runners and nonrunners and the relation of muscular tightness to low back pain in runners. Journal of Orthopaedic and Sports Physical Therapy. 1985;6(6):315-23. | Ineligible outcomes |
| Bailey M, Dew M, Moore A. A comparative analysis of the incidence of overuse knee injuries in triathletes, runners and cyclists. British Journal of Therapy & Rehabilitation. 1996;3(10):537-41. | Ineligible study design |
| Baker RL, Souza RB, Fredericson M. Iliotibial band syndrome: soft tissue and biomechanical factors in evaluation and treatment. Pm R. 2011;3(6):550-61. | Ineligible study design |
| Baltich J, Emery CA, Stefanyshyn D, Nigg BM. The effects of isolated ankle strengthening and functional balance training on strength, running mechanics, postural control and injury prevention in novice runners: design of a randomized controlled trial. BMC Musculoskelet Disord. 2014;15:407. | Ineligible study design |
| Bandy WD, Timm KE. Relationship between peak torque, work, and power for knee flexion and extension in clients with grade 1 medial compartment sprains of the knee. J Orthop Sports Phys Ther. 1992;16(6):288-92. | Ineligible study design |
| Bartholomeeusen K, Meeusen R, Cumps E. Is the novice runner at risk? A prospective cohort study of running injuries during a 10-week supervised training program. BJSM online. 2008;42(6):505-. | Ineligible outcomes |
| Baur H, Hirschmuller A, Grau S, Horstmann T, Dickhuth H, Mayer F. Muscular endurance of the lower leg muscles in runners with unilateral achilles tendon complaints. Isokinetics and Exercise Science. 2002;10(1):62-3. | Ineligible study design |
| Begizew DM, Grace JM, Van Heerden HJ. Lower-extremity running-related injuries among 10,000-meter long distance runners in Ethiopia. Journal of Human Sport & Exercise. 2019;14(2):358-73. | Ineligible outcomes |
| Benca E, Listabarth S, Flock FKJ, et al. Analysis of running-related injuries: The Vienna study. Journal of Clinical Medicine 2020;9(2) | Ineligible study design |
| Bennell KL, Malcolm SA, Thomas SA, Reid SJ, Brukner PD, Ebeling PR, et al. Risk factors for stress fractures in track and field athletes: A twelve- month prospective study. Am J Sports Med. 1996;24(6):810-8. | Ineligible participant |
| Bennell KL, Malcolm SA, Thomas SA, Wark JD, Brukner PD. The incidence and distribution of stress fractures in competitive track and field athletes. A twelve-month prospective study. Am J Sports Med. 1996;24(2):211-7. | Ineligible participant |
| Bennett J, Reinking M, Pluemer B, Pentel A, Seaton M, Killian C. Factors contributing to the development of medical tibial stress syndrome in high school runners. Journal of Orthopaedic and Sports Physical Therapy. 2001;31(9):504-10. | Ineligible participants |
| Blair SN, Kohl HW, Goodyear NN. Rates and risks for running and exercise injuries: Studies in three populations. Research Quarterly for Exercise and Sport. 1987;58(3):221-8. | Ineligible outcomes |
| Bovens AM, Janssen GM, Vermeer HG, Hoeberigs JH, Janssen MP, Verstappen FT. Occurrence of running injuries in adults following a supervised training program. Int J Sports Med. 1989;10 Suppl 3:S186-90. | Ineligible outcomes |
| Bramah C, Preece SJ, Gill N, et al. Running kinematics differ between male and female runners with ITBS. International Journal of Sports Physical Therapy 2019;14(6):S2-S2. | Ineligible study design |
| Brauer M. Faktoren fur die Entwicklung von Beschwerden bei deutschen Marathonlaufern: Ergehnisse einer Befragung von Athleten vor dem 38. Berlin-Marathon - explorative Pilotstudie. Zeitschrift fur Physiotherapeuten Krankengymnastik. 2013;65(5):18-25. | Ineligible outcomes |
| Bredeweg S, Buist I. No relationship between running related injuries and kinetic variables. BJSM online. 2011;45(4):328-. | Missing information |
| Bredeweg S, Kluitenberg B, Bessem B, Buist I. Differences in kinetic variables between injured and noninjured novice runners: A prospective cohort study. Journal of Science and Medicine in Sport. 2013;16(3):205-10. | Ineligible study design |
| Bredeweg SW, Buist I, Kluitenberg B. Differences in kinetic asymmetry between injured and noninjured novice runners: a prospective cohort study. Gait Posture. 2013;38(4):847-52. | Ineligible study design |
| Brill PA, Macera CA. The influence of running patterns on running injuries. Sports Med. 1995;20(6):365-8. | Ineligible study design |
| Brukner P, Bennell K. Stress fractures in runners. Journal of Back and Musculoskeletal Rehabilitation. 1995;5(4):341-51. | Ineligible study design |
| Brumitt J. Injury prevention for high school female cross-country athletes. Athletic Therapy Today. 2009;14(4):8-12. | Ineligible study design |
| Brund R, Rasmussen S, Nielsen R, Kersting U, Laessoe U, Voigt M. Medial shoe-ground pressure and specific running injuries: A 1-year prospective cohort study. Journal of Science and Medicine in Sport. 2017;20(9):830-4. | Ineligible study design. |
| Brund RBK, Rasmussen S, Kersting UG, Arendt-Nielsen L, Palsson TS. Prediction of running-induced Achilles tendinopathy with pain sensitivity - a 1-year prospective study. Scand J Pain. 2019;19(1):139-46. | Ineligible study design |
| Brunet ME, Cook SD, Brinker MR, Dickinson JA. A survey of running injuries in 1505 competitive and recreational runners. J Sports Med Phys Fitness. 1990;30(3):307-15. | Ineligible outcomes |
| Buist I, Bredeweg S. Incidence and risk factors of running-related injuries in female recreational runners. BJSM online. 2008;42(6):506-. | Missing information |
| Buist I, Bredeweg SW, Bessem B, van Mechelen W, Lemmink KA, Diercks RL. Incidence and risk factors of running-related injuries during preparation for a 4-mile recreational running event. BJSM online. 2010;44(8):598-604. | Ineligible outcomes |
| Buist I, Bredeweg SW, Lemmink KA, Pepping GJ, Zwerver J, van Mechelen W, et al. The GRONORUN study: is a graded training program for novice runners effective in preventing running related injuries? Design of a Randomized Controlled Trial. BMC Musculoskelet Disord. 2007;8:24. | Ineligible study design |
| Busseuil C, Freychat P, Guedj E, Lacour J. Rearfoot-forefoot orientation and traumatic risk for runners. Foot and Ankle International. 1998;19(1):32-7. | Ineligible study design |
| Cabak A, Cichocki P. Assessment of the most common injuries of the musculoskeletal system in amateur long-distance runners. Polish Journal of Sports Medicine / Medycyna Sportowa 2020;36(2):101-06. | Ineligible study design |
| Cavanagh PR. The biomechanics of lower extremity action in distance running. Foot Ankle. 1987;7(4):197-217. | Ineligible study design |
| Chan ZYS, Zhang JH, Au IPH, An WW, Shum GLK, Ng GYF, et al. Gait Retraining for the Reduction of Injury Occurrence in Novice Distance Runners: 1-Year Follow-up of a Randomized Controlled Trial. Am J Sports Med. 2018;46(2):388-95. | Missing information |
| Chorley JN, Cianca JC, Divine JG, Hew TD. Baseline injury risk factors for runners starting a marathon training program. Clin J Sport Med. 2002;12(1):18-23. | Ineligible outcomes |
| Chow T, Chen Y, Wang J. Characteristics of plantar pressures and related pain profiles in elite sprinters and recreational runners. Journal - American Podiatric Medical Association. 2018;108(1):33-44. | Ineligible study design |
| Clement DB, Taunton JE. A guide to the prevention of running injuries. Aust Fam Physician. 1981;10(3):156-61, 63-4. | Ineligible study design |
| Cloosterman KLA, Fokkema T, de Vos RJ, et al. Consequences and Prognosis of Running-Related Knee Injuries Among Recreational Runners. Clinical journal of sport medicine: official journal of the Canadian Academy of Sport Medicine 2020;15 | Ineligible outcomes |
| Colbert LH, Hootman JM, Macera CA. Physical activity-related injuries in walkers and runners in the aerobics center longitudinal study Clin J Sport Med. 2000;10(4):259-63. | Ineligible outcomes |
| Crevier LM. Biomechanics report. Gait retraining may prevent knee problems. Journal of Musculoskeletal Medicine. 2009;26(6):232-3. | Ineligible study design |
| Cushman DM, Petrin Z, Eby S, et al. Ultrasound evaluation of the patellar tendon and Achilles tendon and its association with future pain in distance runners. Physician and Sportsmedicine 2020 | Ineligible outcomes |
| Dahle LK, Mueller M, Delitto A, Diamond JE. Visual assessment of foot type and relationship of foot type to lower extremity injury. J Orthop Sports Phys Ther. 1991;14(2):70-4. | Ineligible participants |
| Daly C, McCarthy Persson U, Twycross-Lewis R, Woledge RC, Morrissey D. The biomechanics of running in athletes with previous hamstring injury: A case-control study. Scand J Med Sci Sports. 2016;26(4):413-20. | Ineligible study design |
| Damsted C, Parner ET, Sorensen H, Malisoux L, Hulme A, Nielsen RO. The Association Between Changes in Weekly Running Distance and Running-Related Injury: Preparing for a Half Marathon. The Journal of orthopaedic and sports physical therapy. 2019;49(4):230-8. | Ineligible outcomes |
| Damsted C, Parner ET, Sorensen H, Malisoux L, Nielsen RO. ProjectRun21: Do running experience and running pace influence the risk of running injury-A 14-week prospective cohort study. Journal of Science & Medicine in Sport. 2019;22(3):281-7. | Ineligible outcomes |
| Dane S, Can S, Gürsoy R, Ezirmik N. Sport injuries: relations to sex, sport, injured body region. Percept Mot Skills. 2004;98(2):519-24. | Ineligible participant |
| Dane S, Can S, Karsan O. Relations of body mass index, body fat and power of various muscles to sport injuries. Percept Mot Skills. 2002;95(1):329. | Ineligible participant |
| Davis I. The re-emergence of the minimal running shoe. Journal of Orthopaedic and Sports Physical Therapy. 2014;44(10):775-84. | Ineligible study design |
| Davis IS. Gait retraining in runners. Orthopaedic Physical Therapy Practice. 2005;17(2):8-13. | Ineligible study design |
| Davis IS, Futrell E. Gait Retraining: Altering the Fingerprint of Gait. Phys Med Rehabil Clin N Am. 2016;27(1):339-55. | Ineligible study design |
| Davis Iv JJ, Gruber AH. Injured runners do not replace lost running time with other physical activity. Med Sci Sports Exerc 2020;52(5):1163-68 | Ineligible outcomes |
| Dekker T, Metzl J, Fontana MA, et al. Risk factors for injuries in marathon runners: A 16-week prospective study of the NYC marathon. Clin J Sport Med 2020;30 (2):184-85. | Ineligible outcomes |
| Devereaux MD, Lachmann SM. Athletes attending a sports injury clinic--a review. BJSM online. 1983;17(4):137-42. | Ineligible study design |
| Dicharry J. Kinematics and kinetics of gait: from lab to clinic. Clin Sports Med. 2010;29(3):347-64. | Ineligible study design |
| Dod F, Cascioli V. The importance of training intensity and foot type with regard to injury rates in club and recreational runners. European Journal of Chiropractic. 2003;51(3):167-72. | Ineligible study design |
| Duckham RL, Brooke-Wavell K, Summers GD, Cameron N, Peirce N. Stress fracture injury in female endurance athletes in the United Kingdom: A 12-month prospective study. Scand J Med Sci Sports. 2015;25(6):854-9. | Ineligible participants |
| Dudley RI, Pamukoff DN, Lynn SK, Kersey RD, Noffal GJ. A prospective comparison of lower extremity kinematics and kinetics between injured and non-injured collegiate cross country runners. Hum Mov Sci. 2017;52:197-202. | Ineligible participants |
| Dutto DJ, Braun WA. DOMS-associated changes in ankle and knee joint dynamics during running. Med Sci Sports Exerc. 2004;36(4):560-6. | Ineligible participants |
| Ellis J. Shoe types and construction: their relation to running injuries. Topics in Acute Care & Trauma Rehabilitation. 1986;1(2):28-37. | Ineligible study design |
| Eskofier BM, Kraus M, Worobets JT, Stefanyshyn DJ, Nigg BM. Pattern classification of kinematic and kinetic running data to distinguish gender, shod/barefoot and injury groups with feature ranking. Comput Methods Biomech Biomed Engin. 2012;15(5):467-74. | Missing information |
| Fanciullo JJ, Bell CL. Stress fractures of the sacrum and lower extremity. Curr Opin Rheumatol. 1996;8(2):158-62. | Ineligible study design |
| Favero T, VanderWilde A. Reducing Stress Fractures in Female College Distance Runners. Training & Conditioning 2020;30(4):10-13. | Ineligible study design |
| Fields KB, Delaney M, Hinkle JS. A prospective study of type A behavior and running injuries. Journal of Family Practice. 1990;30(4):425-9. | Ineligible outcomes |
| Finnoff JT, Hall MM, Kyle K, Krause DA, Lai J, Smith J. Hip strength and knee pain in high school runners: a prospective study. Pm R. 2011;3(9):792-801. | Ineligible participants |
| Fitch KD. Stress fractures of the lower limbs in runners. Aust Fam Physician. 1984;13(7):511-5. | Ineligible study design |
| Foch E, Milner C. Lower extremity joint position sense in runners with and without a history of knee overuse injury. Gait and Posture. 2012;36(3):557-60. | Ineligible study design |
| Fokkema T, Burggraaff R, Hartgens F, Kluitenberg B, Verhagen E, Backx FJG, et al. Prognosis and prognostic factors of running-related injuries in novice runners: A prospective cohort study. Journal of Science and Medicine in Sport. 2018. | Ineligible outcomes |
| Fokkema T, Hartgens F, Kluitenberg B, Verhagen E, Backx FJG, van der Worp H, et al. Reasons and predictors of discontinuation of running after a running program for novice runners. Journal of Science and Medicine in Sport. 2018. | Ineligible outcomes |
| Fokkema T, de Vos RJ, van Ochten JM, Verhaar JAN, Davis IS, Bindels PJE, et al. Online multifactorial prevention programme has no effect on the number of running-related injuries: a randomised controlled trial. British journal of sports medicine. 2019;06. | Ineligible outcomes |
| Fredericson M, Cookingham CL, Chaudhari AM, Dowdell BC, Oestreicher N, Sahrmann SA. Hip abductor weakness in distance runners with iliotibial band syndrome. Clin J Sport Med. 2000;10(3):169-75. | Ineligible study design |
| Fredericson M, Misra AK. Epidemiology and aetiology of marathon running injuries. Sports Med. 2007;37(4-5):437-9. | Ineligible study design |
| Fredericson M, Tenforde AS. Running Injuries. Phys Med Rehabil Clin N Am. 2016;27(1):xv-xvi. | Ineligible study design |
| Fries JF, Singh G, Morfeld D, O'Driscoll P, Hubert H. Relationship of running to musculoskeletal pain with age. A six-year longitudinal study. Arthritis Rheum. 1996;39(1):64-72. | Ineligible outcomes |
| Fuller J. The longer-term effects of minimalist running shoes on lower limb structure and function, running performance, and injury risk. Journal of Science and Medicine in Sport. 2017;20(Supplement 3):5-6. | Ineligible study design |
| Fuller JT, Thewlis D, Buckley JD, Brown NA, Hamill J, Tsiros MD. Body Mass and Weekly Training Distance Influence the Pain and Injuries Experienced by Runners Using Minimalist Shoes: A Randomized Controlled Trial. The American journal of sports medicine. 2017;45(5):1162-70. | Ineligible study design |
| Fuller JT, Thewlis D, Tsiros MD, Brown NA, Buckley JD. The long-term effect of minimalist shoes on running performance and injury: design of a randomised controlled trial. BMJ Open. 2015;5(8):e008307. | Ineligible study design |
| Fuller JT, Thewlis D, Tsiros MD, et al. Longer-term effects of minimalist shoes on running performance, strength and bone density: A 20-week follow-up study. European Journal of Sport Science 2019;19(3):402-12 | Ineligible study design |
| Fulmer DC. Achilles tendon disorders in runners. Med Sci Sports Exerc. 1981;13(1):x-xi. | Ineligible study design |
| Gaku T, Rieko K, Yuki M, Shota E, Yuki K, Takuya S. The Risk Factors of Hamstring Strain Injury Induced by High-Speed Running. Journal of Sports Science & Medicine. 2018;17(4):650-5. | Ineligible participants |
| Gallas JE. Risk Factors for Low Back Pain in Recreational Distance Runners.. 2017:1-. | Ineligible study design |
| Geiringer S. The biomechanics of running. Journal of Back and Musculoskeletal Rehabilitation. 1995;5(4):273-9. | Ineligible study design |
| Gerlach K, White S, Burton H, Dorn J, Leddy J, Horvath P. Kinetic changes with fatigue and relationship to injury in female runners. Medicine and Science in Sports and Exercise. 2005;37(4):657-63. | Ineligible participants |
| Goldman JT, Miller E, Runestad S, et al. Should Adolescents Run Marathons?: Youth Marathon Training Injury Epidemiology and Risk Factors. Clinical journal of sport medicine : official journal of the Canadian Academy of Sport Medicine 2020;15 | Ineligible outcomes |
| Gómez-Álvarez N, Mora Jiménez E, Astorga Cáceres B, Contreras Quintanilla N, Cancino Vásquez I, Pavez-Adasme G. Equilibrio dinámico y calidad del movimiento en corredores aficionados. / Dynamic balance and quality of the movement in amateur runner. Revista Ciencias de la Actividad Física UCM. 2019;20(1):105-15. | Ineligible study design |
| Gonzalez-Lazaro J, Arribas-Cubero HF, Rodriguez-Marroyo JA. Musculoskeletal injuries in mountain running races: A 5 seasons study. Injury 2020 | Ineligible outcomes |
| Grau S, Krauss I, Maiwald C, Best R, Horstmann T. Hip abductor weakness is not the cause for iliotibial band syndrome. Int J Sports Med. 2008;29(7):579-83. | Ineligible study design |
| Gremion G. Barefoot running: Is that different in the implications for running injuries? Schweizerische Zeitshrift fur Sportmedizin und Sporttraumatologie. 2014;62(1):5. | Ineligible study design |
| Grimshaw PN, Sinclair MJ. Biomechanical assessment of running gait and running-induced injuries. J Sports Sci. 1991;9(4):397-8. | Ineligible study design |
| Gudas CJ. Patterns of lower-extremity injury in 224 runners. Compr Ther. 1980;6(9):50-9. | Ineligible study design |
| Hamill J. What have we learned about barefoot, minimalist and forefoot running? Journal of Science and Medicine in Sport. 2015;19:e88. | Ineligible study design |
| Hamill J. Have modern footwear reduced running-related injury risk? Journal of Science and Medicine in Sport. 2015;19:e70. | Ineligible study design |
| Hanna C. Lower extremity injuries among high school cross-country runners: Commentary. Clin J Sport Med. 2007;17(2):171. | Ineligible study design |
| Harris M, Ryan MB, Taunton JE. Footwear usage and injury patterns in fitness class participants: A prospective pilot study. Clin J Sport Med. 2011;21 (4):383. | Ineligible outcomes |
| Hart LE. Exercise and soft tissue injury. Baillieres Clin Rheumatol. 1994;8(1):137-48. | Ineligible study design |
| Hart LE. Prevention of running-related injuries among novices. Clin J Sport Med. 2009;19(1):77-9. | Ineligible study design |
| Hayes LE, Boulos A, Cruz AI. Risk factors for in-season injury in varsity collegiate cross-country athletes: an analysis of one season in 97 athletes. The Journal of sports medicine and physical fitness. 2019;59(9):1536-43. | Ineligible outcomes |
| Hashish R, Samarawickrame SD, Sigward S, Azen SP, Salem GJ. Lower-limb dynamics and clinical outcomes for habitually shod runners who transition to barefoot running. Phys Ther Sport. 2018;29:93-100. | Ineligible study design. |
| Heil B. Lower limb biomechanics related to running injuries. Physiotherapy. 1992;78(6):400-6. | Ineligible study design |
| Hendricks C, Phillips J. Factors associated with road-running injuries. Physiotherapy (United Kingdom). 2011;97:eS473-eS4. | Ineligible study design |
| Hespanhol Junior LC, Mechelen W, Postuma E, Verhagen E. Health and economic burden of running-related injuries in runners training for an event: A prospective cohort study. Scand J Med Sci Sports. 2016;26(9):1091-9. | Ineligible outcomes |
| Hespanhol Junior LC, Pena Costa LO, Lopes AD. Previous injuries and some training characteristics predict running-related injuries in recreational runners: a prospective cohort study. J Physiother. 2013;59(4):263-9. | Ineligible outcomes |
| Hjerrild M, Videbaek S, Theisen D, Malisoux L, Oestergaard Nielsen R. How (not) to interpret a non-causal association in sports injury science. Phys Ther Sport. 2018;32:121-5. | Ineligible study design |
| Ho K-Y, Baquet A, Chang Y-J, et al. Factors related to intra-tendinous morphology of Achilles tendon in runners. PLoS ONE 2019;14(8):e0221183. | Ineligible study design |
| Hofstede H, Franke T, van ER, et al. In training for a marathon: Runners and running-related injury prevention. Phys Ther Sport 2020;41:80-86. | Ineligible outcomes |
| Hollander K. Biomechanics of Running -- Implications for Running-Related Injuries and Future Areas for Research. German Journal of Sports Medicine / Deutsche Zeitschrift fur Sportmedizin 2020;71(3):53-54. | Ineligible study design |
| Hollander K, Johnson CD, Outerleys J, et al. Multifactorial Determinants of Running Injury Locations in 550 Injured Recreational Runners. Medicine and science in sports and exercise 2020;04 | Ineligible study design |
| Hollander K, Liebl D, Meining S, Mattes K, Willwacher S, Zech A. Adaptation of Running Biomechanics to Repeated Barefoot Running: A Randomized Controlled Study. The American journal of sports medicine. 2019;47(8):1975-83. | Ineligible outcomes |
| Hoo JS, Krabak BJ, Kasmer M, Vandeleur D, Ciol MA. Injury in ultramarathon runners and its association with foot strike pattern and gait characteristics. PM and R. 2015;1):S203-S4. | Ineligible study design |
| Hootman JM, Macera CA, Ainsworth BE, Martin M, Addy CL, Blair SN. Predictors of lower extremity injury among recreationally active adults. Clin J Sport Med. 2002;12(2):99-106. | Ineligible outcomes |
| Hreljac A, Marshall R, Hume P. Evaluation of lower extremity overuse injury potential in runners. Medicine and Science in Sports and Exercise. 2000;32(9):1635-41. | Ineligible study design |
| Hsu CL, Yang CH, Wang JH, et al. Common running musculoskeletal injuries and associated factors among recreational gorge marathon runners: An investigation from 2013 to 2018 taroko gorge marathons. International Journal of Environmental Research and Public Health 2020;17(21):1-13. | Ineligible outcomes |
| Hubbard TJ, Carpenter EM, Cordova ML. Contributing factors to medial tibial stress syndrome: a prospective investigation. Med Sci Sports Exerc. 2009;41(3):490-6. | Ineligible participants |
| Hulkko A, Alen M, Orava S. Stress fracture of the lower leg. Scandinavian Journal of Sports Sciences. 1987;9(1):1-8. | Ineligible study design |
| Hulkko A, Orava S. Stress fractures in athletes. Int J Sports Med. 1987;8(3):221-6. | Ineligible study design |
| Humble RN, Haverstock BD. Running injuries of the lower extremity: preface. Clin Podiatr Med Surg. 2001;18(2):1 pg- pg. | Ineligible study design |
| Jacobs S, Berson B. Injuries to runners: a study of entrants to a 10,000 meter race. Am J Sports Med. 1986;14(2):151-5. | Ineligible study design |
| Jakobsen BW, Kroner K, Schmidt SA, Jensen J. Running injuries sustained in a marathon race. Registration of the occurrence and types of injuries in the 1986 Arhus Marathon. [Danish]. Ugeskr Laeger. 1989;151(35):2189-92. | Ineligible study design |
| Jakobsen BW, Kroner K, Schmidt SA, Kjeldsen A. Prevention of injuries in long-distance runners. Knee Surg Sports Traumatol Arthrosc. 1994;2(4):245-9. | Ineligible outcomes |
| Jandacka D, Uchytil J, Zahradnik D, et al. Running and physical activity in an air-polluted environment: The biomechanical and musculoskeletal protocol for a prospective cohort study 4HAIE (healthy aging in industrial environment-program 4). International Journal of Environmental Research and Public Health 2020;17(23):1-20 | Ineligible outcomes |
| Jandackova VK, Elavsky S, Uchytil J, et al. Physical Activity, Air Pollution and Health: The Neurocognitive and Brain Imaging Protocol in the 4haie Study. Psychosomatic Medicine 2020;82 (6):A52-A53 | Ineligible outcomes |
| Jauhiainen S, Pohl A, Ayramo S, et al. A hierarchical cluster analysis to determine whether injured runners exhibit similar kinematic gait patterns. Scandinavian Journal of Medicine and Science in Sports 2020;30(4):732-40. | Ineligible study design |
| Jensen SL, Andresen BK, Mencke S, Nielsen PT. Epidemiology of ankle fractures. A prospective population-based study of 212 cases in Aalborg, Denmark. Acta Orthop Scand. 1998;69(1):48-50. | Ineligible participants |
| Johnson AW, Myrer JW, Mitchell UH, Hunter I, Ridge ST. The Effects of a Transition to Minimalist Shoe Running on Intrinsic Foot Muscle Size. Int J Sports Med. 2016;37(2):154‐8. | Ineligible outcomes |
| Johnston R, Cahalan R, Bonnett L, Maguire M, Nevill A, Glasgow P, et al. Training Load and Baseline Characteristics Associated With New Injury/Pain Within an Endurance Sporting Population: A Prospective Study. International journal of sports physiology & performance. 2019;14(5):590-7. | Ineligible outcomes |
| Jorgensen U. Body load in heel-strike running: the effect of a firm heel counter. Am J Sports Med. 1990;18(2):177-81. | Ineligible study design |
| Kalin VX, Denoth J, Stacoff A, Stussi E. Running injuries and running shoe construction: demonstration of possible correlations. [German]. Sportverletzung Sportschaden : Organ der Gesellschaft fur Orthopadisch-Traumatologische Sportmedizin. 1988;2(2):80-5. | Ineligible study design |
| Kelsey JL, Bachrach LK, Procter-Gray E, Nieves J, Greendale GA, Sowers M, et al. Risk factors for stress fracture among young female cross-country runners. Med Sci Sports Exerc. 2007;39(9):1457-63. | Ineligible outcomes |
| Kemler E, Blokland D, Backx F, Huisstede B. Differences in injury risk and characteristics of injuries between novice and experienced runners over a 4-year period. The Physician and sportsmedicine. 2018;46(4):485-91. | Ineligible outcomes |
| Kiernan D, Hawkins DA, Manoukian MAC, McKallip M, Oelsner L, Caskey CF, et al. Accelerometer-based prediction of running injury in National Collegiate Athletic Association track athletes. J Biomech. 2018;73:201-9. | Missing information |
| Kluitenberg B, Middelkoop M, Smits DW, Verhagen E, Hartgens F, Diercks R, et al. The NLstart2run study: Incidence and risk factors of running-related injuries in novice runners. Scand J Med Sci Sports. 2015;25(5):e515-e23. | Ineligible outcomes |
| Kluitenberg B, van der Worp H, Huisstede BMA, Hartgens F, Diercks R, Verhagen E, et al. The NLstart2run study: Training-related factors associated with running-related injuries in novice runners. Journal of Science and Medicine in Sport. 2016;19(8):642-6. | Ineligible outcomes |
| Kluitenberg B, van Middelkoop M, Verhagen E, Hartgens F, Huisstede B, Diercks R, et al. The impact of injury definition on injury surveillance in novice runners. Journal of Science and Medicine in Sport. 2016;19(6):470-5. | Ineligible outcomes |
| Knobloch K, Yoon U, Vogt P. Acute and overuse injuries correlated to hours of training in master running athletes. Foot and Ankle International. 2008;29(7):671-6. | Ineligible participants |
| Koenig SJ, Toth AP, Bosco JA. Stress fractures and stress reactions of the diaphyseal femur in collegiate athletes: an analysis of 25 cases. Am J Orthop. 2008;37(9):476-80. | Ineligible outcomes |
| Koplan J, Rothenberg R, Jones E. The natural history of exercise: a 10-yr follow-up of a cohort of runners. Medicine and Science in Sports and Exercise. 1995;27(8):1180-4. | Ineligible outcomes |
| Koplan JP, Powell KE, Sikes RK, Shirley RW, Campbell CC. An epidemiologic study of the benefits and risks of running. Jama. 1982;248(23):3118-21. | Ineligible outcomes |
| Kornaat PR, Van de Velde SK. Bone marrow edema lesions in the professional runner. Am J Sports Med. 2014;42(5):1242-6. | Ineligible study design |
| Kraus E, Nattiv A, Deakins-Roche M, et al. Low total T3 associated with lower lumbar spine BMD and low energy availability in male and female collegiate distance runner. Clin J Sport Med 2020;30 (2):108-09. | Ineligible outcomes |
| Kretsch A, Grogan R, Duras P. 1980 Melbourne marathon study. Med J Aust. 1984;141(12-13):809-14. | Ineligible outcomes |
| Krivickas LS, Feinberg JH. Lower extremity injuries in college athletes: relation between ligamentous laxity and lower extremity muscle tightness. Arch Phys Med Rehabil. 1996;77(11):1139-43. | Ineligible participants |
| Kuhman DJ, Paquette MR, Peel SA, Melcher DA. Comparison of ankle kinematics and ground reaction forces between prospectively injured and uninjured collegiate cross country runners. Hum Mov Sci. 2016;47:9-15. | Ineligible participants |
| Lagas IF, Fokkema T, Bierma-Zeinstra SMA, et al. How many runners with new-onset Achilles tendinopathy develop persisting symptoms? A large prospective cohort study. Scand J Med Sci Sports 2020;30(10):1939-48. | Ineligible outcomes |
| Lagas IF, Fokkema T, Verhaar JAN, et al. Incidence of Achilles tendinopathy and associated risk factors in recreational runners: A large prospective cohort study. Journal of Science and Medicine in Sport 2020;23(5):448-52. | Ineligible outcomes |
| Lane GD, Malhotra S, Davis Iv JJ. The Podiatrist's Role in Treating Running Injuries in Today's Society. Podiatry Management. 2013;32(2):83-6. | Ineligible study design |
| Larkins PA. Evaluating runners' injuries. Aust Fam Physician. 1984;13(7):503-6. | Ineligible study design |
| Lemberg D. Overuse injuries: shin splints. Chiropractic Journal. 1988;2(6):13-22. | Ineligible study design |
| Leon-Guereno P, Tapia-Serrano MA, Sanchez-Miguel PA. The relationship of recreational runners' motivation and resilience levels to the incidence of injury: A mediation model. PLoS ONE 2020;15(5):e0231628. | Ineligible study design |
| Letafatkar A, Rabiei P, Farivar N, Alamouti G. Long-Term Efficacy of Conditioning Training Program Combined with Feedback on Kinetics and Kinematics in Male Runners. Scandinavian journal of medicine & science in sports. 2019;30. | Ineligible study design |
| Leumann A, Pagenstert G, Frigg A, Ebneter L, Hintermann B, Valderrabano V. Foot and lower leg stress fractures in sports. [German]. Fuss und Sprunggelenk. 2006;4(3):150-7. | Ineligible study design |
| Levine D, Prall E, Hanks J, Whittle M, Marcellin-Little D. Running and the development of osteoarthritis, Part II: Human studies. Athletic Therapy Today. 2003;8(1):12-8. | Ineligible study design |
| Little RMD, Paterson DH, Humphreys DA, Stathokostas L. A 12-month incidence of exercise-related injuries in previously sedentary community-dwelling older adults following an exercise intervention. BMJ Open. 2013;3 (6) (no pagination)(e002831). | Ineligible participants |
| Liu L, Gisselman AS, Tumilty S. Thermal profiles over the Patella tendon in a cohort of non-injured collegiate athletes over the course of a cross-country season. Phys Ther Sport 2020;44:47-52. | Ineligible outcomes |
| Longo UG, Berton A, Stelitano G, et al. 2017 Marathon of Rome: Anthropometry and Sport Profile in 350 Runners and Association With Achilles and Patellar Tendinopathy. Clinical journal of sport medicine: official journal of the Canadian Academy of Sport Medicine 2021;31(1):e15-e20. | Ineligible study design |
| Lubetzky-Vilnai A, Carmeli E, Katz-Leurer M. Prevalence of injuries among young adults in sport centers: relation to the type and pattern of activity. Scand J Med Sci Sports. 2009;19(6):828-33. | Ineligible study design |
| Luedke LE, Heiderscheit BC, Williams DSB, Rauh MJ. Association of isometric strength of hip and knee muscles with injury risk in high school cross country runners. International Journal of Sports Physical Therapy. 2015;10(6):868-76. | Ineligible participants |
| Luedke LE, Heiderscheit BC, Williams DSB, Rauh MJ. Influence of Step Rate on Shin Injury and Anterior Knee Pain in High School Runners. Med Sci Sports Exerc. 2016;48(7):1244-50. | Ineligible participants |
| Lundstrom CJ, Russell HC, O'Donnell KJ, Ingraham SJ. Core and plyometric training for recreational marathon runners: effects on training variables, injury, and muscle damage. Sport Sciences for Health. 2019;15(1):167-74. | Ineligible outcomes |
| Lutter L. Injuries in the runner and jogger. Minn Med. 1980;63(1):45-51. | Ineligible study design |
| Lutter LD. Foot-related knee problems in the long distance runner. Foot Ankle. 1980;1(2):112-6. | Ineligible study design |
| Lutter LD. Cavus foot in runners. Foot Ankle. 1981;1(4):225-8. | Ineligible study design |
| Lysholm J, Wiklander J. Injuries in runners. Am J Sports Med. 1987;15(2):168-71. | Ineligible participants |
| Lysholm J, Wiklander J, Lysholm M. Injuries in runners in relation to findings on a pre-season physical examination. Italian Journal of Sports Traumatology. 1986;8(3):141-8. | Ineligible participants |
| Maas E, Vanwanseele B. Changes in running kinematics and kinetics after a 12-week running program for beginners. Sports Biomech. 2019:1-11. | Missing information |
| Macera CA, Pate RR, Powell KE, Jackson KL, Kendrick JS, Craven TE. Predicting lower-extremity injuries among habitual runners. Arch Intern Med. 1989;149(11):2565-8. | Ineligible outcomes |
| MacKelvie KJ, Taunton JE, McKay HA, Khan KM. Bone mineral density and serum testosterone in chronically trained, high mileage 40-55 year old male runners. BJSM online. 2000;34(4):273-8. | Ineligible study design |
| Major NM, Helms CA. Sacral stress fractures in long-distance runners. AJR Am J Roentgenol. 2000;174(3):727-9. | Ineligible study design |
| Malisoux L, Chambon N, Delattre N, Gueguen N, Urhausen A, Theisen D. Injury risk in runners using standard or motion control shoes: a randomised controlled trial with participant and assessor blinding. BJSM online. 2016;50(8):481-7. | Ineligible study design |
| Malisoux L, Nielsen RO, Urhausen A, Theisen D. A step towards understanding the mechanisms of running-related injuries. J Sci Med Sport. 2015;18(5):523-8. | Ineligible outcomes |
| Malisoux L, Ramesh J, Mann R, Seil R, Urhausen A, Theisen D. Can parallel use of different running shoes decrease running-related injury risk? Scand J Med Sci Sports. 2015;25(1):110-5. | Ineligible outcomes |
| Marshall, C. 2013. Minimalist versus conventional running shoes : effects on lower limb injury incidence, pain and muscle function experienced distance runners. University of Cape Town. | Missing information |
| Marshall RN. Foot mechanics and joggers' injuries. N Z Med J. 1978;88(621):288-90. | Ineligible study design |
| Marti B. Benefits and risks of running among women: an epidemiologic study. Int J Sports Med. 1988;9(2):92-8. | Ineligible outcomes |
| Marti B, Abelin T, Schoch O. [Epidemiology of running-induced complaints of joggers. Berne runner study '84]. Schweiz Med Wochenschr. 1986;116(18):603-8. | Ineligible outcomes |
| Masters KS, Lambert MJ. The Relations Between Cognitive Coping Strategies, Reasons for Running, Injury, and Performance of Marathon Runners. J Sport Exerc Psychol. 1989;11(2):161-70. | Ineligible outcomes |
| Matias AB, Taddei UT, Duarte M, Sacco ICN. Protocol for evaluating the effects of a therapeutic foot exercise program on injury incidence, foot functionality and biomechanics in long-distance runners: A randomized controlled trial. BMC Musculoskelet Disord. 2016;17 (1) (no pagination)(160). | Ineligible study design |
| Matos S, Ferreira da Silva BA, Clemente FM, et al. Running-related injuries in Portuguese trail runners: a retrospective cohort study. The Journal of sports medicine and physical fitness 2020;22 | Ineligible study design |
| Mattila VM, Sillanpaa PJ, Salo T, Laine HJ, Maenpaa H, Pihlajamaki H. Can orthotic insoles prevent lower limb overuse injuries? A randomized-controlled trial of 228 subjects. Scand J Med Sci Sports. 2011;21(6):804-8. | Ineligible participants |
| Mattock J, Steele J, Mickle K. Does tibial bone mineral status quality differ between medial tibial stress syndrome symptomatic and asymptomatic long-distance runners? Journal of Science and Medicine in Sport. 2019;22 (Supplement 2):S20. | Ineligible study design |
| Maynard J, Montero D, Pantin SA, Dematas K, Bittencourt E, Haak I, et al. Baseline characteristics of runners in a prospective study evaluating injuries during long-distance race training. Clin J Sport Med. 2014;24 (2):188. | Missing information |
| McClay MH, Appleby DC, Plascak FD. Predicting injury in young cross country runners with the Self-Motivation Inventory. Sports Training, Medicine and Rehabilitation. 1989;1(3):191-5. | Ineligible participants |
| McCormick F, Nwachukwu B, Provencher M. Stress fractures in runners. Clin Sports Med. 2012;31(2):291-306. | Ineligible study design |
| McCrory JL, Martin DF, Lowery RB, Cannon DW, Curl WW, Read HM, Jr., et al. Etiologic factors associated with Achilles tendinitis in runners. Med Sci Sports Exerc. 1999;31(10):1374-81. | Ineligible study design |
| McCurdy B. Does Running In Minimalist Shoes Increase Injury Risk? Podiatry Today. 2014;27(2):18-. | Missing information |
| McCurdy B. Study Assesses Injuries And Injury Rates Among Barefoot And Shod Runners. Podiatry Today. 2015;28(8):18-. | Ineligible study design |
| McElheny K, Toresdahl B, Metzl J, Ammerman B, Chang B, Kinderknecht J. First-time marathon completion and the effect of a self-directed strengthening program. Clin J Sport Med. 2018;28(2):217. | Ineligible study design |
| McKelvie SJ, Valliant PM, Asu ME. Physical training and personality factors as predictors of marathon time and training injury. Percept Mot Skills. 1985;60(2):551-66. | Ineligible outcomes |
| McKenzie D, Taunton J, Clement D. The prevention of running injuries. Aust J Sci Med Sports. 1986;18(2):7-8. | Ineligible study design |
| McKenzie DC, Clement DB, Taunton JE. Running shoes, orthotics, and injuries. Sports Med. 1985;2(5):334-47. | Ineligible study design |
| McNamee J. Overuse injury of the legs. Med J Aust. 1978;1(8):426-30. | Ineligible outcomes |
| Meardon SA. Skeletal loading: implications for injury and treatment: Iowa State University; 2009. | Ineligible participants |
| Medhat MA, Redford JB. Knee injuries. Damage from running and related sports. J Kans Med Soc. 1983;84(7):379-83, 413. | Ineligible study design |
| Messier S, Davis S, Curl W, Lowery R, Pack R. Etiologic factors associated with patellofemoral pain in runners. Medicine and Science in Sports and Exercise. 1991;23(9):1008-15. | Ineligible study design |
| Messier SP, Legault C, Schoenlank CR, Newman JJ, Martin DF, DeVita P. Risk factors and mechanisms of knee injury in runners. Med Sci Sports Exerc. 2008;40(11):1873-9. | Ineligible outcomes |
| Metzl J, Fontana MA, Quijano B, et al. Injury, illness and race performance of masters runners at the New York city marathon. Clin J Sport Med 2020;30 (2):190 | Ineligible outcomes |
| Michaud TC. Aberrancy of the midtarsal locking mechanism as a causative factor in recurrent ankle sprains. Journal of Manipulative and Physiological Therapeutics. 1989;12(2):135-41. | Ineligible study design |
| Micheli LJ. Lower extremity overuse injuries. Acta Med Scand. 1986;220(SUPPL. 711):171-7. | Ineligible study design |
| Miller E, Beck J, Runestad S, et al. Concurrent sports participation and prior marathon experience are not risk factors for injury in adolescent marathoner runners. Clin J Sport Med 2020;30 (2):172. | Ineligible outcomes |
| Mirkin G. The prevention and treatment of running injuries. J Am Podiatry Assoc. 1976;66(11):880-4. | Ineligible study design |
| Mitchell U, Johnson W, Ridge S, Standifird T. 'Natural Running' - Joggen im Minimalschuh: Fallstricke und Risiken. Zeitschrift fur Physiotherapeuten Krankengymnastik. 2012;64(7):47-9. | Ineligible study design |
| Moen MH, Bongers T, Bakker EW, Zimmermann WO, Weir A, Tol JL, et al. Risk factors and prognostic indicators for medial tibial stress syndrome. Scand J Med Sci Sports. 2012;22(1):34-9. | Ineligible participants |
| Mohammadi E, Saberi A. The relationship between body composition, anthropometry, and physical fitness in female university students. Trends in Sport Sciences. 2016;23(3):155-8. | Ineligible outcomes |
| Mohseni MM, Filmalter SE, Taylor WC, et al. Factors Associated With Half- and Full-Marathon Race-Related Injuries: A 3-Year Review. Clinical journal of sport medicine : official journal of the Canadian Academy of Sport Medicine 2019;18 | Ineligible outcomes |
| Mohseni M, Thomas C, Diehl N, Vadeboncoeur T. Risk factors associated with development of race-related injuries in a community marathon and half-marathon. Clin J Sport Med. 2011;21 (4):382. | Ineligible outcomes |
| Mohseni M, Vadeboncoeur T, McNeil R, Silvers S, Diehl N, Shapiro S, et al. Runners' training profiles and risk of lower extremity injury: A two year experience. Acad Emerg Med. 2010;17:S166-S7. | Ineligible outcomes |
| Montalbano MM, Hugar DW. Metatarsal stress fractures in runners. J Am Podiatry Assoc. 1982;72(11):581-3. | Ineligible study design |
| Morrissey D, Padhiar N. Leg and related ankle injuries. SportEX Medicine. 2000(4):27-32. | Missing information |
| Moshitta MT, Finley J. The clinic. Hip pain after long runs. Running & FitNews. 1999;17(2):7-. | Ineligible study design |
| Muhr G, Richter J. [The upper ankle joint--a "permanent runner"]. Orthopade. 1999;28(6):459. | Ineligible study design |
| Mulvad B, Nielsen RO, Lind M, Ramskov D. Diagnoses and time to recovery among injured recreational runners in the RUN CLEVER trial. PLoS ONE. 2018;13(10):e0204742. | Ineligible outcomes |
| Murphy P. Orthoses: Not the sole solution for running ailments. Physician and Sportsmedicine. 1986;14(2):164-70. | Ineligible study design |
| Myer GD, Ford KR, Barber Foss KD, Chunyan L, Nick TG, Hewett TE. The Relationship of Hamstrings and Quadriceps Strength to Anterior Cruciate Ligament Injury in Female Athletes. Clin J Sport Med. 2009;19(1):3-8. | Ineligible study design |
| Myers RA, Khodaee M, Spittler J, Hill J, Lee J, Yeakel D. Does foot type or shoe type affect injury rate in an ultra-endurance trail run? Clin J Sport Med. 2011;21 (2):170. | Ineligible outcomes |
| Naderi A, Moen MH, Degens H. Is high soleus muscle activity during the stance phase of the running cycle a potential risk factor for the development of medial tibial stress syndrome? A prospective study. J Sports Sci 2020;38(20):2350-58. | Ineligible participants (confirmed by contact author) |
| Nadler SF, Malanga GA, Feinberg JH, Prybicien M, Stitik TP, DePrince M. Relationship between hip muscle imbalance and occurrence of low back pain in collegiate athletes: a prospective study. Am J Phys Med Rehabil. 2001;80(8):572-7. | Ineligible participants |
| Nakajima M, Wu WFW, Becker J. Factors Contributing to Medial Tibial Stress Syndrome in Runners: A Prospective Study. Med Sci Sports Exerc. 2018;50(10):2092-100. | Ineligible participants |
| Nakhaee Z, Rahimi A, Abaee M, Rezasoltani A, Kalantari K. The relationship between the height of the medial longitudinal arch (MLA) and the ankle and knee injuries in professional runners. Foot. 2008;18(2):84-90. | Ineligible study design |
| Nam-Ku L, Jong-Kyu K, Eung-Joon K, Sun-Kyung K, In-ho C, Joon-Yong C, et al. The Relationship Among Femoral Neck Angle and BMD, and Lower Extremity Injury Incidence in Elite Athletes: Based Upon Gender. International Journal of Applied Sports Sciences. 2011;23(2):383-93. | Ineligible participants |
| Napier C. A clinical gait retraining approach to reducing kinetic risk factors of running-related injury. Journal of Science and Medicine in Sport. 2018;21(Supplement 1):S4. | Ineligible study design |
| Napier C, MacLean C, Maurer J, et al. Real-time biofeedback of performance to reduce braking forces associated with running-related injury: An exploratory study. Journal of Orthopaedic and Sports Physical Therapy 2019;49(3):136-44. | Ineligible study design |
| Nathan J, Silman A. Injury Prevention in Marathon Runners. Muscles, Ligaments & Tendons Journal (MLTJ). 2012:78-9. | Ineligible outcomes |
| Nicholas JA, Marino M. The relationship of injuries of the leg, foot, and ankle to proximal thigh strength in athletes. Foot Ankle. 1987;7(4):218-28. | Ineligible study design |
| Nielsen RO, Bertelsen ML, Hansen M, Rasmussen S. The start-to-run distance and running-related injury among obese novice runners: a randomized trial. International Journal of Sports Physical Therapy. 2018;13(6):943-55. | Ineligible outcomes |
| Nielsen O, Parner E, Nohr E, Sorensen H, Lind M, Rasmussen S. Excessive progression in weekly running distance and risk of running-related injuries: An association which varies according to type of injury. Journal of Orthopaedic and Sports Physical Therapy. 2014;44(10):739-47. | Ineligible outcomes |
| Nielsen R, Buist I, Parner E, Nohr E, Sorensen H, Lind M, et al. Foot pronation is not associated with increased injury risk in novice runners wearing a neutral shoe: A 1-year prospective cohort study. BJSM online. 2014;48(6):440-7. | Ineligible study design |
| Nielsen RO, Bertelsen ML, Parner ET, Sørensen H, Lind M, Rasmussen S. Running more than three kilometers during the first week of a running regimen may be associated with increased risk of injury in obese novice runners. International Journal of Sports Physical Therapy. 2014;9(3):338-45. | Ineligible outcomes |
| Nielsen RO, Buist I, Parner ET, Nohr EA, Sorensen H, Lind M, et al. Predictors of running-related injuries among 930 novice runners: A 1-year prospective follow-up study. Orthopaedic Journal of Sports Medicine. 2013;1(1). | Ineligible outcomes |
| Nielsen RO, Ronnow L, Rasmussen S, Lind M. A prospective study on time to recovery in 254 injured novice runners. PLoS ONE. 2014;9(6):e99877. | Ineligible outcomes |
| Nilsson S. Overuse knee injuries in runners. Int J Sports Med. 1984;5(SUPPL.):145-8. | Ineligible study design |
| Nitzschke E, Leonhardt R. [Jogging--stress-induced damage of the musculoskeletal system]. Sportverletz Sportschaden. 1991;5(1):22-6. | Ineligible outcomes |
| Nix RA. Runners' injuries about the knee. J Ark Med Soc. 1982;79(6):200-5. | Ineligible study design |
| Noehren B, Davis I. The effect of gait retraining on hip mechanics, pain, and function in runners with patellofemoral pain syndrome...Patellofemoral pain syndrome: proximal, distal, and local factors, an international research retreat, April 30-May 2, 2009, Fells Point, Baltimore, MD. J Orthop Sports Phys Ther. 2010;40(3):A40-1. | Ineligible outcomes |
| Novak J, Bojanovsky I. [Disorders of the lower extremities in top marathon runners]. Acta Chir Orthop Traumatol Cech. 1987;54(5):449-53. | Ineligible outcomes |
| Nye ER. Some hazards of heavy exercise: jogging and running--is it all good? N Z Med J. 1986;99(813):847. | Ineligible study design |
| Ogwumike OO, Adeniyi AF. The SPLASH/ICPC integrity marathon in Ibadan, Nigeria: Incidence and management of injuries and marathon-related health problems. BMC Sports Science, Medicine and Rehabilitation. 2013;5 (1) (no pagination)(6). | Ineligible outcomes |
| Onieal M. Running injuries. J Am Acad Nurse Pract. 1994;6(2):85-7. | Ineligible study design |
| Onieal ME. Shin splints. J Am Acad Nurse Pract. 1994;6(5):214-5. | Ineligible study design |
| Opar D, Drezner J, Shield A, Sennett B, Kapur R, Cohen M, et al. A 5-year study into hamstring strain injuries at the Penn Relay Carnivala. Journal of Science and Medicine in Sport. 2012;15:S131. | Ineligible study design |
| Opar DA, Drezner J, Shield A, Williams M, Webner D, Sennett B, et al. Acute hamstring strain injury in track-and-field athletes: A 3-year observational study at the Penn Relay Carnival. Scand J Med Sci Sports. 2014;24(4):e254-9. | Ineligible study design |
| Orava S. Overexertion injuries in keep-fit athletes. A study on overexertion injuries among non-competitive keep-fit athletes. Scand J Rehabil Med. 1978;10(4):187-91. | Ineligible outcomes |
| ØStergaard Nielsen R, Thorlund Parner E, Aagaard Nohr E, SØRensen H, Lind M, Rasmussen S. Excessive Progression in Weekly Running Distance and Risk of Running-Related Injuries: An Association Which Varies According to Type of Injury. J Orthop Sports Phys Ther. 2014;44(10):739-47. | Ineligible study design |
| Östör AJK, Conaghan PG. Is there a relationship between running and osteoarthritis? International SportMed Journal. 2006;7(2):75-84. | Ineligible study design |
| O'Toole M. Prevention and treatment of injuries to runners. Medicine and Science in Sports and Exercise. 1992;24(9 Suppl):S360-1. | Ineligible study design |
| Padhiar N. Injuries to the foot in endurance sport. SportEX Medicine. 2001(8):11-5. | Ineligible study design |
| Pagliano J. Running: examination, diagnosis, and treatment. Clin Podiatr Med Surg. 1986;3(4):661-70. | Ineligible study design |
| Pagliano JW, Jackson DW. A clinical study of 3,000 long-distance runners. Annals of Sports Medicine. 1987;3(2):88-91. | Ineligible study design |
| Parfitt P. Comparison of running injuries between middle distance and marathon runners. J Br Podiatr Med. 1994;49(8):133-6. | Ineligible study design |
| Parker DT, Weitzenberg TW, Amey AL, Nied RJ. Group training programs and self-reported injury risk in female marathoners. Clin J Sport Med. 2011;21(6):499-507. | Ineligible study design |
| Pauls C, Kravitz L. Barefoot running: an exciting new training dimension to consider for certain clients. IDEA Fitness Journal. 2010;7(4):18-20. | Ineligible study design |
| Pecci M. Knee pain in the recreational athlete. Family Practice Recertification. 2007;29(11):45-50. | Ineligible study design |
| Pedoe DT. Prevention of injury in joggers and runners. Practitioner. 1988;232(1456 ( Pt 1)):1109-12. | Ineligible study design |
| Perle SM. Sports chiropractic. Runner's pelvis. Chiropractic Journal. 1989;3(5):13-. | Ineligible study design |
| Philipson MR, Parker PJ. Stress fractures. Orthopaedics and Trauma. 2009;23(2):137-43. | Ineligible study design |
| Phinyomark A, Osis S, Hettinga B, Leigh R, Ferber R. Gender differences in gait kinematics in runners with iliotibial band syndrome. Scandinavian Journal of Medicine and Science in Sports. 2015;25(6):744-53. | Ineligible study design |
| Phinyomark A, Osis S, Hettinga BA, Ferber R. Kinematic gait patterns in healthy runners: A hierarchical cluster analysis. J Biomech. 2015;48(14):3897-904. | Ineligible study design |
| Pisani G. Chronic foot injuries in runners aged over 50 years. Chirurgia del Piede. 2010;34(2):91-8. | Ineligible study design |
| Piterman L. The hazards of jogging and running. Aust Fam Physician. 1982;11(12):943-8. | Ineligible study design |
| Plastaras CT, Rittenberg JD, Rittenberg KE, Press J, Akuthota V. Comprehensive functional evaluation of the injured runner. Phys Med Rehabil Clin N Am. 2005;16(3):623-49. | Ineligible study design |
| Platou CS. [Traction periostitis of the tibia]. Tidsskr Nor Laegeforen. 1982;102(5):308-9. | Ineligible study design |
| Plisky M, Rauh M, Heiderscheit B, Underwood F, Tank R. Medial Tibial Stress Syndrome in High School Cross-Country Runners:, Incidence and Risk Factors. Journal of Orthopaedic and Sports Physical Therapy. 2007;37(2):40-7. | Ineligible participants |
| Poppel D, Koning J, Verhagen AP, Scholten-Peeters GGM. Risk factors for lower extremity injuries among half marathon and marathon runners of the Lage Landen Marathon Eindhoven 2012: A prospective cohort study in the Netherlands. Scand J Med Sci Sports. 2016;26(2):226-34. | Ineligible outcomes |
| Poppel D, Scholten-Peeters GGM, Middelkoop M, Verhagen AP. Prevalence, incidence and course of lower extremity injuries in runners during a 12-month follow-up period. Scand J Med Sci Sports. 2014;24(6):943-9. | Ineligible outcomes |
| Powell DW, Williams 3rd DSB, Windsor B, Butler RJ, Zhang S. Ankle work and dynamic joint stiffness in high- compared to low-arched athletes during a barefoot running task. Hum Mov Sci. 2014;34:147-56. | Ineligible study design |
| Powell KE, Kohl HW, Caspersen CJ, Blair SN. An epidemiological perspective on the causes of running injuries. Physician and Sportsmedicine. 1986;14(6):100-14. | Ineligible study design |
| Powers CM, Berke GM, Clary MD, Fredericson M. Patellofemoral Pain: Is There a Role for Orthoses? PM and R. 2010;2(8):771-6. | Ineligible study design |
| Price MD, Herndon JH. Is running good for your knees? Menopause. 2007;14(5):815-6. | Ineligible study design |
| Prost MA. Stressed out: stress fractures are one of the most common problems among athletes, especially long-distance runners. Advance for Directors in Rehabilitation. 1999;8(9):60-5. | Missing information |
| Pujalte GG, Silvis ML. The injured runner. Med Clin North Am. 2014;98(4):851-68, xiii. | Ineligible study design |
| Quirk R. Stress fractures of the foot. Aust Fam Physician. 1987;16(8):1101-2. | Ineligible study design |
| Rahnama N. Preventing sport injuries: Improving performance. International Journal of Preventive Medicine. 2012;3(3):143-4. | Ineligible study design |
| Ramsey C, Lamb P, Ribeiro D. Asymmetric footwear among running-related injuries. Journal of Science and Medicine in Sport. 2019;22 (Supplement 2):S18-S9. | Ineligible outcomes |
| Ramskov D, Barton C, Nielsen R, Rasmusen S. High eccentric hip abduction strength reduces the risk of developing, patellofemoral pain among novice runners initiating a self-structured running program: A 1-year observational study. Journal of Orthopaedic and Sports Physical Therapy. 2015;45(3):153-61. | Ineligible study design |
| Ramskov D, Jensen ML, Obling K, Nielsen RO, Parner ET, Rasmussen S. Original research. No association between q-angle and foot posture with runningrelated injuries: a 10 week prospective followup study. International Journal of Sports Physical Therapy. 2013;8(4):407-15. | Ineligible study design |
| Ramskov D, Nielsen RO, Sørensen H, Parner E, Lind M, Rasmussen S. The design of the run Clever randomized trial: running volume, -intensity and running-related injuries. BMC Musculoskelet Disord. 2016;17:1-11. | Ineligible study design |
| Ramskov D, Rasmussen S, SØRensen H, Thorlund Parner E, Lind M, Nielsen R. Progression in Running Intensity or Running Volume and the Development of Specific Injuries in Recreational Runners: Run Clever, a Randomized Trial Using Competing Risks. J Orthop Sports Phys Ther. 2018;48(10):740-A4. | Ineligible outcomes |
| Rauh MJ. Leg-length inequality and running-related injury among high school runners. International Journal of Sports Physical Therapy. 2018;13(4):643-51. | Ineligible participants |
| Rauh M. Summer training factors and risk of musculoskeletal injury among high school cross-country runners. Journal of Orthopaedic and Sports Physical Therapy. 2014;44(10):793-804. | Ineligible participants |
| Rauh M, Koepsell T, Rivara F, Rice S, Margherita A. Quadriceps Angle and Risk of Injury Among High School Cross-Country Runners. Journal of Orthopaedic and Sports Physical Therapy. 2007;37(12):725-33. | Ineligible participants |
| Rauh MJ, Barrack M, Nichols JF. Associations between the fremale athlete triad and injury among high school runners. International Journal of Sports Physical Therapy. 2014;9(7):948-58. | Ineligible participants |
| Rauh MJ, Beachy G. High school girls' cross-country running injuries: a 20-year longitudinal study. J Orthop Sports Phys Ther. 2009;39(1):A110-1. | Ineligible participants |
| Rauh MJ, Koepsell TD, Rivara FP, Margherita AJ, Rice SG. Epidemiology of musculoskeletal injuries among high school cross-country runners. Am J Epidemiol. 2006;163(2):151-9. | Ineligible participants |
| Rauh MJ, Margherita AJ, Rice SG, Koepsell TD, Rivara FP. High school cross country running injuries: a longitudinal study. Clin J Sport Med. 2000;10(2):110-6. | Ineligible outcomes |
| Rauh MJ, Nichols JF, Barrack MT. Associations of lower extremity injury with disordered eating, menstrual dysfunction, and low bone mineral density among female high school track and field and cross-country runners. J Orthop Sports Phys Ther. 2009;39(1):A110-A. | Ineligible participants |
| Rauh MJ, Nichols JF, Barrack MT. Relationships Among Injury and Disordered Eating, Menstrual Dysfunction, and Low Bone Mineral Density in High School Athletes: A Prospective Study. Journal of Athletic Training (National Athletic Trainers' Association). 2010;45(3):243-52. | Ineligible outcomes |
| Rauh MJ, Nichols JF, Barrack MT. Prevalence and relationships between disordered eating, menstrual dysfunction and musculoskeletal injury among competitive female interscholastic runners. J Orthop Sports Phys Ther. 2010;40(2):122-. | Ineligible participants |
| Rauh MJ KT. Preseason training habits and risk of injury among interscholastic cross-country runners. J Orthop Sports Phys Ther. 2011;41(1):A50-1. | Ineligible outcomes |
| Rauh MJ, Tenforde AS, Barrack MT, Rosenthal MD, Nichols JF. Associations Between Sport Specialization, Running-Related Injury, and Menstrual Dysfunction Among High School Distance Runners. Athletic Training & Sports Health Care: The Journal for the Practicing Clinician. 2018;10(6):260-9. | Ineligible participants |
| Rauh MJ, Tutino AK, Post EG, et al. Relationships between sport specialization and running-related injury among competitive high school cross-country runners. Orthopaedic Journal of Sports Medicine Conference: 7th Annual Meeting of the Pediatric Research in Sports Medicine Society, PRiSM 2020;8(4 Supplement 3) | Ineligible participants |
| Reinking M, Austin T, Hayes A. Exercise-Related Leg Pain in Collegiate Cross-Country Athletes: Extrinsic and Intrinsic Risk Factors. Journal of Orthopaedic and Sports Physical Therapy. 2007;37(11):670-8. | Missing information |
| Reinking M, Hayes A, Austin T. The effect of foot orthotic use on exercise related leg pain in cross country athletes. Phys Ther Sport. 2012;13(4):214-8. | Ineligible study design |
| Reinking MF. Exercise-related leg pain in female collegiate athletes: the influence of intrinsic and extrinsic factors. Am J Sports Med. 2006;34(9):1500-7. | Missing information |
| Reinking MF, Austin TM, Bennett J, Hayes AM, Mitchell WA. Lower extremity overuse bone injury risk factors in collegiate athletes: a pilot study. International Journal of Sports Physical Therapy. 2015;10(2):155-67. | Missing information |
| Reinking MF, Austin TM, Hayes AM. Exercise related leg pain in high school cross-country athletes: intrinsic and extrinsic risk factors...2008 Combined Sections Meeting...Nashville, Tennessee, February 6-9, 2008. J Orthop Sports Phys Ther. 2008;38(1):A76-A. | Missing information |
| Reinking MF, Austin TM, Hayes AM. Risk factors for self-reported exercise-related leg pain in high school cross-country athletes. J Athlet Train. 2010;45(1):51-7. | Missing information |
| Reinking MF, Hayes AM. Intrinsic factors associated with exercise-related leg pain in collegiate cross-country runners. Clin J Sport Med. 2006;16(1):10-4. | Missing information |
| Requa RK, DeAvilla LN, Garrick JG. Injuries in recreational adult fitness activities. Am J Sports Med. 1993;21(3):461-7. | Ineligible participants |
| Reul CA, Al WW. The relevance of subtalar-joint-anatomy for chronic overuse injuries of the lower limbs. Foot and Ankle International. 2011;32 (3):321. | Ineligible study design |
| Reule CA, Alt WW, Lohrer H, Hochwald H. Spatial orientation of the subtalar joint axis is different in subjects with and without Achilles tendon disorders. BJSM online. 2011;45(13):1029-34. | Ineligible study design |
| Reynolds KL, Harman EA, Worsham RE, Sykes MB, Frykman PN, Backus VL. Injuries in women associated with a periodized strength training and running program. J Strength Cond Res. 2001;15(1):136-43. | Ineligible study design |
| Ricker-Fox J. Évolution de la relation athlète/entraîneur. Coaches Plan/Plan du Coach. 2010;17(2):39-. | Ineligible study design |
| Riddle D, Pulisic M, Pidcoe P, Johnson R. Risk factors for plantar fasciitis: a matched case-control study. Journal of Bone and Joint Surgery (American). 2003;85(5):872-7. | Ineligible study design |
| Ridge ST, Johnson AW, Mitchell UH, Hunter I, Robinson E, Rich BS, et al. Foot bone marrow edema after a 10-wk transition to minimalist running shoes. Med Sci Sports Exerc. 2013;45(7):1363-8. | Ineligible outcomes |
| Ristolainen L, Kettunen J, Waller B, Heinonen A, Kujala U. Training-related risk factors in the etiology of overuse injuries in endurance sports. J Sports Med Phys Fitness. 2014;54(1):78-87. | Ineligible study design |
| Rixe J, Silvis M. Impact of a digital running intervention on minimalist running-related injuries. Clin J Sport Med. 2014;24 (2):190. | Ineligible study design |
| Roan S. OK, let's take a closer look at that stride: gait analysis can help runners improve their performance -- and reduce injury [corrected] [published erratum appears in LA TIMES 2007 Jan 8; Health:F5]. Los Angeles Times -- Southern California Edition (Front Page). 2007:F6-F. | Ineligible study design |
| Robbins S, Hanna D. Running-related injury prevention through barefoot adaptations. Medicine and Science in Sports and Exercise. 1987;19(2):148-56. | Ineligible study design |
| Robbins SE, Gouw GJ, Hanna AM. Running-related injury prevention through innate impact-moderating behavior. Med Sci Sports Exerc. 1989;21(2):130-9. | Ineligible study design |
| Roberts D. Learning to train the myofascial system: understanding tibial fractures and medial tibial stress syndrome. Massage Today. 2012;12(12):15-7. | Ineligible study design |
| Rochcongar P, Pernes J, Carre F, Chaperon J. Occurrence of running injuries: A survey among 1153 runners. [French]. Science and Sports. 1995;10(1):15-9. | Ineligible outcomes |
| Rodrigues P. Consequence of functioning at the end range of joint motion: Implications on anterior knee pain: University of Massachusetts Amherst; 2011. | Ineligible study design |
| Rolfe B. lifestyle. run THE RIGHT WAY. Alive: Canada's Natural Health & Wellness Magazine. 2014(378):127-32. | Ineligible study design |
| Rome K, Hancock D, Poratt D. Barefoot running and walking: The pros and cons based on current evidence. N Z Med J. 1972;121(1272). | Ineligible study design |
| Ropiak CR, Bosco JA. Hamstring injuries. Bulletin of the NYU Hospital for Joint Diseases. 2012;70(1):41-8. | Ineligible study design |
| Ross CF, Schuster RO. A preliminary report on predicting injuries in distance runners. J Am Podiatry Assoc. 1983;73(5):275-7. | Missing information |
| Roughny P. Barefoot vs. shod: middle ground? Advance for Physical Therapy & Rehab Medicine. 2012;23(7):11-. | Missing information |
| Rowlands AV, Eston RG, Tilzey C. Effect of stride length manipulation on symptoms of exercise-induced muscle damage and the repeated bout effect. J Sports Sci. 2001;19(5):333-40. | Ineligible outcomes |
| Rowlands C, Plumb MS. The effects of a 4-week barefoot exercise intervention on plantar pressure, impact, balance and pain in injured recreational runners: A pilot study. International Journal of Osteopathic Medicine 2019;33-34:1-7. | Ineligible participants |
| Royer M, Thomas T, Cesini J, Legrand E. Stress Fractures in 2011: Practical Approach. Joint Bone Spine. 2012;79(SUPPL. 2):S86-S90. | Ineligible study design |
| Rudy EB, Estok PJ. Specific areas of concern for the female jogger. Occup Health Nurs. 1985;33(10):496-9. | Ineligible study design |
| Ruffe NJ, Sorce SR, Rosenthal MD, Rauh MJ. Lower quarter- and upper quarter y balance tests as predictors of running-related injuries in high school cross-country runners. International Journal of Sports Physical Therapy. 2019;14(5):695-706. | Missing information |
| Ryan M, Elashi M, Newsham-West R, Taunton J. Examining injury risk and pain perception in runners using minimalist footwear. BJSM online. 2014;48(16):1257-62. | Ineligible study design |
| Ryan M, Elashi M, Taunton J, Koehl M. Is gender a risk factor for injury in runners? The first of a three part series to develop a gender targeted injury prevention strategy. Journal of Science and Medicine in Sport. 2014;18:e72-e3. | Missing information |
| Saillant G, Rodineau J, Roy-Camille R, Benazet JP, Sabourin F. [Prevention, diagnosis and treatment of osteoarticular and musculotendinous complications related to the practice of running]. Rev Prat. 1985;35(7):367-70, 73-5. | Ineligible study design |
| Saka T, Yildiz Y. Exercise-induced lower leg pain: medical education. Turkiye Klinikleri Journal of Medical Sciences. 2007;27(5):753-62. | Ineligible study design |
| Salmon K, Miller GK, Daniels J. The clinic. Hill running & knee problems. Running & FitNews. 2000;18(10):7-. | Ineligible study design |
| Salzler MJ, Kirwan HJ, Scarborough DM, Walker JT, Guarino AJ, Berkson EM. Injuries observed in a prospective transition from traditional to minimalist footwear: correlation of high impact transient forces and lower injury severity. Physician and Sportsmedicine. 2016;44(4):373-9. | Ineligible study design |
| Satterthwaite P. Incidence of injuries and other health problems in the Auckland Citibank marathon, 1993. BJSM online. 1996;30(4):324-6. | Ineligible outcomes |
| Satterthwaite P, Norton R, Larmer P, Robinson E. Risk factors for injuries and other health problems sustained in a marathon. BJSM online. 1999;33(1):22-6. | Ineligible outcomes |
| Schiffer T, Montiel G, Hildebrandt U, Predel HG, Knackstedt C. Marathon race: A health risk? Internistic and orthopedic aspects. [German]. Klinikarzt. 2010;39(6):288-91. | Ineligible study design |
| Schilders E. Groin injuries in athletes. Current Orthopaedics. 2000;14(6):418-23. | Ineligible study design |
| Schlunsen C. High tech athletic shoes. Foot form and running style determine the shoe. [German]. Z Orthop Ihre Grenzgeb. 1998;136(4):Oa18-22. | Ineligible study design |
| Schoene LM. What You Need to Know About Piriformis Syndrome. Podiatry Management. 2012;31(5):209-12. | Ineligible study design |
| Schwabe K, Schwellnus M, Derman W, Swanevelder S, Jordaan E. Medical complications and deaths in 21 and 56 km road race runners: a 4-year prospective study in 65 865 runners--SAFER study I. BJSM online. 2014;48(11):912-8. | Ineligible outcomes |
| Schwabe K, Schwellnus MP, Derman W, Swanevelder S, Jordaan E. Less experience and running pace are potential risk factors for medical complications during a 56 km road running race: a prospective study in 26 354 race starters--SAFER study II. BJSM online. 2014;48(11):905-11. | Ineligible outcomes |
| Schwabe K, Schwellnus MP, Derman W, Swanevelder S, Jordaan E. Older females are at higher risk for medical complications during 21 km road race running: a prospective study in 39 511 race starters--SAFER study III. BJSM online. 2014;48(11):891-7. | Ineligible outcomes |
| Schwellnus M, Derman W. The quest to reduce the risk of adverse medical events in exercising individuals: introducing the SAFER (Strategies to reduce Adverse medical events For the ExerciseR) studies. BJSM online. 2014;48(11):869-70. | Ineligible study design |
| Scott FN, Malanga GA, Solomon JL, Feinberg JH, Foye PM, Park YI. The relationship between lower extremity injury and the hip abductor to extensor strength ratio in collegiate athletes. Journal of Back & Musculoskeletal Rehabilitation. 2002;16(4):153-8. | Ineligible study design |
| Scott G, Zehnacker C, Cianca J. Calf muscle injury. Running & FitNews. 2006;24(1):13-4. | Ineligible study design |
| Segesser B, Nigg BM. [Tibial insertion tendinoses, achillodynia, and damage due to overuse of the foot-etiology, biomechanics, therapy (author's transl)]. Orthopade. 1980;9(3):207-14. | Ineligible study design |
| Senda M, Takahara Y, Yagata Y, Yamamoto K, Nagashima H, Tukiyama H, et al. Measurement of the muscle power of the toes in female marathon runners using a toe dynamometer. Acta Med Okayama. 1999;53(4):189-91. | Ineligible study design |
| Sepulchre P, Blaimont P, Pasteels JL. [Internal tibial pain in runners]. Phlebologie. 1988;41(3):664-72. | Ineligible study design |
| Sepulchre P, Blaimont P, Pasteels JL. [Medial tibial pain in runners]. Int Orthop. 1988;12(3):217-21. | Ineligible study design |
| Shanthikumar S, Low Z, Falvey E, McCrory P, Baker R, Franklyn-Miller A. The effect of gait velocity on calcaneal balance at heel strike: implications for orthotic prescription in injury prevention. BJSM online. 2008;42(6):542-3. | Ineligible outcomes |
| Sheehan GA. Chondromalacia in runners. J Med Soc N J. 1972;69(6):527-8. | Ineligible study design |
| Sheehan GA. An overview of overuse syndromes in distance runners. Ann N Y Acad Sci. 1977;301:877-80. | Ineligible study design |
| Sherbondy P, Sebastianelli W. Stress fractures of the medial malleolus and distal fibula. Clin Sports Med. 2006;25(1):129-37, x. | Ineligible study design |
| Sherkin KJ. The heavyweight runner. J Am Podiatr Med Assoc. 1987;77(9):517-9. | Ineligible study design |
| Singh S, Fredericson M, Sainani K, et al. Sleep correlates with faster mile time and increased triad risk factors in female runners, a 5-year exploratory analysis. Clin J Sport Med 2019;29 (2):167-68 | Ineligible outcomes |
| Shih YF, Wen YK, Chen WY. Application of wedged foot orthosis effectively reduces pain in runners with pronated foot: a randomized clinical study. Clin Rehabil. 2011;25(10):913-23. | Ineligible participants |
| Siegel IM. Jogger's heel. Jama. 1968;206(13):2899. | Ineligible study design |
| Siegele J, Horstmann T, Bunc V, Shifta P, Verle S, Niess A. [Relation between pelvis malposition and functional knee pain by long distance running]. Sportverletz Sportschaden. 2010;24(3):144-9. | Ineligible study design |
| Silva VBd, Lara S, Teixeira LP, de Souza Balk R. Análise do desempenho funcional dos músculos extensores e flexores do joelho em corredores de rua. ConScientiae Saude. 2019;19(2):157-64. | Ineligible study design |
| Smith LS, Bunch R. Athletic footwear. Clin Podiatr Med Surg. 1986;3(4):637-47. | Ineligible study design |
| Smith S. Illiotibial band syndrome. California Chiropractic Association Journal. 2010;35(4):17-9. | Ineligible study design |
| Smith T. Runners focus on your feet. Running & FitNews. 1997;15(6):4-5. | Missing information |
| Smith T. Avoiding chronic injuries. Running & FitNews. 1998;16(4):4-5. | Missing information |
| Smits DW, Huisstede B, Verhagen E, Van Der Worp H, Kluitenberg B, Van Middelkoop M, et al. Short-Term Absenteeism and Health Care Utilization Due to Lower Extremity Injuries among Novice Runners: A Prospective Cohort Study. Clin J Sport Med. 2016;26(6):502-9. | Ineligible outcomes |
| Snyder AC, Clark N. Stress fractures of male distance runners: Lack of association with nutritional practices. Nutr Res. 1993;13(9):995-1002. | Ineligible study design |
| Sperryn P. Running injuries. Practitioner. 1989;233(1462):180-3. | Ineligible study design |
| St. Louis M. Navicular stress reactions in runners. J Am Podiatr Med Assoc. 2013;103(3):254. | Ineligible study design |
| Stacoff A. [Sports shoes "prevention and performance"]. Sportverletz Sportschaden. 2000;14(3):69-70. | Ineligible study design |
| Stasiewicz M, Ślężyński J. Sport injuries in runners. Polish Journal of Sports Medicine / Medycyna Sportowa 2020;36(1):247-59 | Ineligible outcomes |
| Staheli LT, Lippert F, Denotter P. Femoral anteversion and physical performance in adolescent and adult life. Clin Orthop. 1977(129):213-6. | Ineligible outcomes |
| Stefanyshyn DJ, Stergiou P, Nigg BM, Lun VMY, Meeuwisse WH. The relationship between impact forces and running injuries. Archives of Physiology and Biochemistry. 2000;108(1-2):43. | Missing information |
| Steinacker T, Steuer M, Holtke V. [Orthopedic problems in older marathon runners]. Sportverletz Sportschaden. 2001;15(1):12-5. | Ineligible participants |
| Steinberg N, Nemet D, Pantanowitz M, Zeev A, Hallumi M, Sindiani M, et al. Longitudinal Study Evaluating Postural Balance of Young Athletes. Percept Mot Skills. 2016;122(1):256-79. | Ineligible participants |
| Steingruber IE, Wolf C, Gruber H, Gabriel M, Czermak BV, Mallouhi A, et al. [Stress fractures in athletes]. Radiologe. 2002;42(10):771-7. | Ineligible study design |
| Stephan Y, Deroche T, Brewer BW, Caudroit J, Le Scanff C. Predictors of perceived susceptibility to sport-related injury among competitive runners: the role of previous experience, neuroticism, and passion for running. Applied Psychology: An International Review. 2009;58(4):672-87. | Ineligible study design |
| Stevinson C, Plateau CR, Plunkett S, et al. Adherence and Health-Related Outcomes of Beginner Running Programs: A 10-Week Observational Study. Research quarterly for exercise and sport 2020:1-9. | Ineligible outcomes |
| Subotnick SI. A biomechanical approach to running injuries. Ann N Y Acad Sci. 1977;301:888-99. | Ineligible study design |
| Subotnick SI. The biomechanics of running. Implications for the prevention of foot injuries. Sports Med. 1985;2(2):144-53. | Ineligible study design |
| Sugimoto D, Kelly BD, Mandel DL et al. Running propensities of athletes with hamstring injuries. Sports 2019;7(9):210 | Ineligible study design |
| Taddei UT, Matias AB, Duarte M, et al. Foot core training to prevent running-related injuries: a survival analysis of a single-blind, randomized controlled trial. The American journal of sports medicine 2020;48(14):3610-19. | Ineligible study design |
| Taddei UT, Matias AB, Ribeiro FIA, Inoue RS, Bus SA, Sacco ICN. Effects of a therapeutic foot exercise program on injury incidence, foot functionality and biomechanics in long-distance runners: Feasibility study for a randomized controlled trial. Physical Therapy in Sport. 2018;34:216-26. | Ineligible study design |
| Tam N, Santos-Concejero J, Coetzee DR, Noakes TD, Tucker R. Muscle co-activation and its influence on running performance and risk of injury in elite Kenyan runners. J Sports Sci. 2017;35(2):175-81. | Ineligible participants |
| Tam N, Tucker R, Astephen Wilson JL. Individual Responses to a Barefoot Running Program: Insight Into Risk of Injury. Am J Sports Med. 2016;44(3):777-84. | Ineligible outcomes |
| Tan CM, Tan IW, Kok WL, Lee MC, Lee VJ. Medical planning for mass-participation running events: a 3-year review of a half-marathon in Singapore. BMC Public Health. 2014;14:1109. | Ineligible study design |
| Taunton JE, Ryan MB, Clement DB, McKenzie DC, Lloyd-Smith DR, Zumbo BD. A prospective study of running injuries: the Vancouver Sun Run "In Training" clinics. BJSM online. 2003;37(3):239-44. | Ineligible outcomes |
| Teng HL, Dilauro A, Weeks C, et al. Short-term effects of a trunk modification program on patellofemoral joint stress in asymptomatic runners. Phys Ther Sport 2020;44:107-13. | Ineligible outcomes |
| Tersegno MM. Meniscal tears in marathon runners. AJR Am J Roentgenol. 1992;159(2):434. | Ineligible study design |
| Theisen D, Malisoux L, Genin J, Delattre N, Seil R, Urhausen A. Influence of midsole hardness of standard cushioned shoes on running-related injury risk. BJSM online. 2014;48(5):371-6. | Ineligible study design |
| Thorne D, Datz F. Pelvic stress fracture in female runners. Clin Nucl Med. 1986;11(12):828-9. | Ineligible study design |
| Tibbit C, Cianca J. Zero Impact Can Still Injure Knees. Running & FitNews. 2014;32(2):14-5. | Ineligible study design |
| Tietze D, Best TM. Injury Prevention in the Novice Runner. ACSM's Health & Fitness Journal. 2014;18(2):19-22. | Ineligible study design |
| Ting A, King W, Yocum L, Antonelli D, Moynes D, Kerlan R, et al. Stress fractures of the tarsal navicular in long-distance runners. Clin Sports Med. 1988;7(1):89-101. | Ineligible study design |
| Toresdahl B, McElheny K, Fontana MA, et al. Risk factors associated with injuries in first-time marathon runners: A 12-week prospective study. Clin J Sport Med 2019;29 (2):169-70. | Ineligible outcomes |
| Toresdahl BG, McElheny K, Metzl J, Ammerman B, Chang B, Kinderknecht J. A Randomized Study of a Strength Training Program to Prevent Injuries in Runners of the New York City Marathon. Sports health. 2019:1941738119877180. | Ineligible outcomes |
| Torg JS. Athletic footwear and orthotic appliances. Clin Sports Med. 1982;1(1):157-75. | Ineligible study design |
| Toshiyuki K, Ryuichi S, Tadao I. Mechanical properties of achilles tendon in relation to various sport activities of collegiate athletes. International Symposium on Biomechanics in Sports: Conference Proceedings Archive. 2012;30:144-7. | Ineligible outcomes |
| Travers PR. Sports injuries. Physiotherapy. 1980;66(7):215-6. | Ineligible study design |
| Tsai LC, Lyle MA, Popovich JM, Jr. Re: hip strength and knee pain in high school runners: a prospective study. Pm R. 2012;4(8):634-5; author reply 5-6. | Ineligible study design |
| Turnipseed W. Popliteal entrapment in runners. Clin Sports Med. 2012;31(2):321-8. | Ineligible study design |
| Tyflidis A, Kipreos G, Tripolitsioti A, Stergioulas a. Epidemiology of track & field injuries: a one year experience in athletic schools. Biology of Sport. 2012;29(4):291-5. | Ineligible participants |
| Uitenbroek D. The mathematical relationship between the number of events in which people are injured and the number of people injured. BJSM online. 1995;29(2):126-8. | Ineligible outcomes |
| Vadeboncoeur TF, Silvers SM, Taylor WC, Shapiro SA, Roth JA, Diehl N, et al. Impact of a high body mass index on lower extremity injury in marathon/half-marathon participants J Phys Act Health. 2012;9(1):96-103. | Ineligible outcomes |
| Vahl R. Running. Some simple but often over-looked aspects. American Chiropractor. 1987:26-30. | Ineligible study design |
| van der Worp H, van der Does HTD, Brink MS, Zwerver J, Hijmans JM. Prospective Study of the Relation between Landing Biomechanics and Jumper's Knee. Int J Sports Med. 2016;37(3):245-50. | Ineligible participants |
| van Mechelen W. Can running injuries be effectively prevented? Sports Med. 1995;19(3):161-5. | Ineligible study design |
| van Mechelen W, Hlobil H, Kemper HC, Voorn WJ, de Jongh HR. Prevention of running injuries by warm-up, cool-down, and stretching exercises. Am J Sports Med. 1993;21(5):711-9. | Ineligible outcomes |
| Van Mechelen W, Hlobil H, Rep MHG, Strobos W, Kemper HCG. Running injuries and hamstring and quadriceps weakness and balance: A case-control study in male runners. Sports Medicine, Training and Rehabilitation. 1994;5(2):83-93. | Ineligible study design |
| van Middelkoop M, Cloosterman KL, Fokkema T, et al. Prevalence and impact of knee osteoarthritis in runners. Osteoarthritis and cartilage 2019;27:S258‐. | Ineligible outcomes |
| Van Middelkoop M, Kolkman J, Van Ochten J, Bierma-Zeinstra SM, Koes B. Prevalence and incidence of lower extremity injuries in male marathon runners. Scand J Med Sci Sports. 2008;18(2):140-4. | Ineligible outcomes |
| van Middelkoop M, Kolkman J, van Ochten J, Bierma-Zeinstra SM, Koes BW. Course and predicting factors of lower-extremity injuries after running a marathon. Clin J Sport Med. 2007;17(1):25-30. | Ineligible outcomes |
| Van Middelkoop M, Kolkman J, Van Ochten J, Bierma-Zeinstra SM, Koes BW. Risk factors for lower extremity injuries among male marathon runners. Scand J Med Sci Sports. 2008;18(6):691-7. | Ineligible outcomes |
| van Poppel D, de Koning J, Verhagen AP, Scholten-Peeters GG. Risk factors for lower extremity injuries among half marathon and marathon runners of the Lage Landen Marathon Eindhoven 2012: A prospective cohort study in the Netherlands. Scand J Med Sci Sports. 2016;26(2):226-34. | Ineligible outcomes |
| van Poppel D, Scholten-Peeters GG, van Middelkoop M, Verhagen AP. Prevalence, incidence and course of lower extremity injuries in runners during a 12-month follow-up period. Scand J Med Sci Sports. 2014;24(6):943-9. | Ineligible outcomes |
| van Poppel D, Scholten-Peeters GGM, van Middelkoop M, Koes BW, Verhagen AP. Risk models for lower extremity injuries among short- and long distance runners: A prospective cohort study. Musculoskeletal Science and Practice. 2018;36:48-53. | Ineligible outcomes |
| Vandeleur DM, Krabak BJ. Characterization of foot strike pattern and injury in athletes competing in a 7 day staged 200-km ultramarathon. J Investig Med. 2015;63 (1):189. | Ineligible study design |
| Vassil JC, Winn L, Heslop DJ. The Sun Herald Sydney City-2-Surf Fun Run - Historical Injury Patterns and Factors Influencing Injury Type and Frequency. Prehospital Disaster Med 2020;35(2):189-96. | Ineligible study design |
| Verhagen E. Prevention of running-related injuries in novice runners: are we running on empty? BJSM online. 2012;46(12):836-7. | Ineligible study design |
| Vernillo C, Savoldelli A, La Torre A, Skafidas S, Bortolan L, Schena F. Injury and Illness Rates During Ultratrail Running. Int J Sports Med. 2016;37(7):565-9. | Ineligible outcomes |
| Verstappen FT, Twellaar M, Hartgens F, van Mechelen W. Physical fitness and sports skills in relation to sports injuries. A four-year prospective investigation of sports injuries among physical education students. Int J Sports Med. 1998;19(8):586-91. | Ineligible participants |
| Vesterinen V, Häkkinen K, Laine T, Hynynen E, Mikkola J, Nummela A. Predictors of individual adaptation to high-volume or high-intensity endurance training in recreational endurance runners. Scand J Med Sci Sports. 2016;26(8):885-93. | Ineligible outcomes |
| Vincent HK, Chen C, Bruner ML, Wasser J, Vincent KR. Poster 32: Recent History of Lower Extremity Injury and Kinetic Characteristics of Overweight and Obese Runners. PM & R: Journal of Injury, Function & Rehabilitation. 2018;10(9):S2-S3. | Ineligible study design. |
| Vlahek P, Matijevic V. Lower extremity injuries in novice runners: Incidence, types, time patterns, sociodemographic and motivational risk factors in a prospective cohort study. Acta Clinica Croatica. 2018;57(1):31-8. | Ineligible outcomes |
| Volpe AJ, Amis JA. Evaluation and management of foot disorders in the runner. Techniques in Orthopaedics. 1990;5(3):47-56. | Ineligible study design |
| W. V R. A stitch in time or in running. Pediatrics. 2015;135(1):48-. | Ineligible study design |
| Walden M. Running injuries. Update. 2005;71(1):24-7. | Ineligible study design |
| Wallis A, Kemp J, Opar M. Athletic groin pain: Pubic origins, relationship to the hip joint, and associated risk factors. Sport Health. 2012;30(4):16-23. | Ineligible study design |
| Walter SD, Hart LE, McIntosh JM, Sutton JR. The Ontario cohort study of running-related injuries. Arch Intern Med. 1989;149(11):2561-4. | Ineligible outcomes |
| Walter SD, Hart LE, Sutton JR, McIntosh JM, Gauld M. Training habits and injury experience in distance runners: age- and sex-related factors. Physician and Sportsmedicine. 1988;16(6):101-13. | Ineligible study design |
| Wang B, Yang Y, Zhang X, et al. Twelve-Week Gait Retraining Reduced Patellofemoral Joint Stress during Running in Male Recreational Runners. Biomed Res Int 2020 | Ineligible study design |
| Wang J, Luo Z, Dai B, et al. Effects of 12-week cadence retraining on impact peak, load rates and lower extremity biomechanics in running. PeerJ 2020;8 (no pagination) | Ineligible outcomes |
| Warden S, Davis I, Fredericson M. Management and prevention of bone stress injuries in long-distance runners. Journal of Orthopaedic and Sports Physical Therapy. 2014;44(10):749-65. | Ineligible study design |
| Warne JP, Kilduff SM, Gregan BC, Nevill AM, Moran KA, Warrington GD. A 4-week instructed minimalist running transition and gait-retraining changes plantar pressure and force. Scand J Med Sci Sports. 2014;24(6):964-73. | Ineligible outcomes |
| Warren B. Plantar fasciitis in runners: treatment and prevention. Sports Medicine (Auckland). 1990;10(5):338-45. | Ineligible study design |
| Warren B, Jones C. Predicting plantar fasciitis in runners. Medicine and Science in Sports and Exercise. 1987;19(1):71-3. | Ineligible study design |
| Warren BL. Anatomical factors associated with predicting plantar fasciitis in long-distance runners. Med Sci Sports Exerc. 1984;16(1):60-3. | Ineligible study design |
| Warren BL, Davis V. Determining predictor variables for running-related pain. Phys Ther. 1988;68(5):647-51. | Ineligible study design |
| Weimer B, Saxena A, Langer P. The Clinic: Avoid Running with a Foot Drop. Running & FitNews. 2014;32(1):17-. | Ineligible study design |
| Weiss M, Miller GK, Hull L. The clinic. Can distance running cause an ACL tear? Running & FitNews. 2007;25(6):13-4. | Ineligible study design |
| Welsh RP, Clodman J. Clinical survey of Achilles tendinitis in athletes. Can Med Assoc J. 1980;122(2):193-5. | Ineligible study design |
| Wernicke AG, Panush RS. Running and the musculoskeletal system. Bull Rheum Dis. 2001;50(11):1-4. | Ineligible study design |
| Westphal K. Foot-injury in joggers: Deformities, stress fractures and wrong footwear. [German]. MMW-Fortschritte der Medizin. 2003;145(31-32):4-8. | Ineligible study design |
| Wezenbeek E, Willems T, Mahieu N, De Muynck M, Vanden Bossche L, Steyaert A, et al. The Role of the Vascular and Structural Response to Activity in the Development of Achilles Tendinopathy: A Prospective Study. Am J Sports Med. 2018;46(4):947-54. | Ineligible participants |
| Wiechman SA, Williams J. Relation of athletic identity to injury and mood disturbance. Journal of Sport Behavior. 1997;20(2):199. | Ineligible participants |
| Wilder RP. Preface: The runner. Clin Sports Med. 2010;29(3):xv-xvi. | Ineligible study design |
| Wilder RP, Magrum E. Exertional compartment syndrome. Clin Sports Med. 2010;29(3):429-35. | Ineligible study design |
| Wilk BR. Can I run on this injury? Running & FitNews. 2009;27(4):16-21. | Ineligible study design |
| Wilk BR. Avoiding the abyss: when runners should seek professional help. Running & FitNews. 2009;27(3):12-4. | Ineligible study design |
| Wilk BR, Garis A, Johnson C, Cheung RTH, Davis IS. Foot strike pattern in runners. J Orthop Sports Phys Ther. 2012;42(2):147-8. | Ineligible study design |
| Wilk BR, Nau S. The Effects of Arm Swing on Running Mechanics. Running & FitNews. 2013;31(1):4-7. | Ineligible study design |
| Willems T, De CD, Delbaere K, Vanderstraeten G, De CA, Witvrouw E. A prospective study of gait related risk factors for exercise-related lower leg pain. Gait and Posture. 2006;23(1):91-8. | Ineligible participants |
| Willems T, Witvrouw E, De CA, De CD. Gait-Related Risk Factors for Exercise-Related Lower-Leg Pain during Shod Running. Medicine and Science in Sports and Exercise. 2007;39(2):330-9. | Ineligible participants |
| Willems T, Witvrouw E, Delbaere K, De Cock A, De Clercq D. Relationship between gait biomechanics and inversion sprains: a prospective study of risk factors. Gait Posture. 2005;21(4):379-87. | Ineligible participants |
| Willems TM, Witvrouw E, Delbaere K, Mahieu N, Bourdeaudhuij I, De Clercq D. Intrinsic risk factors for inversion ankle sprains in male subjects: a prospective study. Am J Sports Med. 2005;33(3):415-23. | Ineligible participants |
| Willems TM, De Ridder R, Roosen P. Is consumer behaviour towards footwear predisposing for lower extremity injuries in runners and walkers? A prospective study. J 2019;12:43. | Ineligible outcomes |
| Williams D, McClay I, Hamill J. Arch structure and injury patterns in runners. Clin Biomech. 2001;16(4):341-7. | Ineligible study design |
| Williams DS, III. Lower extremity mechanics and injury patterns in runners with pes cavus and pes planus: University of Delaware; 2000. | Ineligible study design |
| Williams DSB, III. Rearfoot and lower leg alignment and mechanics in the knee-injured population. Sports Physical Therapy Section. 2002:16-7. | Ineligible study design |
| Williams DSB, Tierney RN, Butler RJ. Increased Medial Longitudinal Arch Mobility, Lower Extremity Kinematics, and Ground Reaction Forces in High-Arched Runners. Journal of Athletic Training (Allen Press). 2014;49(3):290-6. | Ineligible study design. |
| Willson J, Ratcliff O, Meardon S, Willy R. Influence of step length and landing pattern on patellofemoral joint kinetics during running. Scandinavian Journal of Medicine and Science in Sports. 2015;25(6):736-43. | Ineligible study design |
| Willson JD, Bjorhus JS, Williams 3rd DSB, Butler RJ, Porcari JP, Kernozek TW. Short-term changes in running mechanics and foot strike pattern after introduction to minimalistic footwear. PM & R: Journal of Injury, Function & Rehabilitation. 2014;6(1):34-43. | Ineligible outcomes |
| Willy RW, Buchenic L, Rogacki K, Ackerman J, Schmidt A, Willson JD. In-field gait retraining and mobile monitoring to address running biomechanics associated with tibial stress fracture. Scand J Med Sci Sports. 2016;26(2):197-205. | Ineligible outcomes |
| Willy RW, Davis IS. The effects of a hip strengthening program on running and squatting kinematics in females at risk for patellofemoral pain syndrome...2010 Combined Sections Meeting (CSM), San Diego, California, February 17-20, 2010. J Orthop Sports Phys Ther. 2010;40(1):A50-A. | Ineligible outcomes |
| Willy RW, Davis IS. The effect of a hip-strengthening program on mechanics during running and during a single-leg squat. J Orthop Sports Phys Ther. 2011;41(9):625-32. | Ineligible outcomes |
| Winter DA, Bishop PJ. Lower extremity injury. Biomechanical factors associated with chronic injury to the lower extremity. Sports Med. 1992;14(3):149-56. | Ineligible study design. |
| Winter SC, Gordon S, Brice SM, et al. A Multifactorial Approach to Overuse Running Injuries: A 1-Year Prospective Study. Sports health 2020;12(3):296-303 | Ineligible study design (secondary analysis of included study) |
| Witvrouw E, Van Tiggelen D, Thijs Y. Intrinsic risk factors for patellofemoral pain syndrome: Implications for prevention and treatment. Journal of Science and Medicine in Sport. 2011;14:e118. | Missing information |
| Woolf SK, Barfield WR, Nietert PJ, Mainous 3rd AG, Glaser JA. The Cooper River Bridge Run Study of low back pain in runners and walkers. J South Orthop Assoc. 2002;11(3):136-43. | Ineligible study design. |
| Wouters I, Almonroeder T, Dejarlais B, Laack A, Willson JD, Kernozek TW. Effects of a movement training program on hip and knee joint frontal plane running mechanics. International Journal of Sports Physical Therapy. 2012;7(6):637-46. | Ineligible outcomes |
| Wyndow N, Cowan S, Wrigley T, Crossley K. Triceps surae activation is altered in male runners with Achilles tendinopathy. Journal of Electromyography and Kinesiology. 2013;23(1):166-72. | Ineligible study design. |
| Yagi S, Muneta T, Sekiya I. Incidence and risk factors for medial tibial stress syndrome and tibial stress fracture in high school runners. Knee Surg Sports Traumatol Arthrosc. 2013;21(3):556-63. | Ineligible participants |
| Yamamoto T. Relationship between hamstring strains and leg muscle strength. A follow-up study of collegiate track and field athletes. / Relations entre les elongations des muscles de la loge posterieure de la cuisse et la force des membres inferieurs. Etude chez des athletes universitaires pratiquant l ' athletisme. J Sports Med Phys Fitness. 1993;33(2):194-9. | Ineligible participants |
| Yeung SS, Suen AM, Yeung EW. A prospective cohort study of hamstring injuries in competitive sprinters: preseason muscle imbalance as a possible risk factor. BJSM online. 2009;43(8):589-94. | Ineligible participants |
| Zhang GQ, Meng ML, Su GY, Liu YF, Zhou N. Survey and analysis of injuries due to track and field sports among college students majoring in physical education in Shanxi province. [Chinese]. Chinese Journal of Clinical Rehabilitation. 2005;9(44):118-20. | Ineligible study design |
| Zifchock RA, Davis I, Higginson J, McCaw S, Royer T. Side-to-side differences in overuse running injury susceptibility: a retrospective study. Hum Mov Sci. 2008;27(6):888-902. | Ineligible study design. |
