# Supplementary material for: Biomechanical and Musculoskeletal Measurements as Risk Factors for Running-Related Injury in Non-elite Runners: A Systematic Review and Meta-analysis of Prospective Studies
Source: Sports Med Open. 2022 Mar 7;8:38. doi: 10.1186/s40798-022-00416-z (PMC8901814; doi:10.1186/s40798-022-00416-z)
Supplement: Supplementary file 2 — Additional file 2. Risk factors for RRI – Results from individual studies. [file 40798_2022_416_MOESM2_ESM.docx]

| **Additional table 1. Results of individual trials – Results relate to injured vs. non-injured unless otherwise stated** | | | |
| --- | --- | --- | --- |
| Study ID and Outcome | Risk factor | Measurement technique | Result |
| Bennett et al. 2012  ERLLP | Navicular drop | Navicular tuberosity height from NCSP to RCSP | Navicular drop >10mm medial ERLLP vs no ERLLP OR 6.6 (1.2-38) p=0.03  Navicular drop >10mm ERLLP vs no ERLLP OR 0.9 (0.2 – 2.6) |
|  | Plantar flexor endurance | n standing heel raises (rate of one heel raise/second | Non-significant |
| Bring et al. 2018  RRI  (Data re-analysed for this review) | Functional movement screen | Functional movement screen score (out of 21) | Incidence of injury and total number of injuries not associated with any individual domain, or with total score.  Cut point >14:Injured vs. non-injured Pearson Chi2 p=0.142. Number of injuries independent samples t-test 0.16 (p=0.045)  Cut point >15:Injured vs. non-injured Pearson Chi2 p=0.142.Number of injuries independent samples t-test 0.16 (p=0.083) |
| Buist et al. 2010  RRI | Hip joint internal rotation ROM | Passive hip joint internal rotation ROM (hip flexed). Twice per leg then averaged. | HR male: 1.00 p=0.63. HR female: 0.98 p=0.08 |
|  | Hip joint external rotation ROM | Passive hip joint external rotation ROM (hip flexed). Twice per leg then averaged. | HR male: 1.01 (p=0.28). HR female 1.00 (p=0.86) |
|  | Ankle joint dorsiflexion ROM | Passive ankle joint dorsiflexion ROM (knee flexed). Twice per foot then averaged. | HR male: 1.01 (p=0.42). HR female: 1.00 (p=0.61) |
|  |  | Passive ankle joint dorsiflexion ROM (knee extended). Twice per foot then averaged. | HR male: 1.01 (p=0.45). HR female: 1.00 (p=0.87) |
|  | Navicular drop | Navicular tuberosity height from seated STJ neutral to standing RCSP. Twice per foot then averaged. | HR male: 1.02 (p=0.40) HR female: 0.92 (p=0.01)* |
| Davis et al. 2003  Patellofemoral pain syndrome | Pk eversion (calc) | Subjects run along a 25 m runway at a speed of 3.65 m/s (±5%). Kinematic data collected (120 Hz) with a 6-camera Vicon Motion Systems. All kinematic data are filtered at 8 Hz. 3D angles of interest are calculated about a joint coordinate system using MOVE3D. Five trials were averaged for analysis. | p=0.5 |
|  | Knee adduction |  | p=0.28 |
|  | Knee internal rotation |  | p=0.47 |
|  | Hip adduction |  | p=0.1 |
|  | Hip external rotation |  | p=0.08 |
|  | Q-angle | Method not reported. | p=0.05 |
| Davis et al. 2016  RRI and medically diagnosed injuries | Peak vertical force, BW | Participants wore standard, neutral running shoes during baseline screening and all subsequent data collections. They ran at 3.7 ms (±5%) across a force plate located in the centre of a 25 m runway. Ground reaction force data were sampled at 1080 Hz and filtered at a cut-off frequency of 50 Hz using a second order, recursive, Butterworth, low pass filter. | Non-injured vs. Injured: p=0.72  Never injured vs medically diagnosed injury: p= 0.954 |
|  | Vertical average load rate, BW/s |  | Non-injured vs. Injured: p=0.357. Never injured vs medically diagnosed injury: p=0.001 |
|  | Vertical instantaneous load rate, BW/s |  | Non-injured vs. Injured: p=0.14. Never injured vs medically diagnosed injury: p=0.014 |
|  | Vertical impact peak BW |  | Non-injured vs. Injured: p=0.88. Never injured vs medically diagnosed injury: p=0.013 |
| Desai et al. 2021 RRI | Coordinative variability-initial stance  Knee-ankle | Participants at a speed of 4m/s (+/- 5%) across an 18m level runway. Data were sampled at 240Hz by a 9 camera motion capture system (Oqus 400+, Qualysis AB, Gotherburg, Sweeden). Gait speed was measured by two infared break beam sensors (TC-Gait, Brower Timing System, Draper, UT, USA) placed 6m apart within the centre of the data collection volume. Raw kinematic data were filtered using a bi-directional, 4th order, Butterworth lowpass filter with zero lag and a cut-off frequency of 12 Hz.  Stance phase was time normalized from initial contact (0%) to toe-off (100%). Stance was divided into three portions: initial-stance (0–33%), mid-stance (34–66%), and late-stance  (67–100%) to compare coordinative patterns and CAV within each portion between groups. CAV was quantified at the knee, shank, and ankle using a continuous circular analysis measure known as vector coding. | Consensus RRI definition: <0.05  Authors RRI definition: <0.05 |
|  | Coordinative variability-initial stance  Shank-ankle |  | Consensus RRI definition: NS  Authors RRI definition: NS |
|  | Coordinative variability-initial stance  Knee-shank |  | Consensus RRI definition: <0.05  Authors RRI definition: <0.05 |
|  | Coordinative variability-mid-stance  Knee-ankle |  | Consensus RRI definition: NS  Authors RRI definition: NS |
|  | Coordinative variability-mid-stance  Shank-ankle |  | Consensus RRI definition: NS  Authors RRI definition: NS |
|  | Coordinative variability-mid-stance  Knee-shank |  | Consensus RRI definition: <0.05  Authors RRI definition: NS |
|  | Coordinative variability-late-stance  Knee-ankle |  | Consensus RRI definition: NS  Authors RRI definition: NS |
|  | Coordinative variability-late-stance  Shank-ankle |  | Consensus RRI definition: <0.05  Authors RRI definition: <0.05 |
|  | Coordinative variability-late-stance  Knee-shank |  | Consensus RRI definition: NS  Authors RRI definition: NS |
|  | Peak ankle eversion | Participants at a speed of 4m/s (+/- 5%) across an 18m level runway. Data were sampled at 240Hz by a 9 camera motion capture system (Oqus 400+, Qualysis AB, Gotherburg, Sweeden). Gait speed was measured by two infared break beam sensors (TC-Gait, Brower Timing System, Draper, UT, USA) placed 6m apart within the centre of the data collection volume. Raw kinematic data were filtered using a bi-directional, 4th order, Butterworth lowpass filter with zero lag and a cut-off frequency of 12 Hz. | p=0.28 |
|  | Peak ankle inversion |  | p=0.25 |
|  | Peak shank internal rotation |  | p=0.44 |
|  | Peak shank external rotation |  | p=0.79 |
|  | Peak knee flexion |  | p=0.66 |
|  | Average ankle motion |  | p<0.01 |
|  | Average shank motion |  | p<0.01 |
|  | Average knee motion |  | p=0.8 |
| Hamill et al. 2007  Iliotibial band syndrome/strain | Strain (%) at touchdown | Strain consisted of the simulated length at each time step during the stance phase of the running stride | p=0.26, ES=0.23 |
|  | Strain (%) at max knee flexion | Strain consisted of the simulated length at each time step during the stance phase of the running stride | p=0.11; ES=0.45 |
|  | Strain rate (%/sec) | Change in strain from touchdown to maximum knee flexion divided by time from touchdown to maximum knee flexion | p=0.001; ES=1.91 |
|  | Duration of impingement (%) | Impingement between ITB and lateral femoral epicondyle (LFE) was modelled by defining a wrapping sphere whose surface was flush against the outer surface of the LFE. Duration of impingement was defined as the range of knee flexion angles during which the ITB interacted with the LFE. | p=0.11; ES=0.44 |
| Hein et al. 2014  Achilles tendon pain | Abdominal Flexion Strength | MVIC measured fixated in a seated with 90^o^ of knee flexion, without using hands for stabilisation, with the upper body in an upright position (0^o^) | p-values not reported in study |
|  | Back extension strength | MVIC measured fixated in a seated with 90^o^ of knee flexion, without using hands for stabilisation, with the upper body at a 30^o^ forward incline |  |
|  | Bilateral hip abduction strength | MVIC measured standing with 20^o^ of hip abduction, using a ‘hip machine’ |  |
|  | Bilateral hip adduction strength | MVIC measured standing with 20^o^ of hip abduction, using a ‘hip machine’ |  |
|  | Unilateral knee flexion strength | MVIC measured seated with 30 degrees of knee flexion, using a DAVID device’ |  |
|  | Unilateral knee extension strength | MVIC measured seated with 60 degrees of knee extension, using a DAVID device’ |  |
|  | Lower limb kinematics during running task | Maximum knee flexion (and its timing), maximum ankle dorsiflexion (and its timing), ankle joint dorsiflexion ROM, ankle joint plantarflexion ROM, maximum rearfoot eversion, rearfoot eversion ROM), maximum rearfoot inversion, rearfoot inversion ROM. |  |
| Hendricks et al. 2013  RRI  (Data re-analysed for this review) | Leg length difference | Participant lying in supine. Distance from ASIS to medial malleolus measured using measuring tape. | MD -0.53 (-1.07, 0.01) (p=0.05) |
|  | Q-angle | Participant in bipedal stance. ASIS marked, and fulcum of goniometer positioned at the patella, one arm oriented toward the ASIS, and the other toward the tibial tuberosity. | MD -1.80 (-6.27, 2.67) (p=0.43) |
|  | Hip flexion ROM | Procedure unclear. Measured using universal goniometer. Three trials averaged. | MD -2.64 (-12.33, 7.05) (p=0.59) |
|  | Hip extension ROM |  | MD 0.75 (-5.77, 7.27) (p=0.82) |
|  | Hip abduction ROM |  | MD -2.78 (-11.02, 5.46) (p=0.51) |
|  | Hip adduction ROM |  | MD -0.33 (-8.32, 7.66) (p=0.94) |
|  | Hip internal rotation ROM |  | MD -3.60 (-10.71, 3.51) (p=0.32) |
|  | Hip external rotation ROM |  | MD -1.25 (-10.20, 7.70) (p=0.78) |
|  | Knee flexion ROM |  | MD -4.66 (-10.83, 1.51) (p=0.14) |
|  | Muscle strength | 1RM leg-press on a leg-press machine. Weight pressed divided by body mass for leg-press to weight ratio. Documented as percentile rankings (e.g. >70 = above average, 50 = average, <30 = below average). | Missing data precluded re-analysis |
| Hesar et al. 2009  Lower leg overuse injury |  | Participants ran barefoot at a comfortable self selected speed over a force plate on a 15metre runway. 3 bilateral steps were analysed for kinetic plantar pressure variables. |  |
|  | Laterally directed force distribution at first metatarsal contact ratio 1 |  | Adjusted OR 0.63 (0.42-0.96, p=0.032) |
|  | Laterally directed force distribution at first metatarsal contact ratio 2 |  | Adjusted OR= 0.65 (0.43-0.98, p=0.038) |
|  | Laterally directed force distribution at first metatarsal contact ratio 3 |  | Adjusted OR= 0.64 (0.42-0.97, p=0.035) |
|  | Laterally directed force distribution at first metatarsal contact ratio 8 |  | Adjusted OR= 0.58 (0.38-0.90, p=0.016) |
|  | Laterally directed force distribution at forefoot flat ratio 1 |  | Adjusted OR= 0.65 (0.41-1.01, p=0.057) |
|  | Laterally directed force distribution at forefoot flat ratio 3 |  | Adjusted OR= 0.63 (0.4-0.99, p=0.047 |
|  | Displacement from medial to lateral in initial contact phase ratio 8 |  | Adjusted OR= 0.59 (0.36-0.96, p=0.033) |
|  | Laterally directed center of force during heel-off |  | Adjusted OR= 0.74 (0.61-0.90, p=0.002) |
|  | Laterally directed center of force during forefoot contact phase |  | Adjusted OR= 0.4 (0.22-0.73, p=0.003) |
|  | Laterally directed center of force during foot flat phase |  | Adjusted OR= 0.59 (0.38-0.92, p=0.021) |
|  | Medially directed center of force during forefoot push off phase |  | Adjusted OR= 1.67 (1.08-2.57, p=0.02) |
|  | Velocity of mediolateral displacement of center of force at forefoot flat |  | Adjusted OR= 0.51 (0.27-0.95, p=0.033) |
|  | Velocity of anteroposterior displacement of centre of force at forefoot flat |  | Adjusted OR= 0.65 (0.42-1.01, p=0.055) |
|  | Anteroposterior displacement of center of force at forefoot flat |  | Adjusted OR= 1.75 (1.09-2.80, p=0.021) |
|  | Absolute force-time integral underneath metatarsal 5 |  | Adjusted OR= 1.72 (1.13-2.64, p=0.021) |
|  | Vertical plantar peak force underneath metatarsal 5 |  | Adjusted OR 1.68 (1.09-2.60, p=0.019) |
| Hespanhol Jr. (2016)  RRI | Leg length difference | Participant lying in supine. Distance from the ASIS to the centre of the ipsilateral medial malleolus was measured. | OR=1.3 (0.6 to 2.7) |
|  | Q-angle | Participant in bipedal stance. ASIS marked, and fulcum of goniometer positioned at the patella, one arm oriented toward the ASIS, and the other toward the tibial tuberosity. Values between 10° and 15° were considered normal for both genders. | OR=0.9 (0.8 to 1.0) |
|  | Subtalar angle | Measured relative to vertical. 0-5^o^ = normal, <0^o^ = varus, >5^o^ valgus subtalar angle. | OR=1.0 (0.8 to 1.2) |
|  | Plantar arch index | Participant stood on a podoscope and their footprint photographed.  Foot was divided into three areas: forefoot, midfoot, and rearfoot. The area of the midfoot was divided by the area of the three regions combined to calculate the plantar arch index. | Normal arch: OR=1  High arch: OR=1.0 (0.3 to 3.1)  Low arch: OR=1.0 (0.3 to 3.8) |
| Hotta et al. 2015  RRI | Functional movement screen (FMS) | Each movement test was scored on a 4-point scale (0-3), and the maximal FMS score that could be achieved was 21. A score of 3 was awarded for perfect form, a score of 2 for completing the test with compensations, score of 1 for not completing the test accurately, and a score of 0 if the subjects felt any pain during the test. Each test was performed 3 times, and the highest score was used. Of the 7 tests that comprise the FMS, 5 tests (HS, ILL, SM, ASLR, and RS) were performed and scored separately for the right and left sides of the body. For these bilaterally assessed tests, the lower score was used. |  |
|  | *FMS total score* |  | No serious injury vs serious injury p=0.1 |
|  | *deep squat* |  | No serious injury vs serious injury p=0.01 |
|  | *hurdle step* |  | No serious injury vs serious injury p= 0.2 |
|  | *in-line lunge* |  | No serious injury vs serious injury p=0.26 |
|  | *shoulder mobility* |  | No serious injury vs serious injury p=0.36 |
|  | *activie straight leg raise* |  | No serious injury vs serious injury p<0.01 |
|  | trunk stability push-up |  | No serious injury vs serious injury p=0.06 |
|  | rotary stability |  | No serious injury vs serious injury p=0.97 |
|  | FMS <14 | Multivariate adjusted for age, height, weight, running experience, weekly training sessions, weekly mileage, performance level, and injury history | Univariate OR: 3.2 (0.9-11, p=0.07)  Multivariate OR: 3 (0.8-11.6, p=0.1) |
|  | DS and ASLR <3 |  | Injury vs. no injury during follow-up  Univariate OR: 7.8 (2.2-27.6, p<0.01)  Multivariate OR: 9.7 (2.1-44.4, p<0.01) |
| Jungmalm et al. 2020 RRI | Hip adduction ROM (degrees) | Movement variables were collected using a 16-camera motion capture system (Qualisys AB, Gothenburg, Sweden) with a sampling frequency of 400Hz, while participants ran overground at a controlled speed of 3.33m/s (+/- 5%) with a set of reflective markers applied according to ISB recommendations. | 1SD below reference: p=0.588  1SD above reference: p=0.095 |
|  | Hip adduction velocity (deg/sec) |  | 1SD below reference: p=0.87  1SD above reference: p=0.094 |
|  | Hip adduction timing (%) |  | 1SD below reference: p=0.78  1SD above reference: p=0.624 |
|  | Knee flexion ROM (deg) |  | 1SD below reference: p=0.796  1SD above reference: p=0.401 |
|  | Max excursion knee Flexion (deg) |  | 1SD below reference: p=0.541  1SD above reference: p=0.236 |
|  | Knee flexion velocity (deg/sec) |  | 1SD below reference: p=0.783  1SD above reference: p=0.675 |
|  | Knee flexion timing (%) |  | 1SD below reference: p=0.14  1SD above reference: p=0.702 |
|  | Rearfoot eversion ROM (deg) |  | 1SD below reference: p=0.272  1SD above reference: p=0.93 |
|  | Rearfoot eversion velocity (deg/sec) |  | 1SD below reference: p=0.981  1SD above reference: p=0.487 |
|  | Rearfoot eversion timing (%) |  | 1SD below reference: p=0.786  1SD above reference: p=0.033 |
|  | Ankle dorsiflexion ROM (deg) |  | 1SD below reference: p=0.61  1SD above reference: p=0.193 |
|  | Ankle touchdown angle (deg) |  | 1SD below reference: p=0.65  1SD above reference: p=0.186 |
|  | Ankle dorsiflexion timing (%) |  | 1SD below reference: p=0.115  1SD above reference: p=0.244 |
|  | Hip abduction strength | Strength variables were isometric maximal voluntary strength measures (MVC) and ratios between agonistic and antagonistic muscle groups. Strength was measured using DAVID devices (David Health Solutions, Helsinki, Finland) and the maximal torque (Nm) for each test was normalised to body weight (Nm/kg). | 1SD below reference: p=0.793  1SD above reference: p= 0.332 |
|  | Hip adduction strength |  | 1SD below reference: p=0.634  1SD above reference: p= 0.328 |
|  | Knee extension strength |  | 1SD below reference: p=0.065  1SD above reference: p= 0.59 |
|  | Knee flexion strength |  | 1SD below reference: p=0.741  1SD above reference: p= 0.645 |
|  | Trunk rotation strength |  | 1SD below reference: p=0.065  1SD above reference: p= 0.6 |
|  | Trunk flexion strength |  | 1SD below reference: p=0.608  1SD above reference: p= 0.118 |
|  | Trunk extension strength |  | 1SD below reference: p=0.925  1SD above reference: p= 0.678 |
|  | Hip abduction:adduction strength ratio |  | 1SD below reference: p=0.04  1SD above reference: p= 0.774 |
|  | Hamstring:quadriceps strength ratio |  | 1SD below reference: p=0.666  1SD above reference: p= 0.156 |
|  | Trunk flexion:extension strength ratio |  | 1SD below reference: p=0.436  1SD above reference: p= 0.82 |
|  | Hip flexion ROM | Joint ROM was measured according to the neutral-zero method and classified as 'reference', 'hypermobile', or 'hypomobile' | Hypermobile: p= N/A  Hypomobile: p= 0.286 |
|  | Hip extension ROM |  | Hypermobile: p= 0.569  Hypomobile: p= N/A |
|  | Hip abduction ROM |  | Hypermobile: p= 0.822  Hypomobile: p= 0.539 |
|  | Hip adduction ROM |  | Hypermobile: p= N/A  Hypomobile: p= N/A |
|  | Hip internal rotation ROM |  | Hypermobile: p= N/A  Hypomobile: p= N/A |
|  | Hip external rotation ROM |  | Hypermobile: p= 0.855  Hypomobile: p= 0.166 |
|  | Knee flexion ROM |  | Hypermobile: p= 0.896  Hypomobile: p= 0.724 |
|  | Knee extension ROM |  | Hypermobile: p= 0.16  Hypomobile: p= 0.68 |
|  | Ankle dorsiflexion ROM |  | Hypermobile: p= N/A  Hypomobile: p= 0.682 |
|  | Ankle plantarflexion ROM |  | Hypermobile: p= N/A  Hypomobile: p= N/A |
|  | Ankle pronation ROM |  | Hypermobile: p= 0.525  Hypomobile: p= 0.61 |
|  | Ankle supination ROM |  | Hypermobile: p= 0.549  Hypomobile: p= 0.73 |
|  | Rectus femoris flexibility | Muscle flexibility was assessed unilaterally for the hip flexors (rectus femoris and iliopsoas) with the Thomas test, and for the hamstrings with the straight leg raise test. | p=0.845 |
|  | Iliopsoas flexibility |  | p=0.465 |
|  | Hamstring flexibility |  | p=0.992 |
|  | ITB trigger points | Trigger points were defined as a tender area in a muscle that reproduces pain during palpation. Participants informed the examiner whether the applied pressure at the different locations was accompanied by pain or not. | p=0.996 |
|  | Gastrocnemius trigger points |  | p=0.288 |
|  | Soleus trigger points |  | p=0.109 |
|  | Piriformis trigger points |  | p=0.743 |
|  | Glut med trigger points |  | p=0.549 |
|  | Tibialis posterior trigger points |  | p=0.604 |
|  | Tibialis anterior trigger points |  | p=0.167 |
| Leetun et al. 2004  Lower extremity injury  (Data re-analysed for this review) | Hip abduction strength, % bodyweight | Unilateral; three 5s MVIC measured side-lying with approx. 10^o^ of hip abduction. Dynamometer positioned 5cm proximal to lateral knee joint line. Resisted by a rigid strap. | MD: -1.59 (-3.38-0.20, p=0.08)** |
|  | Hip external rotation strength, % bodyweight | Unilateral; three 5s MVIC measured seated at 90^o^ hip and knee flexion. Dynamometer positioned 5cm proximal to medial malleolus. Resisted by a rigid strap | MD: 0.09 (-1.46-1.65, p=0.90)** |
|  | Lateral core strength | Side bridge, measured in seconds | MD: -0.86 (-2.49-0.76, p=0.30)** |
|  | Back extensor strenth, seconds | Modified Biering-Sorensen test | MD: -1.71 (-3.54-0.11, p=0.07)** |
| Lun et al. 2004 (13) RRI | Hip internal rotation ROM | Active ROM. Measured seated with participants feet hanging over the examination table. Inclinometer positioned against distal tibia. | Comparative statistics not reported |
|  | Hip external rotation ROM | Active ROM. Measured seated with participants feet hanging over the examination table. Inclinometer positioned against distal tibia. | Comparative statistics not reported |
|  | Knee recurvatum | Standing in maximal knee extension. Goniometer used to measure long axis of femur relative to long axis of tibia. | Comparative statistics not reported |
|  | Q-angle | ASIS marked, and fulcum of goniometer positioned at the patella, one arm oriented toward the ASIS, and the other toward the tibial tuberosity. | Comparative statistics not reported |
|  | ankle dorsiflexion ROM | Passive ROM. Goniometer positioned against lateral malleolus and lateral border of foot. Ankle joint was maximally dorsiflexed from a neutral (90^o^) start point. | Comparative statistics not reported |
|  | Ankle plantarflexion ROM | Passive ROM. Goniometer positioned against lateral malleolus and lateral border of foot. Ankle joint was maximally plantarflexed from a neutral (90^o^) start point. | Comparative statistics not reported |
|  | STJ valgus | Subject in prone, measured in degrees. | Comparative statistics not reported |
|  | STJ varus | Subject in prone, measured in degrees. | Comparative statistics not reported |
|  | Forefoot valgus | Subject in prone, measured in degrees. | Comparative statistics not reported |
|  | Forefoot varus | Subject in prone, measured in degrees. | Comparative statistics not reported |
|  | Ankle joint pronation | Bipedal stance. Subjective assessment. | Comparative statistics not reported |
|  | Medial longitudinal arch height | Bipedal stance. Subjective assessment. | Comparative statistics not reported |
|  | Leg length | Measured but not reported | Comparative statistics not reported |
|  | Genu varum | Measured but not reported | Comparative statistics not reported |
| Messier et al. 2018  RRI | Q-angle | Bipedal stance, feet standardized by a template, markers placed on the anterior superior iliac spine, midpoint of the patella, and tibial tuberosity. Measured as angle formed by the intersection of 2 lines that connected the midpoint of the patella to the anterior superior iliac spine superiorly and to the tibial tuberosity inferiorly. | p=0.14 |
|  | Arch index | A footprint, created by participant stepping onto a Harris mat with one-half of total bodyweight, was divided into thirds, not including the toes. The area of the middle third was divided by the total area to yield an arch index. | p=0.85 |
|  | Quadricep flex | Maximum knee flexion. Participant lay prone; both thighs were strapped to the examining table; and the knee was actively flexed until it met resistance or the hip began to flex. | p=0.86 |
|  | Hamstring flex | Maximum knee extension: the participant was supine; the hip and knee were flexed to 90; and a strap was placed over the contralateral thigh to secure the participant to the table | p=0.35 |
|  | Ankle flexibility | Passive ROM. Measured with the participant’s legs extended and the foot placed in a neutral angle equivalent to 90 of dorsiflexion. | p=0.6 |
|  | Ankle dorsiflexion and plantarflexion ROM | Active ROM. Plantar flexion and dorsiflexion active range of motion were calculated as change from the neutral angle. | p=0.95 |
|  | Hip abductor strength | Isokinetic strength testing. Measured through a joint arc from 0 to 30 (0 = anatomic position) with the dynamometer set at an angular velocity of 30 deg/ sec. The first and last 5 were deleted to account for the dynamometer’s acceleration and deceleration at the ends of the range of motion. | p=0.74 (adjusted: sex) |
|  | Knee extensor strength | Participant secured with torso and test leg strapped to chair. Hands across the chest. The dynamometer axis aligned with the knee, and the resistance pad attached to the lower leg proximal to ankle joint. Knee extensors and flexors were tested through a joint arc from 90 to 30 (0 = full extension). The first and last 10 were deleted to account for the dynamometer’s acceleration and deceleration at the ends of the range of motion and for possible inconsistent effort. Hence, average force was calculated between joint angles of 40 to 80. | p=0.63 (adjusted: sex) |
|  | Knee flexor strength |  | p=0.61 (adjusted: sex) |
|  | Knee flexion/extension strength ratio | Ratio of knee flexion and extension isokinetic strength. | p=0.92 (adjusted: sex and BW) |
|  | Ankle plantar flexor strength | Ankle plantar flexors and dorsiflexors were tested through a joint arc from 15 of dorsiflexion to 15 of plantar flexion. The first and last 5 were deleted to account for the dynamometer’s acceleration and deceleration at the ends of the range of motion. | p=0.6 (adjusted: sex) |
|  | Vertical impact peak, N | 3D kinematic and kinetic data were collected using a 6-camera motion capture system set to sample data at 200 Hz was synchronized with a strain gauge force platform set to sample data at 480 Hz.  37 reflective markers arranged in the Cleveland Clinic full-body configuration, plus markers on the rearfoot and the shank. Participants wore own footwear and ran at their own training pace (+3.5%) on a 22.5m runway. | p=0.74 (adjusted training pace and BW) |
|  | Vertical propulsive epeak, N |  | p=0.06 (adjusted training pace and BW) |
|  | Braking force, N |  | p=0.79 (adjusted training pace and BW) |
|  | Propulsive force, N |  | p=0.38 (adjusted training pace and BW) |
|  | Tibial compressive force, N |  | p=0.68 (adjusted training pace and BW) |
|  | Patellofemoral compressive force, N |  | p=0.75 (adjusted training pace and BW) |
|  | Knee abduction moment, NM |  | p=0.51 (adjusted training pace and BW) |
|  | Knee extension moment, NM |  | p=0.88 (adjusted training pace and BW) |
|  | Max knee flex, deg |  | p=0.82 (adjusted training pace and BW) |
|  | Knee stiffness, NM/deg |  | p=0.03 (adjusted training pace and BW) |
|  | Knee power absorption, W |  | p=0.63 (adjusted training pace and BW) |
|  | Knee negative work stance, J |  | p=0.61 (adjusted training pace and BW) |
|  | Touchdown angle, deg |  | p=0.4 |
|  | Maximum eversion, deg |  | p=0.94 |
|  | Eversion range of motion, deg |  | p=0.35 |
|  | Maximum eversion velocity, deg/s |  | p=0.59 |
|  | Forefoot angle, deg (+, adduction) |  | p=0.38 |
|  | Strike index, % (distance from heel) |  | p=0.44 |
|  | Maximum vertical GRF, N |  | OR: 0.998 (0.996-1.001, p= 0.17) |
|  | Maximum propelling force, N |  | OR: 0.998 (0.987-1.009, p= 0.72) |
|  | Maximum knee stiffness, NM/deg | ratio of the maximum change in the internal knee extension moment with the maximum change in the knee flexion angle during the first half of the support phase and in which these maxima occurred within 10% of one another. | OR: 1.184 (1.021-1.374, p=0.03) |
| Napier et al. 2018  RRI | Vertical impact transient (BW) | Running on instrumented treadmill, in self-selected footwear, and at self-selected running pace (RPE 13).  Three-dimensional kinematic data were sampled at 240 Hz using a six-camera passive marker system while three-dimensional kinetic data were sampled at 2400 Hz. A single continuous running bout was performed, and data were collected from 3 consecutive captures of 15 seconds duration each. | Not significant |
|  | Average vertical loading rate (BW/s) |  | Not significant |
|  | Instantaneous vertical loading rate (BW/s) |  | Not significant |
|  | Active peak (BW) |  | Not significant |
|  | Peak braking force (BW) |  | Peak braking force significantly associated with higher injury hazard ratio. P<0.01 |
|  | Vertical impulse (BW*s) |  | Not significant |
| Noehren et al. 2007  ITBS | Hip adduction peak (degrees) | Subjects ran along a 25 m run way, in a standard neutral running shoe, at a speed of 3.7 m/s (±5%) striking a force plate at its center.  Kinematic data were collected at 120 Hz with a 6 camera Vicon 512 motion analysis) and low-pass filtered at 8 Hz with a fourth-order zero lag Butterworth filter. Force data was sampled at 1080 Hz and low-pass filtered at 50 Hz with a fourth-order zero lag. | p=0.01 |
|  | hip abduction moment (Nm) |  | p=0.56 |
|  | Knee internal rotation moment (Nm) |  | p=0.01 |
|  | Knee external rotation moment (Nm) |  | p=0.42 |
|  | Rearfoot eversion peak (deg) |  | p=0.07 |
|  | Rearfoot inversion moment (Nm) |  | p=0.66 |
|  | Tibia in lab peak (deg) |  | p=0.23 |
|  | Femur in lab peak (deg) |  | p=0.02 |
|  | Knee flexion at heel strike (deg) |  | p=0.178 |
| Noehren et al. 2013  PFPS | Hip adduction angle (deg) | Subjects ran along a 25 m run way, in a standard neutral running shoe, at a speed of 3.7 m/s (±5%) striking a force plate at its center.  Kinematic data were collected at 120 Hz with a 6 camera Vicon 512 motion analysis system and low-pass filtered at 8 Hz with a fourth-order zero lag Butterworth filter. Force data was sampled at 1080 Hz and low-pass filtered at 50 Hz with a fourth-order zero lag. | p=0.007 |
|  | Hip internal rotation (deg) |  | p=0.47 |
|  | Rearfoot eversion angle (deg) |  | p=0.1 |
| Peterson et al. 2020 (unpublished data) | Navicular drop | Assessed as the change in height of the navicular tuberosity between neutral calcaneal stance position (a bipedal stance position, where the test leg is held in the STJ neutral position), and a resting bipedal stance position. | MD: 0.12 (-0.40-0.63, p=0.66) |
|  | Foot posture index-6 | Six separate foot posture measurements performed and each assigned a value from -2 to +2, with -2 being highly supinated, 0 being neutral, and +2 being highly pronated. These values are combined to give an overall FPI score, and the foot classified as highly pronated, pronated, neutral, supinated, or highly supinated. | Risk ratio: 1.20 (0.56-2.58, p=0.64) |
| Shen et al. 2019  ITBS | Peak trunk lateral flexion | Running at 3.7m/s. A Kistler force plate sampling at 1000Hz was used to collect force data. Kinematic data were synchronously collected using an 8-camera motion capture system sampling at 100Hz. Five acceptable trials were conducted. | p=0.825 |
|  | Peak trunk inclination angle |  | p=0.168 |
|  | Peak hip abductor moment |  | p=0.179 |
|  | Peak knee external rotation |  | p=0.752 |
| Stefanyshyn et al. 2013  PFPS | Knee abduction impulse (Nm/s) | Running speed was controlled (4.0 ± 0.2 m/s) using 2 photocells, 1.9 m apart, at shoulder height. The 3-dimensional spatial positions of 9 reflective markers were collected using 4 electronically shuttered, high-speed vide cameras. The sampling frequency was set at 200 frames per second, and the exposure time was set at 1/2500 seconds.  Simultaneous 3-dimensional force data were collected using a force platform mounted flush with the floor in the center of a 30-m runway. Force data were sampled at 1000 Hz. | PFPS vs. uninjured cohort p=0.23 PFPS vs matched participants p=0.042 |
| Thijs et al. 2008  PFPS | Vertical plantar peak force underneath hallux (N) | A footscan pressure plate was mounted in the middle of a walkway 15m in length.  Maximum vertical force under hallux, metatarsal heads 1-5, medial heel, and lateral heel, and Relative maximum vertical force (peak force relative to total time of foot contact) | p=0.336 |
|  | Vertical plantar peak force underneath metatarsal I (N) |  | p=0.324 |
|  | Vertical plantar peak force underneath metatarsal II (N) |  | p=0.016  Logistic regression: p=0.037 |
|  | Vertical plantar peak force underneath metatarsal III (N) |  | p=0.026  Logistic regression: p=0.523 |
|  | Vertical plantar peak force underneath metatarsal IV (N) |  | p=0.058 |
|  | Vertical plantar peak force underneath metatarsal V (N) |  | p=0.089 |
|  | Vertical plantar peak force underneath medial heel (N) |  | p=0.213 |
|  | Vertical plantar peak force underneath lateral heel (N) |  | p=0.034  Logistic regression: p=0.872 |
|  | Time to vertical plantar peak force underneath hallux relative to total time of foot contact (s) |  | p=0.798 |
|  | Time to vertical plantar peak force underneath metatarsal I relative to total time of foot contact (s) |  | p=0.251 |
|  | Time to vertical plantar peak force underneath metatarsal II relative to total time of foot contact (s) |  | p=0.693 |
|  | Time to vertical plantar peak force underneath metatarsal III relative to total time of foot contact (s) |  | p=0.776 |
|  | Time to vertical plantar peak force underneath metatarsal IV relative to total time of foot contact (s) |  | p=0.356 |
|  | Time to vertical plantar peak force underneath metatarsal V relative to total time of foot contact (s) |  | p=0.201 |
|  | Time to vertical plantar peak force underneath medial heel relative to total time of foot contact (s) |  | p=0.016 Logistic regression: p=0.35 |
|  | Time to vertical plantar peak force underneath lateral heel relative to total time of foot contact (s) |  | p=0.037  Logistic regression: p=0.048 |
|  | Foot Posture Index-6 | Six separate foot posture measurements performed and each assigned a value from -2 to +2, with -2 being highly supinated, 0 being neutral, and +2 being highly pronated. These values are combined to give an overall FPI score, and the foot classified as highly pronated, pronated, neutral, supinated, or highly supinated. | Pearson’s chi^2^ test: p=0.788 |
| Thijs et al. 2011  PFPS | All measures | Isometric strength (make-method) using a Microfet hand-held dynamomete. 3 trials per muscle group, MVIC for 2-3s, with 15s rest between trials |  |
|  | Hip flexors, force | Seated. Participant pushed maximally against the dynamometer, which was placed on the anterior aspect of the distal thigh, 2 cm proximal to the knee. | p=0.31 |
|  | Hip extensors, force | Prone: subject extended hip, with knee flexed 90^o^, against the dynamometer, which was placed on the posterior aspect of the distal thigh, 2 cm proximal to the popliteal crease. | p=0.4 |
|  | Hip abductors, force | Supine. Participant abducted the leg against the dynamometer, which was placed on the lateral aspect of the distal thigh, 2 cm proximal to the lateral epicondyle of the knee. | p=0.55 |
|  | Hip adductors, force | Supine. Participant adducted the leg against the dynamometer, which was placed on the lateral aspect of the distal thigh, 2 cm proximal to the medial epicondyle of the knee. | p=0.26 |
|  | Hip external rotators, force | Seated. Dynamometer positioned 2cm proximal to medial malleolus. | p=0.61 |
|  | Hip internal rotators, force | Seated. Dynamometer positioned 2cm proximal to lateral malleolus. | p=0.61 |
|  | q-angle, degrees | Bipedal stance. For each leg the midpoint of the patella, the anterior iliac spine (ASIS), and the tibial tubercle were marked. Subsequently, a string was used to connect the midpoint of the patella to the ASIS and to the tibial tubercle, respectively. The Q-angle was measured with a goniometer. | p=0.9 |
| Torp et al. 2018  RRI | Knee flexion strength  (NM/Kg*m) | A hand-held dynamometer. Three 5s MVIC (make-test), 30s rest between trials. | Effect size: -0.08 (-0.68-0.53, p=0.87) |
|  | Knee extension strength |  | Effect size: -0.33 (-0.93-0.29, p=0.21) |
|  | Hip flexion strength |  | Effect size: 0.19 (-0.42-0.79, p=0.43) |
|  | Hip extension strength |  | Effect size: 0 (-0.60-0.6, p=0.94) |
|  | Hip external rotation strength |  | Effect size: -0.25 (-0.85-0.38, p=0.41) |
|  | Hip abduction strength |  | Effect size: -0.18 (-0.79-0.43, p=0.52) |
| Van Der Worp et al. 2016  RRI | Navicular drop | The change in the height of the navicular tuberosity between sitting, a non–weightbearing position (subtalar neutral), and standing, a weight-bearing position with the subtalar joint in relaxed stance. | HR (bivariate): 1 (0.93-1.08, p=0.98)  HR (multivariate): 1 (0.92-1.09, p=0.99) |
|  | 1st MPJ extension | 2 marks were made on the medial and ventral aspects of the MTP1 and 1 mark was made on the medial side of the base of the shaft of the first metatarsal bone. The static weightbearing step-length (30 cm) position, was used to measure the extension of the MTP1, using a goniometer. | HR (bivariate): 1.01 (0.99-1.03, p=0.49)  HR (multivariate): 1 (0.98-1.02, p=0.81) |
| Van Ginckel et al. 2009  Achilles tendinopathy | 79 variables reported in paper. N.B. Only significant variables displayed in this table | Participants ran barefoot at a comfortable self selected speed over a force plate on a 15metre runway. 3 bilateral steps were analysed for kinetic plantar pressure variables. |  |
|  | Anteroposterior displacement of center of force at last foot contact (mm) |  | p=0.008 |
|  | Anteroposterior displacement of center of force at forefoot push off phase (mm) |  | p=0.033 |
|  | Total anteroposterior displacement of center of force (mm) |  | p=0.007  Stepwise linear regression  OR 0.919 (0.859-0.984, p= 0.015 |
|  | Mediolateral force ratio 2 at forefoot flat |  | p=0.009 |
|  | Mediolateral force ratio 2 at foot flat phase |  | p=0.045  Stepwise linear regression  OR 0 (0-0.158, p= 0.016) |
|  | Time to vertical plantar peak force underneath medial heel (s) |  | p=0.032 |
| Wen et al. 1998  RRI | Arch index | The ratio of navicular height to foot length, defined as the distance from the posterior calcaneus to the first metatarsophalangeal joint. | Statistical tests for injured vs. non-injured runners not reported |
|  | Heel valgus | Measured standing, was the angle between a line along the vertical axis of the posterior calcaneus and a second line along the vertical axis of the posterior lower leg. Positive values referred to valgus, negative values to varus |  |
|  | Tubercle-sulcus angle | Measured with the subject seated and knees flexed to 90° as the angle between a vertical line through the centre of the patella and a line from the centre of the patella to the centre of the tibial tubercle. |  |
|  | Knee varus | Assessed standing with varus (positive values) as the distance between the medial femoral epicondyles with the medial malleoli touching and valgus (negative values) as the distance between the medial malleoli with the medial femoral epicondyles touching. |  |
|  | Leg length difference | measured supine with leg length as the distance from the anterior superior iliac spine to the inferior of the medial malleolus |  |
| Winter et al. (2019)  RRI | Speed (km/h) | A wireless tri-axial accelerometer (52x30x13mm, mass 23g, 16 bit resolution, 250Hz sampling; SABEL Labs, Brisbane Aus). Attached at the level of L5/S1 vertebrae. Data were analysed using a custom written program in MATLAB R2014a (The Mathworks Inc., Natick, MA), filtered using a zero-lag fourth order low-pass Butterworth filter with a cut-off frequency of 25Hz. Spatiotemporal variables calculated were contact time, flight time, and step frequency from vertical acceleration data. Dynamic loading measures during stance were determined using peak positive vertical and peak negative anteroposterior accelerations. Variability was measured using step and stride regularity calculated in each acceleration axis using unbiased autocorrelation procedures. | Slow/intermediate/advanced runners: NS |
|  | Contact time (ms) |  | Slow/intermediate/advanced runners: NS |
|  | Flight time (ms) |  | Slow runners: p<0.05  Intermediate/advanced runners: NS |
|  | Step frequency |  | Slow runners: p<0.05  Intermediate/advanced runners: NS |
|  | Peak VT (g) |  | Slow/intermediate/advanced runners: NS |
|  | Peak AP (g) |  | Slow/intermediate/advanced runners: NS |
|  | VT RMSR (arb. Units) |  | Slow/intermediate/advanced runners: NS |
|  | ML RMSR (arb. Units) |  | Slow/intermediate/advanced runners: NS |
|  | AP RMSR (arb. Units) |  | Slow/intermediate/advanced runners: NS |
|  | Step reg VT |  | Slow runners: p<0.05  Intermediate/advanced runners: NS |
|  | Stride reg VT |  | Slow/intermediate/advanced runners: NS |
|  | Step reg ML |  | Slow/intermediate/advanced runners: NS |
|  | Stride reg ML |  | Slow/intermediate/advanced runners: NS |
|  | Step reg AP |  | Slow/intermediate/advanced runners: NS |
|  | Stride reg AP |  | Slow/intermediate/advanced runners: NS |
| Zifchock et al. 2007  RRI | Hip abduction strength, kg/Bw | MVIC following Leetun et al. (2004) procedures. This was done with a hand-held dynamometer. | p-value not reported |
|  | Hip external rotation strength, kg/bw |  | p-value not reported |
|  | Knee Valgus Angle, deg | Frontal plane knee varus angle determined during the standing calibration trial for motion capture system. | p-value not reported |
|  | Q-angle, deg | Measured in supine using a standard goniometer. | p-value not reported |
|  | Hip internal rotation, deg | Measured in prone with participants knee flexed to 90^o^. | p-value not reported |
|  | Arch height index | Measure of the height of the dorsum at 50% the total foot length, normalized to the length of the foot from the posterior heel to the first metatarsal joint | p-value not reported |
|  | impact force along shank | Subjects ran along a 25m runway at a speed of 3.7m/s ±5%, striking the centres of two forceplates with consecutive foot strikes. Kinematic data were collected with a 6-camera, VICON motion analysis system  The Symmetry Angle (SA) was used to compare asymmetry levels between the injured and uninjured subjects. The SA is a measure related to the angle formed when two values (Xright, Xleft) are plotted in two-dimensional space. The deviation of this angle from a 45º axis of perfect symmetry is an indicator of the asymmetry between the two values. The value is then normalized to a maximum deviation of 90º from the axis of perfect symmetry. Therefore an SA value of 0% indicates perfect symmetry, and 100% represents two values that are perfectly out-of-phase. The SA value is calculated using the equation: SA = (45° – arctan(Xleft/Xright))/90° * 100% | p-value not reported |
|  | instantaneous loading rate along shank(BW) |  | p-value not reported |
|  | peak shock along shank (BW), g |  | p-value not reported |
|  | knee joint stiffness(N/kg-deg) |  | p-value not reported |
|  | avg velocity of hip adduction, deg/sec |  | p-value not reported |
|  | avg velocity of hip internation rotation, deg/sec |  | p-value not reported |
|  | Avg velocity of knee adduction, deg/sec |  | p-value not reported |
|  | Avge velocity of rearfoot eversion, deg/sec |  | p-value not reported |
|  | Symmetry angle |  | p-value not reported |
| ERLLP = exercise related lower leg pain, NCSP = neutral calcaneal stance position, RCSP = resting calcaneal stance position, RRI = running-related injury, ROM = range of motion, STJ = subtalar joint, HR = hazard ratio, MVIC = maximal voluntary isometric contraction, MD = Mean difference, 1RM = one repetition maximum, H1 = medial heel, H2 = lateral heel, M = metatarsal head, T1 = hallux, FMC = first metatarsal contact, FFF = forefoot flat, ICP = initial contact phase, COF = medio-lateral displacement of the centre of force, Vx = velocity of mediolateral displacement of COF, Yx = velocity anterior-posterior displacement of the COF, dy = anterio-posterior displacement of the COF, ImpulsM5 = absolute force-time integral beneath M5, pMaxM5 = peak force beneath M5, BW = body weight, ITBS = iliotibial band (friction) syndrome, PFPS = patellofemoral pain syndrome, F = force, T1 = hallux, M1-5 = 1^st^ to 5^th^ metatarsal heads, H1 = medial heel, H2 = lateral heel, Relt = time to vertical peak force relative to time of foot contact, RPE = rating of perceived exertion, SA = symmetry angle, BW = body weight, LFE = lateral femoral epicondyle, NS = non-significant; N/A = not applicable  *Data presented for participants meeting eligibility criteria for this review – results differ from published report **Data re-analysed for this review using review manager 5 or SPSS | | | |
